# Nomograms Based on Serum *N*-glycome for Diagnosis of Papillary Thyroid Microcarcinoma and Prediction of Lymph Node Metastasis

**DOI:** 10.3390/curroncol29090474

**Published:** 2022-08-23

**Authors:** Zejian Zhang, Zhen Cao, Rui Liu, Zepeng Li, Jianqiang Wu, Xiaoli Liu, Mengwei Wu, Xiequn Xu, Ziwen Liu

**Affiliations:** 1 Medical Research Center, State Key Laboratory of Complex Severe and Rare Diseases, Peking Union Medical College Hospital, Chinese Academy of Medical Sciences and Peking Union Medical College, Beijing 100730, China; 2 Department of General Surgery, Peking Union Medical College Hospital, Chinese Academy of Medical Sciences and Peking Union Medical College, Beijing 100730, China; 3 Department of Clinical Laboratory, Peking Union Medical College Hospital, Chinese Academy of Medical Sciences and Peking Union Medical College, Beijing 100730, China; 4 Department of Hernia and Abdominal Wall Surgery, Beijing Chao-Yang Hospital, Capital Medical University, Beijing 100043, China

**Keywords:** papillary thyroid microcarcinoma, lymph node metastasis, serum glycomics, capsular invasion, nomogram

## Abstract

Non-invasive biomarkers for the diagnosis and prognosis of papillary thyroid microcarcinoma (PTMC) are still urgently needed. We aimed to characterize the *N*-glycome of PTMC, and establish nomograms for the diagnosis of PTMC and the prediction of lymph node metastasis (LNM). *N*-glycome of PTMC (LNM vs. non-LNM, capsular invasion (CI) vs. non-CI (NCI)) and matched healthy controls (HC) were quantitatively analyzed based on mass spectrometry. *N*-glycan traits associated with PTMC/LNM were used to create binomial logistic regression models and were visualized as nomograms. We found serum *N*-glycome differed between PTMC and HC in high-mannose, complexity, fucosylation, and bisection, of which, four *N*-glycan traits (TM, CA1, CA4, and A2Fa) were significantly associated with PTMC. The nomogram based on four traits achieved good performance for the identification of PTMC. Two *N*-glycan traits (CA4 and A2F0S0G) showed strong associations with LNM. The nomogram based on two traits showed relatively good performance in predicting LNM. We also found differences between CI and NCI in several *N*-glycan traits, which were not the same as that associated with LNM. This study reported serum *N*-glycosylation signatures of PTMC for the first time. Nomograms constructed from aberrant glycans could be useful tools for PTMC diagnosis and stratification.

## 1. Introduction

Papillary thyroid cancer (PTC) is a common endocrine malignancy, with the incidence rising steadily over the past three decades [1]. The incidence of PTC increased by more than 35% each year in China from 2000 to 2010 [2] A recent study showed that the age-standardized incidence rate of female PTCs during 2008–2012 per 100,000 person-years ranged from 5.3 cases in north-western Europe to 143.3 cases in South Korea. The incidence rates of male PTCs per 100,000 person-years ranged from 1.2 cases in Thailand to 30.7 cases in South Korea [3]. Papillary thyroid microcarcinoma (PTMC) is defined as PTC with a maximum tumor diameter of 10 mm or less [4], and surgeons usually distinguish between PTMC and PTC by directly measuring the maximum diameter of the tumor on the ultrasound image. PTMC accounts for approximately 35–70% of all PTC cases, and its incidence has dramatically increased in both males and females [5]. With universal uses of ultrasonography and fine-needle aspiration biopsy (FNAB), it was reported that the incidence of PTMC has increased to 441% within just two decades [6,7]. Though most PTMCs have an indolent clinical course with excellent prognosis, a subset of these patients has been shown to harbor aggressive features, such as cervical lymph node metastasis (LNM), extrathyroidal extension, lymphovascular invasion, or distance metastasis that can lead to recurrence and a decreased survival time [8,9,10]. Thus, the aggressive biological behavior of PTMC indicates that PTMC may not be just a simple occult cancer that only differs from larger PTC in tumor size. However, this issue remains controversial. For the affiliation between PTMC and PTC, some researchers consider PTMC to be similar to PTC in histology and biology. Park et al. found that PTMC and PTC patients were similar in demographics, clinical characteristics, BRAF^V600E^ mutational status, and the expression of some intracellular molecules [11]. Timler et al. indicated no difference in cyclin D, which played a role in tumor progression, between PTC larger than 10 mm and PTMC [12], whereas others consider that PTMC is not the “early stage” of PTC. Therefore, targeted research of PTMC will be important for understanding its pathogenesis, and discovering specific approaches for its diagnosis and prognosis.

Clinically, FNAB with cytopathological examination is an effective tool for the diagnosis of thyroid cancer (TC). However, it has limited value for tumors smaller than 10 mm. The American Thyroid Association (ATA) guidelines recommend against the use of FNAB for thyroid nodules smaller than 10 mm, even those with highly suspicious ultrasound patterns [13,14]. Additionally, FNAB cannot obtain sufficient tissue for cytological analysis due to its small size, and tends to result in a high false-negative rate. Therefore, effective diagnostic approaches for PTMC are urgently needed. On the other hand, depending on the different conditions of each patient, surgeons have several options, from active surveillance (AS) to total thyroidectomy with cervical lymph node dissections [11]. PTMC patients with high-risk features are not suitable for AS, and need operation [15]. The preoperative identification of patients with clinical LNM is critical for the selection of treatment [15,16]. However, the rates of misdiagnosis and missed diagnosis are high in the identification of LNM due to atypical clinical manifestations of PTMC [16]. The sensitivity of ultrasound detection of LNM is only 20–38% [17]. This situation may cause the best timing of treatment to be missed, affecting the prognosis. Moreover, though several studies have reported that some risk factors, including age, sex, and capsular invasion (CI), might have predictive value for LNM [18,19,20], these risk factors are debatable [21]. Approaches for the preoperative prediction of LNM are still needed.

Glycosylation is one of the most common and important post-translational modifications [22]. Glycosylation analysis in the broader context of cancer can provide meaningful insight into the mechanisms of cancer progression. Furthermore, serological glycomic profiling is a rising non-invasion approach for the discovery of potential biomarkers for cancer diagnosis and disease surveillance in recent years [23,24]. A growing body of studies suggests that total serum/plasma *N*-glycome has great potential as a biomarker for the diagnosis of cancers, including PTC [25,26,27]. In response to the current controversy over whether the pathological mechanisms of PTMC and PTC are the same, we conduct this PTMC glycomics study on the basis of our previous PTC glycomic study [25], hoping to provide new clues for their pathological mechanism and find PTMC-specific diagnostic biomarkers. Moreover, whether serum *N*-glycome has potential as a biomarker for predicting LNM of PTMC still needs to be investigated.

In this study, we investigated serum protein *N*-glycomic features of healthy controls (HC) and PTMC patients consisting of LNM and non-LNM (NLNM) using matrix-assisted laser desorption/ionization time-of-flight mass spectrometry (MALDI-TOF MS). We aimed to explore non-invasion glycan biomarkers and establish nomograms based on glycans for PTMC diagnosis and stratification. Additionally, *N*-glycome of CI and non-CI (NCI) in PTMC was analyzed to explore associations between CI and LNM. Moreover, based on our previous PTC glycomic study, *N*-glycome profiles between PTMC and PTC were also investigated to discover their difference.

## 2. Materials and Methods

### 2.1. Participants

The study enrolled 100 patients diagnosed with PTMC, and 80 HC volunteers. The serum specimen of each individual was consecutively collected from Peking Union Medical College Hospital (PUMCH, Beijing, China) between January 2021 and June 2021. The inclusion criteria for the PTMC group were: (a) the maximal diameter of the thyroid nodule ≤ 10 mm on the ultrasound images; (b) age ≥ 18 years; (c) diagnosed based on postoperative pathology, and had no distant metastasis. In the HC group, all volunteers should have normal thyroid function, thyroid ultrasonography, and biochemical and blood coagulation parameters. The exclusion criteria were: (a) a history of blood and infectious diseases, diabetes, impaired glucose tolerance, or autoimmune disorders that required systemic immunosuppressive therapy; (b) a history of pituitary disease or other cancers; (c) a previous history of thyroid or malignancy operations. The clinical and pathological characteristics of the cohort are presented in Table 1. 

### 2.2. Serum N-glycome Detection and MS Data Processing

A total of 180 serum samples from the cohort (100 PTMC and 80 HC) and 17 quality control samples (5 blanks and 12 replicated serum standards) were randomly distributed into three 96-well plates. According to the protocol described previously, serum glycoproteins from the samples were enzymatically treated to release *N*-glycans [28]. Briefly, 10 μL of 2% sodium dodecyl sulfonate was added to 5 μL of serum specimen, and then the mixture was incubated for 10 min at 60 °C. The process of *N*-glycan release was performed by the addition of 10 μL of the mixture (2% Nonidet P-40, 2.5 × phosphate-buffered salines, and 1 U PNGase F), followed by incubation at 37 °C overnight. During the derivation procedure, sialic acid residues at the nonreducing ends of the glycan were derivatized to stable end-products (α-2,3-linked sialic acids were lactonized, and α-2,6-linked sialic acids were ethyl-esterified), permitting mass-based distinction among the sialic-acid linkage variants [28]. In short, 1 μL of the release mixture was added to 20 μL of derivatization reagent (250 mM 1-ethyl-3-(3-dimethylaminopropyl)-carbodiimide and 250 mM hydroxybenzotriazole in ethanol), followed by incubation for 1 h at 37 °C. Detailed information on the glycan release and derivatization process is provided in the Supplementary Materials. The derivatized *N*-glycans were then purified by hydrophilic interaction liquid chromatography solid-phase extraction (HILIC-SPE) micro-tips, and were eventually eluted with MilliQ water according to the previously reported method [26,29,30]. Thereafter, 1 μL of purified glycans were mixed with 1 μL of the matrix (5 mg/mL super-2,5-dihydroxybenzoic acid in 50% acetonitrile with 1 mM NaOH) on a target plate and were air-dried at room temperature. The state-of-the-art and high-resolution MS platform (rapifleXtreme MALDI-TOF (Bruker Daltonics, Bremen, Germany) was employed to measure the purified glycans. The instrument was equipped with a Smartbeam-3D laser and was controlled by flexControl 4.0 software (Bruker Daltonics). The equipment was calibrated by external calibrants (Bruker Peptide Calibration Standard II). The mass range of measurements was set to 1000–5000 m/z. For each spot, 5000 laser shots were accumulated in random walking mode at a laser frequency of 5000 Hz.

The raw MS data were processed according to the workflow reported in the previous study [28,29,30]. The raw MS data had been deposited in GlycoPOST (ID: GPST000252, https://glycopost.glycosmos.org accessed on 22 August 2022) [31]. Briefly, the raw data were smoothed, baseline-subtracted, and transformed to .XY files using the software of flexAnalysis (Bruker Daltonics, Bremen, Germany). The transformed .XY files were then re-calibrated using internally developed MassyTools software (version 0.1.8.1.2) [32]. The peaks of MS spectra were manually assigned to *N*-glycan compositions using the GlycoPeakfinder tool of GlycoWorkbench [33,34]. The *N*-glycan compositions were also confirmed by previous literature [25,28,29]. A detailed process for glycan identification is provided in the Supplementary Materials. Finally, the composition list of 131 putative *N*-glycan compositions was generated for subsequent targeted data extraction. The peak intensities of putative glycan composition were extracted as peak areas (background-corrected) by the composition list and MassyTools. The extracted data were further processed in Microsoft Excel. In the furthering process, 35 out of 131 glycan compositions were excluded after applying the cut-offs of S/N > 9, ppm error < 20, QC score < 25%, and the minimum percentage (>50%) of presence in all spectra of PTMC, HC, or quality control serum samples [29]. After the curation, 96/131 *N*-glycan compositions met the quality criteria and were included in quantitative analysis (Supplementary Table S1). Thereafter, the sum of areas per spectrum was rescaled to one. To explore the exact role of a group of *N*-glycans with similar glycan structures and to explain their biological effects, 91 derived *N*-glycan traits were calculated from the 96 directly detected *N*-glycan traits based on their common structural characteristics. The formulas used for the calculation of derived glycan traits are given in Supplementary Table S2. The calculations were implemented in RStudio. Aberrant derived glycan traits indicate that changes in glycosylation are shared by a series of structurally related N-glycans [29].

The average value, standard deviation (SD), and coefficient of variance (CV) of the 12 replicated standard samples were calculated for directly detected and derived glycan traits to evaluate the quality of the data (Supplementary Table S3).

### 2.3. Statistical Analysis

Categorical variables were presented as frequencies and percentages. Continuous variables were presented as means ± standard deviations (Table 1). Comparisons were performed for the derived N-glycan traits among subgroups (PTMC vs. HC, LNM vs. NLNM, CI vs. NCI) using the nonparametric Mann–Whitney–Wilcoxon test. Multiple testing correction was conducted to adjust the significance threshold (*p* = 0.05/91, “91” is the number of derived glycan traits). The receiver operating characteristic (ROC) curve was used to evaluate the sensitivity, specificity, and accuracy of each glycan trait. The derived glycan traits that showed diagnostic or predictive potential in univariate analysis were entered into multivariate analysis. Statistically significant derived glycan traits in multivariate analysis (cutoff value of significance: *p* < 0.05) were used to create visual nomograms, which were tested for goodness of fit using Hosmer–Lemeshow test. The ROC curves were used to evaluate predictive performance. Statistical analyses were performed by SPSS statistics (version 25.0), GraphPad Prism software (version 8.4.3), and R software (version 4.0.3). The GlycoWorkbench software was used for the annotation of glycan structures.

## 3. Results

**Figure 1:**
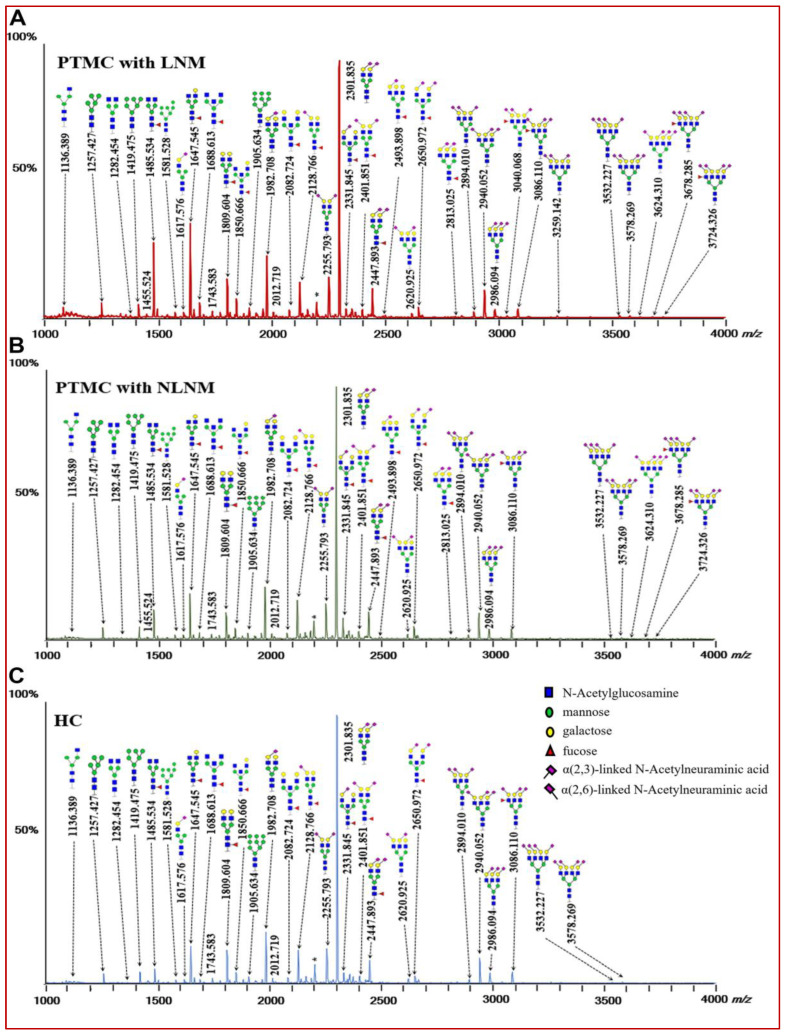
The representative matrix-assisted laser desorption/ionization time-of-flight mass spectrometry (MALDI-TOF-MS) spectra of serum protein *N*-glycome for (**A**) PTMC with LNM, (**B**) PTMC with NLNM, and (**C**) HC. Spectra were recorded in positive-ion reflection mode on a Bruker rapifleXtreme mass spectrometer. Major *N*-glycan peaks are annotated and assigned to compositions, and the presence of structural isomers cannot be excluded. The asterisks (*) represent by-products. Abbreviations: PTMC, papillary thyroid microcarcinoma; LNM, lymph node metastasis; NLNM, non-lymph node metastasis; HC, healthy control.

Serum *N*-glycans were detected by MALDI-TOF MS. Ninety-six directly detected *N*-glycan traits passed the quality criteria (Supplementary Table S1). Examples of annotated spectra of serum *N*-glycomes from PTMC with LNM, PTMC without LNM, and HC are shown in Figure 1, indicating the differences in peak patterns among these groups. Ninety-one derived *N*-glycan traits were generated from the ninety-six directly detected *N*-glycan traits based on the similar structural features of glycan traits (Supplementary Table S2). The characteristics of derived glycan traits include number of antennae (A), fucosylation (F), bisection (B), galactosylation (G), and α-2,3-linked (L) and α-2,6-linked sialylation (E). The consistent quality of data and the repeatability of the overall approach were evaluated by replicated serum standard samples. The average CV of directly detected glycan traits (top 20) and all derived glycan traits was 3.67% and 3.42%, respectively (Supplementary Table S3). Since the derived *N*-glycan traits could combine the exact effects of individual glycans sharing similar glycan structures, help the interpretation of the biological effects, and have better repeatability than the individual glycan compositions from which they were calculated [35], we mainly focused on the derived *N*-glycan traits for the subsequent analysis in the study.

### 3.1. Identification of Serum N-glycomic Features for Discriminating PTMC from HC

Several glycomic features were found to differ between the groups of PTMC and HC. High-mannose glycans (TM) and the ratio of high-mannose to hybrid glycans (MHy) were upregulated in PTMC compared to that in HC (Table 2, Supplementary Table S4). Serum *N*-glycome between PTMC and HC showed changes in the antennary (A) of complex-type glycans. Mono-antennary and tetra-antennary *N*-glycans within complex type (CA1 and CA4) were increased in PTMC compared to HC (Table 2, Supplementary Table S4). In addition, PTMC displayed lower levels of fucosylation than HC, particularly in polyfucosylation (CFa and A2Fa; Table 2). In addition to fucosylation changes, PTMC patients showed higher levels of bisecting GlcNAc than HC, mainly because of the increase of bisection within non-fucosylated diantennary complex type (A2F0B and A2F0SB) (Table 2, Supplementary Table S4).

**Figure 2:**
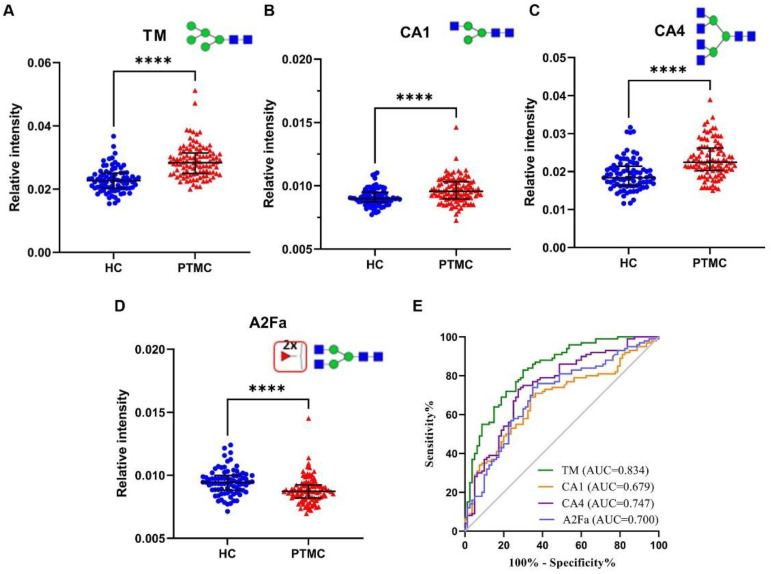
The scatter plots and ROC curve analysis of four prominently different derived *N*-glycan traits between PTMC and HC groups. (**A**) scatter plot for TM, (**B**) scatter plot for CA1, (**C**) scatter plot for CA4, (**D**) scatter plot for A2Fa, and (**E**) ROC curve analysis for four differential expressed derived glycan traits. **** represents *p*-value < 0.0001 (after multiple testing correction). Notes: mannose, green circle; fucose, red triangle; GlcNAc, blue square. Abbreviations: ROC, receiver operating characteristic; PTMC, papillary thyroid microcarcinoma; HC, healthy control; AUC, area under the curve.

The associations between serum glycan traits and PTMC were further explored by multivariable logistic regression. TM, CA1, CA4, and A2Fa (*p* = 0.002, *p* = 0.042, *p* = 0.000, *P* = 0.010, respectively) were found to be strongly associated with PTMC (Table 2, Figure 2). ROC curves were also used to assess the selected four glycan traits. The areas under the curves (AUCs) of TM, CA1, CA4, and A2Fa were 0.834, 0.679, 0.747, and 0.700 in discriminating PTMC from HC (Figure 2), respectively. These results indicate that nomograms based on the four glycan traits might be useful tools for the diagnosis of PTMC, as further investigated below.

### 3.2. Identification of Serum N-glycomic Features of PTMC Patients with LNM

We investigated the differentially expressed derived *N*-glycan traits between PTMC with LNM and NLNM. We found the ratio of high-mannose to hybrid glycans (MHy) and the average number of mannoses on high-mannose-type glycans (MM) was upregulated in the LNM compared to that in NLNM (Table 3, Supplementary Table S4). The differences in the antennary were also found: triantennary and tetra-antennary *N*-glycans within complex type (CA3 and CA4) were increased in LNM compared to NLNM, with the concomitant decrease in diantennary species (CA2; Table 3, Supplementary Table S4). In addition, LNM showed a lower level of galactosylation within non-fucosylated and non-sialylated diantennary complex-type (A2F0S0G; Table 3, Supplementary Table S4). Altered sialylation was also discovered in LNM compared to NLNM. Sialylation per antenna within triantennary (A3S) was higher in LNM than in NLNM (Table 3, Supplementary Table S4).

**Figure 3:**
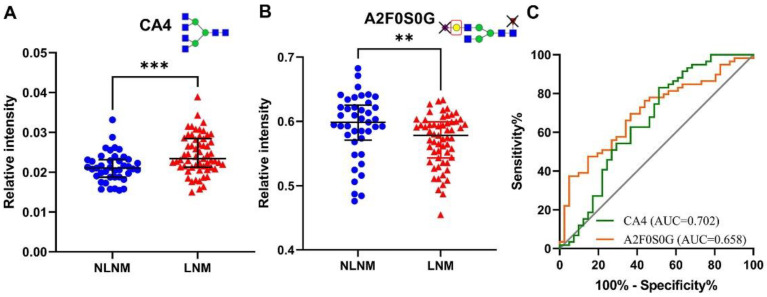
The scatter plots and ROC curve analysis of two prominently different derived *N*-glycan traits between PTMC with LNM and with non-LNM groups. (**A**) scatter plot for CA4, (**B**) scatter plot for A2F0S0G, and (**C**) ROC curve analysis for two differential expressed derived glycan traits. *** represents *p*-value < 0.001, ** represents *p*-value < 0.01 (after multiple testing correction). Notes: mannose, green circle; GlcNAc, blue square; fucose, red triangle; galactose, yellow circle; sialic acid, purple diamond. Abbreviations: ROC, receiver operating characteristic; PTMC, papillary thyroid microcarcinoma; LNM, lymph node metastasis; AUC, area under the curve.

The associations of serum glycan traits with LNM in PTMC were further explored by multivariable logistic regression. CA4 and A2F0S0G (*p* = 0.001, *p* = 0.011, respectively) were strongly associated with LNM (Table 3, Figure 3). The AUCs of CA4 and A2F0S0G were 0.702 and 0.658 in differentiating LNM from NLNM (Figure 3). The two glycan traits were included for further establishment of the nomogram.

### 3.3. Establishment of Nomograms Based on Glycan Traits for the Diagnosis of PTMC and Preoperative Prediction of LNM

**Figure 4:**
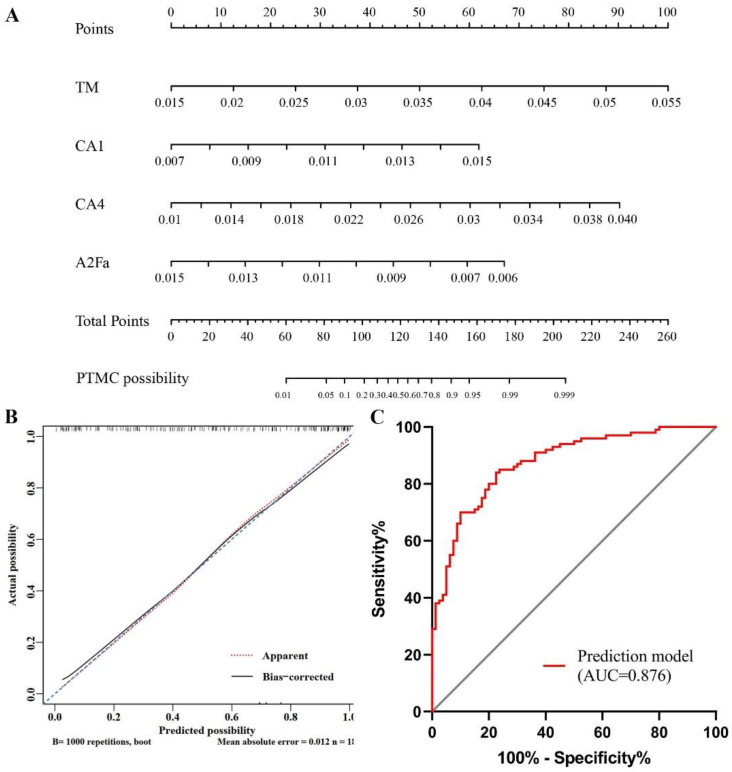
The binomial logistic regression model constructed from glycans for the diagnosis of PTMC. (**A**) The visualized nomogram of the logistic regression model; (**B**) Calibration curve of the diagnosis nomogram. The 45° dashed line represents the ideal predictions. The solid line shows a bias-corrected curve. The dotted line shows the nomogram probability; (**C**) ROC curve of the nomogram for the diagnosis of PTMC. PTMC, papillary thyroid microcarcinoma; ROC, receive operating characteristic; AUC, area under the curve.

**Figure 5:**
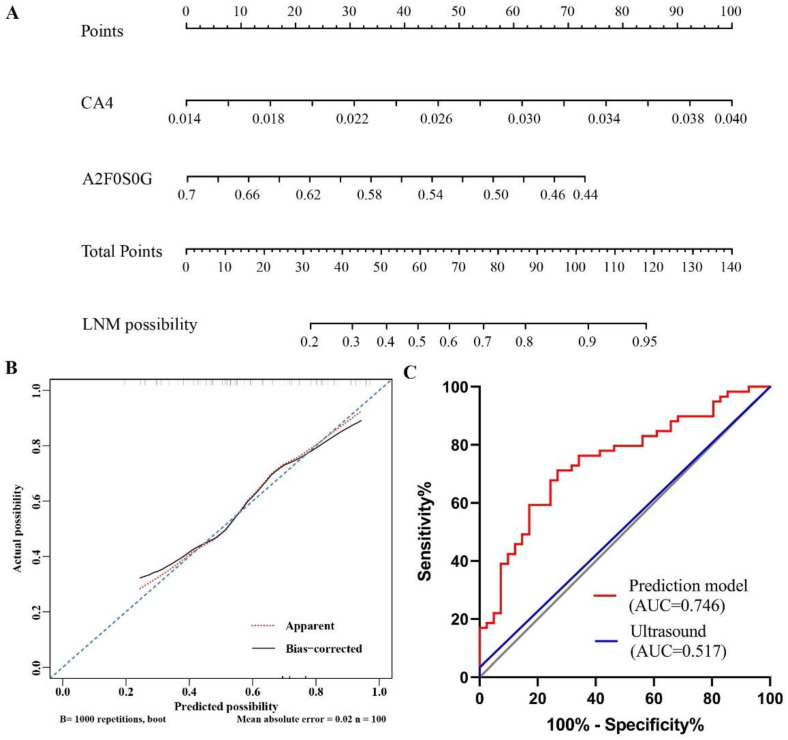
The ultrasound and the binomial logistic regression model constructed from glycans for preoperative prediction of LNM in papillary thyroid microcarcinoma. (**A**) The visualized nomogram of the logistic regression model; (**B**) Calibration curve of the nomogram. The 45° dashed line represents the ideal predictions. The solid line shows a bias-corrected curve. The dotted line shows the nomogram probability; (**C**) ROC curves of the nomogram and ultrasound for predicting the probability of papillary thyroid microcarcinoma with LNM. LNM, lymph node metastasis; ROC, receive operating characteristic; AUC area under the curve.

Based on the four *N*-glycan traits (TM, CA1, CA4, and A2Fa) that were significantly associated with PTMC in multivariate analysis, the logistic regression models were created and visualized as nomograms to provide a quantitative method for the diagnosis of PTMC (Figure 4A). A Hosmer–Lemeshow goodness-of-fit test was used to confirm the consistency of the nomogram (χ^2^ =2.012, df = 8, *p* =0.9806). The calibration curve indicated a satisfying agreement between predictions performed by the nomogram and actual outcomes in the cohort (Figure 4B). The AUC of the model was 0.876 (95% CI, 0.819-0.920) (Figure 4C). The optimal cutoff value for diagnosis of PTMC is 0.746, which was determined by the Youden method (sensitivity, 84.0%; specificity, 77.5%; negative predictive value, 82.4%; positive predictive value, 79.5%). The existing method for detecting LNM is ultrasound, which has a sensitivity of 20–38% [17]. Considering that CA4 and A2F0S0G showed strong associations with LNM in PTMC, we built a nomogram for the preoperative prediction of LNM in PTMC based on the two glycan traits (Figure 5A). The consistency of the nomogram was assessed by the Hosmer–Lemeshow goodness-of-fit test (χ^2^ =7.145, df = 8, *p* = 0.516). The calibration curves of the model demonstrated a satisfying agreement between nomogram and observed outcomes (Figure 5B). The AUC of the model was 0.746 (95% CI, 0.649–0.828) (Figure 5C). The optimal cutoff value of the nomogram for the prediction of LNM was 0.534 (sensitivity, 71.2%; specificity, 73.2%; negative predictive value, 79.2%; positive predictive value, 63.8%). Additionally, our results indicated that the AUC value of ultrasound in predicting metastasis was 0.517 based on the clinical and ultrasound data of patients in our study.

### 3.4. Association between Serum N-glycomes and CI of PTMC

The differential levels of derived *N*-glycan traits between CI and NCI in PTMC were explored. The galactosylation within the all-complex-type (CG) was upregulated in CI compared to that in NCI, which was mainly due to the increase of galactosylation of the biantennary glycans (A2G, A2FG, A2S0G, and A2FS0G; Table 4, Supplementary Table S4). Furthermore, the level of sialylation within complex-type glycans (CS) was higher in CI than that in NCI, which was mainly due to the increase of α-2,6-linked sialylation of fucosylated biantennary glycans in CI (A2FS, A2FE; Table 4, Supplementary Table S4). Of note, serum galactosylation levels are elevated in the group of PTMC with CI (compared with NCI) and decreased in the group of PTMC with LNM (compared with NLNM), shown in Table 3 and Table 4.

### 3.5. Identification of serum N-glycome differences between PTC and PTMC

Aberrant *N*-glycans in PTC patients (compared to HC) discovered in our previous study [25] and dysregulated *N*-glycans in PTMC patients (compared to HC) found in the present study were compared. We found three glycan traits (CA4, CFa, and A2Fa) were both dysregulated in PTC and PTMC. Five glycan traits (TM, MHy, CA1, A2F0B, and A2F0SB) were only altered in PTMC. Additionally, altered galactosylation, sialylation, and linkage-specific sialylation were only found in PTC. These findings showed that the serum *N*-glycomes of PTMC and PTC are different. Moreover, we screened out four potential diagnostic biomarkers for PTMC (TM, CA1, CA4, and A2Fa) in the present study (Figure 2 and Figure 4). Two of them (TM and CA1) are specific diagnostic biomarkers for PTMC, whereas the left two (CA4 and A2Fa) were also found useful in PTC diagnosis in our previous study [25].

Furthermore, we compared the *N*-glycomes of PTMC and PTC (diameter > 10 mm). The *N*-glycomic data of PTC patients (diameter > 10 mm, n = 32) from our previous study [25] and that of PTMC patients (n = 32) from this study were batch-corrected and age- and sex-matched, and then compared. We found the ratio of high-mannose to hybrid glycans (MHy) was upregulated in the PTMC compared to that in PTC (diameter > 10 mm). PTC (diameter > 10 mm) patients showed higher levels of fucosylation in tetraantennary species (A4L0F). In addition to fucosylation differences, PTMC patients displayed a higher galactosylation of tetra-antennary glycans (A4G) compared to PTC (diameter > 10 mm). Furthermore, the level of sialylation within tetraantennary glycans (A4S) was higher in PTMC than that in PTC (diameter > 10 mm), which was mainly due to the increase of α2,3-linked sialylation and α2,6-linked sialylation of tetra-antennary glycans (A4L, A4FGL, A4E, A4F0E, and A4FGE).

## 4. Discussion

Apart from the cancer genome, deciphering changes in protein glycosylation is vital not only for understanding the mechanisms of tumor occurrence and progression, but also to explore novel biomarkers for diagnosis and prognosis. Despite significant advances in thyroid cancer health care systems, and the mortality rate remaining stable, deeper insight into the underlying mechanisms and discovering the diagnostic and prognostic biomarkers are crucial for thyroid cancer. The affiliation between PTMC and PTC remains a point of controversy due to the aggressive biological behavior and good prognosis of PTMC. So far, several studies regarding the aberrant glycosylation of PTC have been reported in tissues, plasma, and IgG. Due to the underestimation of PTMC in practice, little is known about the *N*-glycomic profiles of PTMC at present. Hence, this study analyzed the released *N*-glycans from serum samples of HC and PTMC (we also analyzed subgroups of NLNM, LNM, NCI, and CI) using MALDI-TOF MS to explore *N*-glycomic features and potential biomarkers for PTMC diagnosis and stratification.

The present study represents the first comprehensive analysis of serum *N*-glycan profiles in PTMC. Our data demonstrate that PTMC patients have elevated levels of high-mannose, complexity, and bisected *N*-glycans, and decreased levels of fucosylation, compared with HC. Our investigation of the dysregulation of *N*-glycan patterns in PTMC may point to pathophysiological processes involving multiple proteins, as we discuss below. Early studies showed that the high-mannose glycans and complex-type glycans were increased in breast cancer progression, and glycans took part in cancer progression phases, such as cell proliferation signaling, activating invasion and metastasis, and tumor-promoting inflammation [36,37,38]. Chik et al. have shown high-mannose structures as the most abundant glycan in colorectal cancer cell lines and tissues [39]. The abundance of high-mannose and complex type *N*-glycans was also increased in our study, which indicated that these glycan structures might be strongly associated with the occurrence and development of PTMC. These findings together with our observations suggest increased levels of high-mannose and complex-type glycans to be key molecular features in PTMC. Additionally, the aberrant expression of bisecting GlcNAc has been observed in many cancer cells, such as breast, pancreatic, and colorectal cancer, and plays a regulatory role in tumor development by altering *N*-glycans in adhesion molecules and the extracellular matrix [40]. In the present study, we also identified aberrant bisecting GlcNAc in PTMC compared to HC. We guess that the obvious abnormal bisecting GlcNAc in PTMC might play an important role in tumorigenesis and progression by altering adhesion molecules and remodeling the extracellular matrix. Fucosylation is an important mode of posttranslational glycosylation event that regulates cancer signaling processes including metastasis and epithelial-to-mesenchymal transition, and is regulated by various fucosyltransferases. FUT3, 4, and 6 are responsible for antennary fucosylation. FUT3 is involved in tumorigenesis, proliferation, and migration of pancreatic cancer [41]. FUT4 is correlated with higher metastatic potentials in lung cancer [42]. FUT6 is associated with metastatic colorectal cancer [43]. In particular, fucosylation has been studied as a potential marker for several different cancers, such as hepatocellular, pancreatic, and lung cancer. These findings could help to explain the underlying mechanism of dysregulated CFa (difucosylation) and A2Fa (difucosylation) in PTMC in our study. Interestingly, four derived glycan traits (CFa, A2Fa, A2L, and A2GL) were found to show significant changes in plasma *N*-glycome patterns of thyroid cancer compared to HC in our previous study, which was partially consistent with our findings in PTMC serum [25].

Though the PTMC-associated glycan traits were identified, the diagnostic accuracy of each *N*-glycan trait was underpowered. Then, we screened out four derived glycan traits (TM, CA1, CA4, and A2Fa) which were significantly associated with PTMC, based on which, a nomogram was established as a model for the diagnosis of PTMC. The AUC value of our nomogram was greater than 0.8, and has superior accuracy. Patients with a total point value of more than 0.746 (cutoff) are more likely to be diagnosed with PTMC. Hence, this nomogram can provide surgeons with a quantitative tool for PTMC diagnosis, assisting existing approaches (ultrasound and FNAB).

Considering the good prognosis and indolent behavior of PTMC, the necessity of operation is questioned by certain surgeons and patients. Nevertheless, PTMC patients with central LNM need to undergo an operation. Cervical LNM is present in 17–64% of PTMC. Ultrasound is not sensitive for the detection of LNM, which is mainly due to the location of lymph nodes and interference from vessels and trachea [44]. This dilemma could cause a delayed timing of operation. Thus, we aimed to screen out glycan biomarkers for preoperatively identifying LNM in PTMC by investigating the associations between *N*-glycan and LNM. We found an increased abundance of high-mannose (MHy, MM) in LNM. Tumor cells likely used aberrant high-mannose to enhance cell adhesion to the vascular wall, further promoting extravasation and metastasis [36]. Additionally, as we have mentioned earlier, increased complex branched *N*-glycans were observed in tumor tissues and module tumor adhesion through attachment to cadherins and integrins. Increased complex branched *N*-glycans observed in the serum of PTMC with LNM might promote tumor progression and metastasis. What is more, we observed decreased galactosylation in the serum of PTMC with LNM. Theodoratou et al. also found that decreased IgG galactosylation was associated with colorectal cancer prognosis [45]. Several studies reported declined levels of galactosylated *N*-glycans of IgG in ovarian, prostate, gastric, and lung cancers, which were associated with poor prognosis [46,47,48]. Previous studies also suggested possible mechanisms for the decreased IgG galactosylation in cancers, such as the host-defense response to the presence of the tumor [49]. We found decreased serum galactosylation in PTMC with LNM patients compared with that without LNM in the present study. However, the potential role of decreased serum galactosylation in PTMC progression or metastasis deserves further in-depth exploration. Our results also indicated that PTMC with LNM showed higher sialylation than NLNM. Previous studies demonstrated the increase of sialylation could be closely associated with tumor cell adhesion and cellular recognition, thereby supporting metastasis formation [50,51]. Additionally, aberrant sialylation is directly involved in the activation and modulation of the immune system, and accelerates tumor progression and metastasis. Shah et al. assessed serum total sialylation via lectins in the serum of oral cancer and controls, and observed that the sialylation changes in serum were related to neoplastic transformation and progression [52]. We hypothesize that the increased sialylation found in PTMC with LNM compared to PTMC without LNM in the present study may be implicated in the tumor progression and metastasis. It is noteworthy that CA4 and A2F0S0G show robust associations with LNM. We established a predictive model visualized as a nomogram based on CA4 and A2F0S0G for preoperatively identifying LNM to achieve precision therapy. Our nomogram performed better in predicting LNM than ultrasound, and its predictive value was supported by calibration and C-index. Therefore, surgeons can make clinical decisions with the assistance of the nomogram to risk-stratify patients.

The affiliation between PTMC and PTC remains controversial. Studies comparing PTMC and PTC (diameter > 10 mm) patients are fewer and less conclusive at present. In this study, the serum *N*-glycomic profiles of PTMC and PTC (diameter > 10 mm) were compared. To our knowledge, it is the first report to investigate the difference between PTMC and PTC (diameter > 10 mm) at the level of serum *N*-glycome. Here, the findings indicated that PTMC displayed distinct serum *N*-glycome patterns (PTMC-specific) compared to PTC (diameter > 10 mm). Therefore, our *N*-glycomic data indicate that the pathological mechanism of PTMC may not be the same as that of PTC, and PTMC is not the “early stage” of PTC. Further studies will combine clinical data with multi-omics approaches to further explore the underlying mechanisms between PTMC and PTC patients.

Several studies demonstrated that CI was independently associated with an increased risk of LNM [20,53]. Conversely, some scholars have proposed that CI is not a critical factor for triggering LNM. In this study, we explored the correlation between CI and LNM by investigating the CI-related glycan traits and LNM-related glycan traits. We found CI-related glycan traits were different from the LNM-related glycans in PTMC, and there are even opposite changes in some glycan traits, i.e., serum galactosylation levels are elevated in PTMC with the CI group (compared with NCI), and decreased in PTMC with the LNM group (compared with NLNM). These serum *N*-glycomic results suggest that CI may not be related to LNM, at least at the glycan level. The in-depth mechanisms between CI and LNM still need further investigation.

Though we discovered valuable glycan biomarkers and established nomograms for PTMC diagnosis and stratification, the present study has several limitations. First, the methodology of the study cannot provide detailed information on protein origins of *N*-glycan traits, which remains the known challenge in this field, and might be resolved by glycoproteomic analysis. Second, we only explore the association between CI and LNM at serum *N*-glycome levels, and the underlying mechanisms need to combine multi-omic approaches with clinical datasets to further investigate. Third, we only focused on the association between CI and LNM. Due to the sample size of subgroups based on other risk factors not being large enough, we were unable to conduct a stratified analysis. A stratified analysis of subgroups according to age, sex, and multifocality will be performed in future studies. Fourth, an external validation of the logistic models was not conducted. Studies in large validation cohorts are further required to confirm our findings before applications of the glycan-based nomogram.

## 5. Conclusions

In summary, this is the first study focused on PTMC-specific serum *N*-glycomic signatures. The predictive nomograms based on serum *N*-glycome were established for PTMC diagnosis and stratification. We found that CI was not associated with LNM at the level of serum *N*-glycome. Surgeons can give patients preoperative counseling and guidance based on these nomograms. Further protein-specific studies and validation are required to confirm our findings.

## Figures and Tables

**Table 1:** Clinical and pathological characteristics.

Characteristics	PTMC		HC
	with NLNM	with LNM	
Sample size	41	59	80
Age at operation (y, X ± S)	40.34 ± 6.58	38.56 ± 7.91	38.26 ± 6.54
Sex (n (%))			
Female	33 (80.5)	36 (61.1)	40 (50.0)
Male	8 (19.5)	23 (38.9)	40 (50.0)
Family history of thyroid disease (n (%))			
No	31 (75.6)	46 (78.0)	\
Yes	10 (24.4)	13 (22.0)	\
Tumor size (cm, X ± S)	0.69 ± 0.18	0.73 ± 0.23	\
≤0.5	6 (14.6)	15 (25.4)	\
>0.5	35 (85.4)	44 (74.6)	\
Clinical LNM (n (%))			
Absent	41 (100.0)	57 (96.6)	\
Present	0 (0.0)	2 (3.4)	\
Pathological subtype (n (%))			
Classic	32 (78.1)	51 (86.4)	\
Follicular variant	6 (14.6)	5 (8.5)	\
Classic and follicular variant	3 (7.3)	3 (5.1)	\
Tumor location (n (%))			
Unifocal	36 (87.8)	45 (76.3)	\
Multifocal	5 (12.2)	14 (23.7)	\
Tumor calcification (n (%))			
Absent	29 (70.7)	43 (72.9)	\
Present	12 (29.3)	16 (27.1)	\
Microscopic capsular invasion (n (%))			
Absent	14 (34.1)	21 (35.6)	\
Present	27(65.9)	38 (64.4)	\
Hashimoto’s thyroiditis (n (%))			
Absent	30 (73.2)	47 (79.7)	\
Present	11 (26.8)	12 (20.3)	\

Abbreviations: PTMC, papillary thyroid microcarcinoma; HC, healthy controls; LNM, lymph node metastasis; NLNM, non-LNM. Data are presented as mean ± standard deviation or n (%).

**Table 2:** The univariate and multivariate analysis of differentially expressed derived *N*-glycan traits, helping to distinguish between papillary thyroid microcarcinoma (PTMC) and healthy controls (HC) groups.

	Descriptions	Median		*p* Value	
		HC	PTMC	Univariate	Multivariate
**Glycan traits—general**					
TM	High-mannose glycans in total spectrum	0.0227	0.0284	1.38 × 10^−14^	0.002
MHy	The ratio of high-mannose to hybrid glycans	1.7240	2.0062	2.36 × 10^−9^	0.372
CA1	Monoantennary species (A1) in complex glycans	0.0090	0.0096	3.61 × 10^−5^	0.042
CA4	Tetraantennary species (A4) in complex glycans	0.0184	0.0224	1.26 × 10^−8^	0.000
**Glycan traits—fucosylation (F)**					
CFa	Antenna-fucosylation in complex glycans	0.0095	0.0090	7.73 × 10^−5^	0.597
A2Fa	Antenna-fucosylation in diantennary (A2)	0.0094	0.0087	3.94 × 10^−6^	0.010
**Glycan traits—bisection (B)**					
A2F0B	GlcNAc with non-fucosylated diantennary	0.0306	0.0342	0.0002	0.162
A2F0SB	GlcNAc with non-fucosylated sialylated diantennary	0.0222	0.0254	0.0001	0.307

**Table 3:** The univariate and multivariate analysis of differentially expressed derived *N*-glycan traits between papillary thyroid microcarcinoma (PTMC) with lymph node metastasis (LNM) and PTMC with non-LNM (NLNM).

	Descriptions	Median		*p* Value	
		NLNM	LNM	Univariate	Multivariate
**Glycan traits—general**					
MHy	The ratio of high-mannose to hybrid glycans	1.9494	2.0865	0.0265	0.979
MM	Average number of mannoses on high-mannose	6.8579	6.9325	0.0334	0.752
CA2	Diantennary species (A2) in complex glycans	0.8557	0.8413	0.0130	0.853
CA3	Triantennary species (A3) in complex glycans	0.1051	0.1157	0.0485	0.888
CA4	Tetraantennary species (A4) in complex glycans	0.0210	0.0234	0.0006	0.001
**Glycan traits—galactosylation (G)**					
A2F0S0G	In non-fucosylated, non-sialylated diantennary	0.5986	0.5783	0.0073	0.011
**Glycan traits—sialylation (S)**					
A3S	In triantennary (A3)	0.9001	0.9056	0.0358	0.795

**Table 4:** The differentially expressed derived *N*-glycan traits in papillary thyroid microcarcinoma (PTMC) with capsular invasion (CI) compared to PTMC without CI.

	Descriptions	Median of PTMC without CI	Median of PTMC with CI	*p* Value
**Glycan traits—galactosylation (G)**				
CG	In all complex glycans	0.9496	0.9585	0.0045
A2G	In diantennary glycans (A2)	0.8762	0.8919	0.0120
A2FG	In fucosylated diantennary glycans (A2)	0.7334	0.7668	0.0030
A2S0G	In non-sialylated diantennary glycans (A2)	0.5105	0.5447	0.0144
A2FS0G	In fucosylated non-sialylated dianntennary glycans (A2)	0.5066	0.5334	0.0139
**Glycan traits—sialylation (S)**				
CS	In all complex glycans	0.7970	0.8159	0.0377
A2FS	In fucosylated diantennary glycans (A2)	0.3657	0.4026	0.0159
**Glycan traits-α-2,6-linked sialylation (E)**				
A2FE	In fucosylated diantennary glycans (A2)	0.2907	0.3251	0.0147

## Data Availability

Data are available to qualified researchers upon reasonable request of the authors.

## Supplementary Materials

The following supporting information are available online: https://www.mdpi.com/article/10.3390/curroncol29090474/s1, Table S1: The general view of directly detected glycans; Table S2: Description and calculation of derived glycan traits; Table S3: Data quality control; Table S4: Derived *N*-glycan trait changes in the cohort. References [25,26,28,29,30,31,32,33,34] are cited in the supplementary materials.
