# Supplementary material for: Nomograms Based on Serum N-glycome for Diagnosis of Papillary Thyroid Microcarcinoma and Prediction of Lymph Node Metastasis
Source: Curr Oncol. 2022 Aug 23;29(9):6018–34. doi: 10.3390/curroncol29090474 (PMC9497917; doi:10.3390/curroncol29090474)
Supplement: Supplementary file 1 [file curroncol-29-00474-s001.zip › curroncol-1799346-supplementary.pdf]

Current Oncology

Supplementary Material

# Nomograms Based on Serum *N*-glycome for Diagnosis of Papillary Thyroid Microcarcinoma and Prediction of Lymph Node Metastasis

**Zejian Zhang [1,†], Zhen Cao [2,†], Rui Liu[2], Zepeng Li [3], Jianqiang Wu [1], Xiaoli Liu [4], Mengwei Wu [2], Xiequn Xu[2,*] and Ziwen Liu [2,*]**

1 Department of Medical Research Center, State Key Laboratory of Complex Severe and Rare Diseases, Peking Union Medical College Hospital, Chinese Academy of Medical Sciences and Peking Union Medical College, Beijing 100730, China
2 Department of General Surgery, Peking Union Medical College Hospital, Chinese Academy of Medical Sciences and Peking Union Medical College, Beijing 100730, China
3 Department of Clinical Laboratory, Peking Union Medical College Hospital, Chinese Academy of Medical Sciences and Peking Union Medical College, Beijing 100730, China
4 Department of Hernia and Abdominal Wall Surgery, Beijing Chao-Yang Hospital, Capital Medical University, Beijing 100043, China
* Correspondence: xxq75@163.com (X.-Q. X); Tel.: +86-010-69152620; liuziwen@pumch.cn (Z.-W. L.); Tel.: +86-010-69152620.
† These authors equally contributed to this work.

## Detailed Protocols

### Serum *N*-glycome Detection and MS Data Processing

A total of 180 serum samples from the cohort (100 PTMC and 80 HC) and 17 quality control samples (5 blanks and 12 replicated serum standards) were randomly distributed into three 96-well plates. According to the protocol described previously, serum glycoproteins from the samples were enzymatically treated to release *N*-glycans [28]. Briefly, 10 µl of 2% sodium dodecyl sulfonate was added to 5 µl of serum specimen, and then the mixture was incubated for 10 min at 60 ℃. The process of *N*-glycan release was performed by the addition of 10 µl of the mixture (2% Nonidet P-40, 2.5 × phosphate-buffered salines, and 1 U PNGase F), followed by incubation at 37 ℃ overnight. For better discrimination of linkage-specific sialic acids located at the non-reducing ends of glycans, $\alpha$-2,3- linked sialic acids were lactonized and $\alpha$-2,6-linked sialic acids were ethyl-esterified, permitting mass-based distinction among the sialic-acid linkage variants [28]. In short, 1 µl of release mixture was added to 20 µl of derivatization reagent (250 mM 1-ethyl-3-(3-dimethylaminopropyl)-carbodiimide and 250 mM hydroxybenzotriazole in ethanol), followed by incubation for 1 h at 37 ℃. The derivatized *N*-glycans were then purified by hydrophilic interaction liquid chromatography solid-phase extraction (HILIC-SPE) micro-tips using cotton thread as the stationary phase packed in the pipette tips and enriched glycans were eventually eluted with MilliQ water according to the previously reported method [26,29,30]. HILIC-SPE cotton tips allowed the removal of salts, most deglycosylated peptides, and detergents from glycoconjugate samples. Additionally, subsequent MALDI-TOF MS glycan profiles were very repeatable with different tips. The sialylated *N*-glycans were detected simultaneously with non-sialylated *N*-glycans using MALDI-TOF MS in positive-ion mode. Whereafter, 1 µl of purified glycans were mixed with 1 µl of the matrix (5 mg/ml super-2,5-dihydroxybenzoic acid in 50% acetonitrile with 1 mM NaOH) on a target plate and were air-dried at room temperature. The state-of-the-art and high-resolution MS platform（rapifleXtreme MALDI-TOF mass spectrometer) was employed to measure the purified glycans. The instrument was equipped with a Smartbeam-3D laser and was controlled by flexControl 4.0 software (Bruker Daltonics). The equipment was calibrated by external calibrants (Bruker Peptide Calibration Standard II). The mass range of measurements was set to 1,000–5,000 m/z. For each spot, 5,000 laser shots were accumulated in random walking mode at a laser frequency of 5,000 Hz.

The raw MS data were processed according to the workflow reported in the previous study [28–30]. The raw MS data had been deposited in GlycoPOST (ID: GPST000252) [31]. Briefly, the raw data were smoothed with Savitzky Golay algorithm by flexAnalysis software, baseline corrected with the TopHat method, and transformed to.XY files using the software of flexAnalysis (Bruker Daltonics). Transformed .XY files were then re-calibrated using internally developed MassyTools software (version 0.1.8.1.2) using a selection of well-known high-intensity glycan signals distributed across the detected m/z rang (minimum five calibrants at S/N > 9) [32]. The peaks of MS spectra were manually assigned to *N*-glycan compositions using the GlycoPeakfinder tool of GlycoWorkbench [33,34]. It used data from single MS measurements to estimate the quantities and classed of monosaccharide components of the glycan structures. It generates only the *N*-linked glycans possibly synthesized by mammalian cells using a set of archetypal structures and a set of rules for the modification of said structures. The *N*-glycan compositions were also confirmed by previous literature [25,28,29]. Briefly, peaks (isotope clusters) with a signal-to-noise ratio (S/N) above nine and good isotopic patterns were listed. Next, the peaks of MS spectra (analytes) were manually assigned to *N*-glycan structures. These peaks (analytes) were annotated with glycan compositions in GlycoWorkbench (version 2.1 stable build 146) using the GlycoPeakfinder tool. The generated glycan compositions were then confirmed and validated by previous literature. Finally, the composition list of 131 putative *N*-glycan compositions was generated for subsequent targeted data extraction. The peak intensities of putative glycan composition were extracted as peak areas (background-corrected) by the composition list and MassyTools. The extracted data were further processed in Microsoft Excel. In the furthering process, 35 out of 131 glycan compositions were excluded after applying the cut-offs of S/N > 9, ppm error < 20, QC score < 25%, and the minimum percentage (>50%) of presence in all spectra of PTMC, HC, or quality control serum samples [29]. After the curation, 96/131 *N*-glycan compositions met the

quality criteria and were included in quantitative analysis (Supplementary Table S1). At last, the sum of glycan areas per spectrum was re-scaled to 1 to assess relative intensities. To explore the exact role of a group of *N*-glycans with similar glycan structures and to explain their biological effects, such as the number of antennae of complex type *N*-glycans (CA), the level of bisection (B), fucosylation (F), galactosylation (G) and sialylation (S), 91 derived *N*-glycan traits were calculated from the 96 directly detected *N*-glycan traits based on their common structural characteristics. The formulas used for the calculation of derived glycan traits are given in Supplementary Table S2. The calculations were implemented in RStudio. Aberrant derived glycan traits indicate that changes in glycosylation are shared by a series of structurally related *N*-glycans [29].

The average value, standard deviation (SD), and coefficient of variance (CV) of the 12 replicated standard samples were calculated for directly detected and derived glycan traits to evaluate the quality of the data (Supplementary Table S3).

## References


**[25]** Zhang, Z., K. R. Reiding, J. Wu, Z. Li, and X. Xu. "Distinguishing Benign and Malignant Thyroid Nodules and Identifying Lymph Node Metastasis in Papillary Thyroid Cancer by Plasma N-Glycomics." *Front. Endocrinol.* **2021**, *12*, 692910.

**[26]** Vreeker, G. C. M., R. G. Hanna-Sawires, Y. Mohammed, M. R. Bladergroen, S. Nicolardi, V. Dotz, J. Nouta, B. A. Bonsing, W. E. Mesker, Y. E. M. van der Burgt, M. Wuhrer, and Raem Tollenaar. "Serum N-Glycome Analysis Reveals Pancreatic Cancer Disease Signatures. *Cancer Med.* **2020**, *9*, 8519–8529.

**[28]** Reiding, K. R., D. Blank, D. M. Kuijper, A. M. Deelder, and M. Wuhrer. "High-Throughput Profiling of Protein N-Glycosylation by Maldi-Tof-Ms Employing Linkage-Specific Sialic Acid Esterification. *Anal. Chem.* **2014**, *86*, 5784-93.

**[29]** Zhang, Z., M. Westhrin, A. Bondt, M. Wuhrer, T. Standal, and S. Holst. "Serum Protein N-Glycosylation Changes in Multiple Myeloma. *Biochim. Biophys. Acta Gen. Subj.* **2019**, *1863*, 960–970.

**[30]** Zhang, Z., J. Wu, P. Liu, L. Kang, and X. Xu. "Diagnostic Potential of Plasma Igg N-Glycans in Discriminating Thyroid Cancer from Benign Thyroid Nodules and Healthy Controls. *Front. Oncol.* **2021,** *11*. 658223.

**[31]** Watanabe, Y., K. F. Aoki-Kinoshita, Y. Ishihama, and S. Okuda. "Glycopost Realizes Fair Principles for Glycomics Mass Spectrometry Data. *Nucleic Acids Res.* **2021,** *49*, D1523–D1528.

**[32]** Jansen, B. C., K. R. Reiding, A. Bondt, A. L. Hipgrave Ederveen, M. Palmblad, D. Falck, and M. Wuhrer. "Massytools: A High-Throughput Targeted Data Processing Tool for Relative Quantitation and Quality Control Developed for Glycomic and Glycoproteomic Maldi-Ms. *J. Proteome Res.* **2015,** *14*, 5088-5098.

**[33]** Maass, K., R. Ranzinger, H. Geyer, C. W. von der Lieth, and R. Geyer. ""Glyco-Peakfinder"--De Novo Composition Analysis of Glycoconjugates. *Proteomics* **2007**, *7*, 4435–4444.

**[34]** Ceroni, A., K. Maass, H. Geyer, R. Geyer, A. Dell, and S. M. Haslam. "Glycoworkbench: A Tool for the Computer-Assisted Annotation of Mass Spectra of Glycans. *J. Proteome Res.* **2008**, *7*, 1650–1659.

## Supplemental Tables

**Table S1.** The general view of directly detected glycans. Compositions were detected by MALDI-TOF-MS after ethyl esterification. All species are assigned [M+Na]+. H = hexose; N = N-acetylhexosamine; F = deoxyhexose (fucose); L = lactonized N-acetylneuraminic acid (α2,3-linked); E = ethyl esterified N-acetylneuraminic acid (α2,6-linked).

| Observed m/z | Input composition | | | | | Modifiers composition | | | Calculated | | Error | |
|---|---|---|---|---|---|---|---|---|---|---|---|---|
| | H | N | F | L | E | H2O | Na+ | Ac | Traits | Mass | error (Da) | error (ppm) |
| | 162.053 | 203.079 | 146.058 | 273.085 | 319.127 | 18.0106 | 22.9892 | 42.0106 | | | | |
| 1136.389 | 3 | 3 | 0 | 0 | 0 | 1 | 1 | 0 | H3N3 | 1136.396 | -0.00701832 | -6.175948037 |
| 1257.423 | 5 | 2 | 0 | 0 | 0 | 1 | 1 | 0 | H5N2 | 1257.423 | 0.000575309 | 0.457530569 |
| 1282.461 | 3 | 3 | 1 | 0 | 0 | 1 | 1 | 0 | H3N3F1 | 1282.454 | 0.006860206 | 5.349279562 |
| 1298.443 | 4 | 3 | 0 | 0 | 0 | 1 | 1 | 0 | H4N3 | 1298.449 | -0.00615251 | -4.738353335 |
| 1339.473 | 3 | 4 | 0 | 0 | 0 | 1 | 1 | 0 | H3N4 | 1339.476 | -0.00311986 | -2.329165751 |
| 1419.473 | 6 | 2 | 0 | 0 | 0 | 1 | 1 | 0 | H6N2 | 1419.475 | -0.00234941 | -1.655124938 |
| 1444.503 | 4 | 3 | 1 | 0 | 0 | 1 | 1 | 0 | H4N3F1 | 1444.507 | -0.00364159 | -2.520993062 |
| 1455.526 | 3 | 3 | 0 | 0 | 1 | 1 | 1 | 0 | H3N3E1 | 1455.523 | 0.003056084 | 2.099646409 |
| 1460.493 | 5 | 3 | 0 | 0 | 0 | 1 | 1 | 0 | H5N3 | 1460.502 | -0.00902302 | -6.178025033 |
| 1485.531 | 3 | 4 | 1 | 0 | 0 | 1 | 1 | 0 | H3N4F1 | 1485.534 | -0.00246156 | -1.657020892 |
| 1501.523 | 4 | 4 | 0 | 0 | 0 | 1 | 1 | 0 | H4N4 | 1501.529 | -0.0059364 | -3.953570223 |
| 1542.557 | 3 | 5 | 0 | 0 | 0 | 1 | 1 | 0 | H3N5 | 1542.555 | 0.001910484 | 1.238519037 |
| 1581.53 | 7 | 2 | 0 | 0 | 0 | 1 | 1 | 0 | H7N2 | 1581.528 | 0.001989074 | 1.257691327 |
| 1601.583 | 3 | 3 | 1 | 0 | 1 | 1 | 1 | 0 | H3N3F1E1 | 1601.581 | 0.00230257 | 1.437685582 |
| 1606.564 | 5 | 3 | 1 | 0 | 0 | 1 | 1 | 0 | H5N3F1 | 1606.56 | 0.004119532 | 2.564194616 |
| 1617.57 | 4 | 3 | 0 | 0 | 1 | 1 | 1 | 0 | H4N3E1 | 1617.576 | -0.00543612 | -3.360657504 |
| 1622.556 | 6 | 3 | 0 | 0 | 0 | 1 | 1 | 0 | H6N3 | 1622.555 | 0.001369247 | 0.843883068 |
| 1631.582 | 3 | 4 | 2 | 0 | 0 | 1 | 1 | 0 | H3N4F2 | 1631.592 | -0.00983831 | -6.029883037 |
| 1647.582 | 4 | 4 | 1 | 0 | 0 | 1 | 1 | 0 | H4N4F1 | 1647.586 | -0.00464598 | -2.819867875 |
| 1663.582 | 5 | 4 | 0 | 0 | 0 | 1 | 1 | 0 | H5N4 | 1663.581 | 0.000509927 | 0.306523547 |
| 1688.605 | 3 | 5 | 1 | 0 | 0 | 1 | 1 | 0 | H3N5F1 | 1688.613 | -0.00765126 | -4.531091326 |
| 1701.594 | 3 | 3 | 2 | 1 | 0 | 1 | 1 | 0 | H3N3F2L1 | 1701.597 | -0.00349439 | -2.053594502 |
| 1704.605 | 4 | 5 | 0 | 0 | 0 | 1 | 1 | 0 | H4N5 | 1704.608 | -0.00258875 | -1.518680318 |
| 1717.594 | 4 | 3 | 1 | 1 | 0 | 1 | 1 | 0 | H4N3F1L1 | 1717.592 | 0.002538478 | 1.477928421 |
| 1733.591 | 5 | 3 | 0 | 1 | 0 | 1 | 1 | 0 | H5N3L1 | 1733.587 | 0.004535191 | 2.616073918 |
| 1743.583 | 8 | 2 | 0 | 0 | 0 | 1 | 1 | 0 | H8N2 | 1743.581 | 0.001590847 | 0.912401903 |
| 1763.63 | 4 | 3 | 1 | 0 | 1 | 1 | 1 | 0 | H4N3F1E1 | 1763.634 | -0.00355768 | -2.01724669 |
| 1774.617 | 4 | 4 | 0 | 1 | 0 | 1 | 1 | 0 | H4N4L1 | 1774.613 | 0.004027614 | 2.269572427 |
| 1779.638 | 5 | 3 | 0 | 0 | 1 | 1 | 1 | 0 | H5N3E1 | 1779.629 | 0.009333405 | 5.244580056 |
| 1804.662 | 3 | 4 | 1 | 0 | 1 | 1 | 1 | 0 | H3N4F1E1 | 1804.66 | 0.0017589 | 0.974643256 |
| 1809.644 | 5 | 4 | 1 | 0 | 0 | 1 | 1 | 0 | H5N4F1 | 1809.639 | 0.004431856 | 2.449027509 |

| | | | | | | | | | | | | |
|---|---|---|---|---|---|---|---|---|---|---|---|---|
| 1820.65 | 4 | 4 | 0 | 0 | 1 | 1 | 1 | 0 | H4N4E1 | 1820.655 | -0.00544184 | -2.988947712 |
| 1850.668 | 4 | 5 | 1 | 0 | 0 | 1 | 1 | 0 | H4N5F1 | 1850.666 | 0.002480382 | 1.340264678 |
| 1866.652 | 5 | 5 | 0 | 0 | 0 | 1 | 1 | 0 | H5N5 | 1866.661 | -0.00882467 | -4.727519116 |
| 1879.642 | 5 | 3 | 1 | 1 | 0 | 1 | 1 | 0 | H5N3F1L1 | 1879.645 | -0.00299704 | -1.594472871 |
| 1895.645 | 6 | 3 | 0 | 1 | 0 | 1 | 1 | 0 | H6N3L1 | 1895.64 | 0.005630157 | 2.9700563 |
| 1905.626 | 9 | 2 | 0 | 0 | 0 | 1 | 1 | 0 | H9N2 | 1905.634 | -0.00752287 | -3.947699528 |
| 1936.659 | 5 | 4 | 0 | 1 | 0 | 1 | 1 | 0 | H5N4L1 | 1936.666 | -0.0068224 | -3.522752224 |
| 1941.672 | 6 | 3 | 0 | 0 | 1 | 1 | 1 | 0 | H6N3E1 | 1941.682 | -0.00963158 | -4.960432549 |
| 1966.702 | 4 | 4 | 1 | 0 | 1 | 1 | 1 | 0 | H4N4F1E1 | 1966.713 | -0.01078379 | -5.483152328 |
| 1982.709 | 5 | 4 | 0 | 0 | 1 | 1 | 1 | 0 | H5N4E1 | 1982.708 | 0.000644831 | 0.325227322 |
| 2012.72 | 5 | 5 | 1 | 0 | 0 | 1 | 1 | 0 | H5N5F1 | 2012.719 | 0.00087556 | 0.435013532 |
| 2023.723 | 4 | 5 | 0 | 0 | 1 | 1 | 1 | 0 | H4N5E1 | 2023.735 | -0.01131597 | -5.591627877 |
| 2082.724 | 5 | 4 | 1 | 1 | 0 | 1 | 1 | 0 | H5N4F1L1 | 2082.724 | -0.00057709 | -0.277082378 |
| 2093.742 | 4 | 4 | 0 | 1 | 1 | 1 | 1 | 0 | H4N4L1E1 | 2093.74 | 0.002119956 | 1.01252091 |
| 2128.767 | 5 | 4 | 1 | 0 | 1 | 1 | 1 | 0 | H5N4F1E1 | 2128.766 | 0.000933599 | 0.438563553 |
| 2169.786 | 4 | 5 | 1 | 0 | 1 | 1 | 1 | 0 | H4N5F1E1 | 2169.793 | -0.00688035 | -3.170972408 |
| 2177.758 | 3 | 4 | 2 | 2 | 0 | 1 | 1 | 0 | H3N4F2L2 | 2177.761 | -0.00363124 | -1.667416974 |
| 2185.791 | 5 | 5 | 0 | 0 | 1 | 1 | 1 | 0 | H5N5E1 | 2185.787 | 0.003085815 | 1.411763319 |
| 2255.783 | 5 | 4 | 0 | 1 | 1 | 1 | 1 | 0 | H5N4L1E1 | 2255.793 | -0.01008956 | -4.472735009 |
| 2280.812 | 3 | 5 | 1 | 1 | 1 | 1 | 1 | 0 | H3N5F1L1E1 | 2280.825 | -0.01236064 | -5.419372943 |
| 2285.838 | 4 | 4 | 1 | 0 | 2 | 1 | 1 | 0 | H4N4F1E2 | 2285.84 | -0.00141738 | -0.620070567 |
| 2301.852 | 5 | 4 | 0 | 0 | 2 | 1 | 1 | 0 | H5N4E2 | 2301.835 | 0.017283099 | 7.508400844 |
| 2331.843 | 5 | 5 | 1 | 0 | 1 | 1 | 1 | 0 | H5N5F1E1 | 2331.845 | -0.00284624 | -1.220594544 |
| 2339.806 | 4 | 4 | 2 | 2 | 0 | 1 | 1 | 0 | H4N4F2L2 | 2339.814 | -0.00777974 | -3.324937397 |
| 2347.854 | 6 | 5 | 0 | 0 | 1 | 1 | 1 | 0 | H6N5E1 | 2347.84 | 0.013749711 | 5.856323136 |
| 2355.822 | 5 | 4 | 1 | 2 | 0 | 1 | 1 | 0 | H5N4F1L2 | 2355.809 | 0.012797436 | 5.432289087 |
| 2372.874 | 4 | 6 | 1 | 0 | 1 | 1 | 1 | 0 | H4N6F1E1 | 2372.872 | 0.00215211 | 0.906964111 |
| 2385.833 | 4 | 4 | 2 | 1 | 1 | 1 | 1 | 0 | H4N4F2L1E1 | 2385.856 | -0.02295117 | -9.619677705 |
| 2401.84 | 5 | 4 | 1 | 1 | 1 | 1 | 1 | 0 | H5N4F1L1E1 | 2401.851 | -0.01047759 | -4.362298422 |
| 2429.894 | 4 | 7 | 0 | 0 | 1 | 1 | 1 | 0 | H4N7E1 | 2429.893 | 0.000323666 | 0.133201765 |
| 2447.902 | 5 | 4 | 1 | 0 | 2 | 1 | 1 | 0 | H5N4F1E2 | 2447.893 | 0.008938643 | 3.651566502 |
| 2493.898 | 6 | 5 | 1 | 0 | 1 | 1 | 1 | 0 | H6N5F1E1 | 2493.898 | -0.00055004 | -0.22055587 |
| 2504.924 | 5 | 5 | 0 | 0 | 2 | 1 | 1 | 0 | H5N5E2 | 2504.914 | 0.009500801 | 3.792864872 |
| 2542.892 | 4 | 5 | 2 | 2 | 0 | 1 | 1 | 0 | H4N5F2L2 | 2542.893 | -0.00166172 | -0.653477145 |
| 2620.942 | 6 | 5 | 0 | 1 | 1 | 1 | 1 | 0 | H6N5L1E1 | 2620.925 | 0.016819697 | 6.417465758 |
| 2650.964 | 5 | 5 | 1 | 0 | 2 | 1 | 1 | 0 | H5N5F1E2 | 2650.972 | -0.00806371 | -3.041792284 |
| 2666.971 | 6 | 5 | 0 | 0 | 2 | 1 | 1 | 0 | H6N5E2 | 2666.967 | 0.003805836 | 1.427027717 |
| 2704.947 | 5 | 5 | 2 | 2 | 0 | 1 | 1 | 0 | H5N5F2L2 | 2704.946 | 0.000765752 | 0.283093381 |
| 2712.975 | 7 | 6 | 0 | 0 | 1 | 1 | 1 | 0 | H7N6E1 | 2712.973 | 0.002880936 | 1.061911147 |
| 2720.941 | 6 | 5 | 1 | 2 | 0 | 1 | 1 | 0 | H6N5F1L2 | 2720.941 | -0.00048497 | -0.178235572 |
| 2758.997 | 8 | 7 | 0 | 0 | 0 | 1 | 1 | 0 | H8N7 | 2758.978 | 0.018651141 | 6.760162974 |

| | | | | | | | | | | | | |
|---|---|---|---|---|---|---|---|---|---|---|---|---|
| 2766.979 | 6 | 5 | 1 | 1 | 1 | 1 | 1 | 0 | H6N5F1L1E1 | 2766.983 | -0.00415612 | -1.502041968 |
| 2813.022 | 6 | 5 | 1 | 0 | 2 | 1 | 1 | 0 | H6N5F1E2 | 2813.025 | -0.00313008 | -1.112709047 |
| 2894.01 | 6 | 5 | 0 | 2 | 1 | 1 | 1 | 0 | H6N5L2E1 | 2894.01 | 0.000277577 | 0.095914226 |
| 2940.055 | 6 | 5 | 0 | 1 | 2 | 1 | 1 | 0 | H6N5L1E2 | 2940.052 | 0.003470376 | 1.180379336 |
| 2986.097 | 6 | 5 | 0 | 0 | 3 | 1 | 1 | 0 | H6N5E3 | 2986.094 | 0.00336213 | 1.125929205 |
| 2994.008 | 6 | 5 | 1 | 3 | 0 | 1 | 1 | 0 | H6N5F1L3 | 2994.026 | -0.0179145 | -5.983414955 |
| 3032.102 | 7 | 6 | 0 | 0 | 2 | 1 | 1 | 0 | H7N6E2 | 3032.099 | 0.00234704 | 0.774064478 |
| 3040.073 | 6 | 5 | 1 | 2 | 1 | 1 | 1 | 0 | H6N5F1L2E1 | 3040.068 | 0.00461779 | 1.518975771 |
| 3086.117 | 6 | 5 | 1 | 1 | 2 | 1 | 1 | 0 | H6N5F1L1E2 | 3086.11 | 0.006853262 | 2.220679863 |
| 3124.121 | 9 | 8 | 0 | 0 | 0 | 1 | 1 | 0 | H9N8 | 3124.11 | 0.01041013 | 3.332190394 |
| 3132.158 | 6 | 5 | 1 | 0 | 3 | 1 | 1 | 0 | H6N5F1E3 | 3132.152 | 0.006787482 | 2.167035005 |
| 3232.178 | 6 | 5 | 2 | 1 | 2 | 1 | 1 | 0 | H6N5F2L1E2 | 3232.168 | 0.010198483 | 3.155307634 |
| 3259.15 | 7 | 6 | 0 | 2 | 1 | 1 | 1 | 0 | H7N6L2E1 | 3259.142 | 0.008013 | 2.458622345 |
| 3305.166 | 7 | 6 | 0 | 1 | 2 | 1 | 1 | 0 | H7N6L1E2 | 3305.184 | -0.01817955 | -5.500313056 |
| 3351.227 | 7 | 6 | 0 | 0 | 3 | 1 | 1 | 0 | H7N6E3 | 3351.226 | 0.0013127 | 0.391707409 |
| 3397.243 | 8 | 7 | 0 | 0 | 2 | 1 | 1 | 0 | H8N7E2 | 3397.231 | 0.011902185 | 3.503495644 |
| 3405.206 | 7 | 6 | 1 | 2 | 1 | 1 | 1 | 0 | H7N6F1L2E1 | 3405.2 | 0.005761072 | 1.691845403 |
| 3443.237 | 9 | 8 | 0 | 0 | 1 | 1 | 1 | 0 | H9N8E1 | 3443.237 | 0.000197098 | 0.057241975 |
| 3532.239 | 7 | 6 | 0 | 3 | 1 | 1 | 1 | 0 | H7N6L3E1 | 3532.227 | 0.011649966 | 3.298192883 |
| 3578.275 | 7 | 6 | 0 | 2 | 2 | 1 | 1 | 0 | H7N6L2E2 | 3578.269 | 0.005661174 | 1.582098505 |
| 3624.32 | 7 | 6 | 0 | 1 | 3 | 1 | 1 | 0 | H7N6L1E3 | 3624.311 | 0.009367276 | 2.584567647 |
| 3678.297 | 7 | 6 | 1 | 3 | 1 | 1 | 1 | 0 | H7N6F1L3E1 | 3678.285 | 0.011717035 | 3.185461475 |
| 3724.336 | 7 | 6 | 1 | 2 | 2 | 1 | 1 | 0 | H7N6F1L2E2 | 3724.327 | 0.00876955 | 2.354667257 |
| 4081.498 | 9 | 8 | 0 | 0 | 3 | 1 | 1 | 0 | H9N8E3 | 4081.49 | 0.007251185 | 1.7766023 |

**Table S2. Description and calculation of derived glycan traits.** Within the depictions, divisions have been indicated by braces, and exclusions by a cross, whereas numerical values indicate compositional limitations (i.e. 5-9 as the possible number of mannoses). M = mannose; Hy = hybrid species; T = within total spectrum; C = within complex species; F = deoxy-hexose (fucose); G = galactose; S = N-acetylneuraminic acid (sialic acid); E = $\alpha$2,6-linked sialic acid; L = $\alpha$2,3-linked sialic acid; H = hexose (mannose or galactose); N = N-acetylhexosamine (N-acetylglucosamine: GlcNAc).

| Derived trait | Cartoon illustration | Description | Formular of calculation |
|---|---|---|---|
| | | **Glycan type** | |
| **TM** | 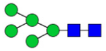 | Relative abundance of high mannose type glycans within total spectrum | TM = ( H5N2 + H6N2 + H7N2 + H8N2 + H9N2 ) / ( H3N3 + H5N2 + H3N3F1 + H4N3 + H3N4 + H6N2 + H4N3F1 + H3N3E1 + H5N3 + H3N4F1 + H4N4 + H3N5 + H7N2 + H3N3F1E1 + H5N3F1 + H4N3E1 + H6N3 + H3N4F2 + H4N4F1 + H5N4 + H3N5F1 + H3N3F2L1 + H4N5 + H4N3F1L1 + H5N3L1 + H8N2 + H4N3F1E1 + H4N4L1 + H5N3E1 + H3N4F1E1 + H5N4F1 + H4N4E1 + H4N5F1 + H5N5 + H5N3F1L1 + H6N3L1 + H9N2 + H5N4L1 + H6N3E1 + H4N4F1E1 + H5N4E1 + H5N5F1 + H4N5E1 + H5N4F1L1 + H4N4E1L1 + H5N4F1E1 + H4N5F1E1 + H3N4F2L2 + H5N5E1 + H5N4E1L1 + H3N5F1E1L1 + H4N4F1E2 + H5N4E2 + H5N5F1E1 + H4N4F2L2 + H6N5E1 + H5N4F1L2 + H4N6F1E1 + H4N4F2E1L1 + H5N4F1E1L1 + H4N7E1 + H5N4F1E2 + H6N5F1E1 + H5N5E2 + H4N5F2L2 + H6N5E1L1 + H5N5F1E2 + H6N5E2 + H5N5F2L2 + H7N6E1 + H6N5F1L2 + H8N7 + H6N5F1E1L1 + H6N5F1E2 + H6N5E1L2 + H6N5E2L1 + H6N5E3 + H6N5F1L3 + H7N6E2 + H6N5F1E1L2 + H6N5F1E2L1 + H9N8 + H6N5F1E3 + H6N5F2E2L1 + H7N6E1L2 + H7N6E2L1 + H7N6E3 + H8N7E2 + H7N6F1E1L2 + H9N8E1 + H7N6E1L3 + H7N6E2L2 + H7N6E3L1 + H7N6F1E1L3 + H7N6F1E2L2 + H9N8E3 ) |
| **THy** | 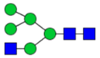 | Relative abundance of hybrid type glycans within total spectrum | THy = ( H5N3 + H5N3F1 + H6N3 + H5N3L1 + H5N3E1 + H5N3F1L1 + H6N3L1 + H6N3E1 ) / ( H3N3 + H5N2 + H3N3F1 + H4N3 + H3N4 + H6N2 + H4N3F1 + H3N3E1 + H5N3 + H3N4F1 + H4N4 + H3N5 + H7N2 + H3N3F1E1 + H5N3F1 + H4N3E1 + H6N3 + H3N4F2 + H4N4F1 + H5N4 + H3N5F1 + H3N3F2L1 + H4N5 + H4N3F1L1 + H5N3L1 + H8N2 + H4N3F1E1 + H4N4L1 + H5N3E1 + H3N4F1E1 + H5N4F1 + H4N4E1 + H4N5F1 + H5N5 + H5N3F1L1 + H6N3L1 + H9N2 + H5N4L1 + H6N3E1 + H4N4F1E1 + H5N4E1 + H5N5F1 + H4N5E1 + H5N4F1L1 + H4N4E1L1 + H5N4F1E1 + H4N5F1E1 + H3N4F2L2 + H5N5E1 + H5N4E1L1 + H3N5F1E1L1 + H4N4F1E2 + H5N4E2 + H5N5F1E1 + H4N4F2L2 + H6N5E1 + H5N4F1L2 + H4N6F1E1 + H4N4F2E1L1 + H5N4F1E1L1 + H4N7E1 + H5N4F1E2 + H6N5F1E1 + H5N5E2 + H4N5F2L2 + H6N5E1L1 + H5N5F1E2 + H6N5E2 + H5N5F2L2 + H7N6E1 + H6N5F1L2 + H8N7 + H6N5F1E1L1 + H6N5F1E2 + H6N5E1L2 + H6N5E2L1 + H6N5E3 + H6N5F1L3 + H7N6E2 + H6N5F1E1L2 + H6N5F1E2L1 + H9N8 + H6N5F1E3 + H6N5F2E2L1 + H7N6E1L2 + H7N6E2L1 + H7N6E3 + H8N7E2 + H7N6F1E1L2 + H9N8E1 + H7N6E1L3 + H7N6E2L2 + H7N6E3L1 + H7N6F1E1L3 + H7N6F1E2L2 + H9N8E3 ) |
| **MHy** | 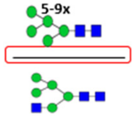 | The ratio of high-mannose to hybrid glycans | MHy = ( H5N2 + H6N2 + H7N2 + H8N2 + H9N2 ) / ( H5N3 + H5N3F1 + H6N3 + H5N3L1 + H5N3E1 + H5N3F1L1 + H6N3L1 + H6N3E1 ) |
| **MM** | 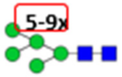 | Average number of mannoses on high mannose type glycans | MM = ( 5 * ( H5N2 ) + 6 * ( H6N2 ) + 7 * ( H7N2 ) + 8 * ( H8N2 ) + 9 * ( H9N2 ) + 10 * (0) ) / ( H5N2 + H6N2 + H7N2 + H8N2 + H9N2 ) |
| **CA1** | 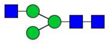 | Relative abundance of monoantennary glycans within complex type glycans | CA1 = ( H3N3E1 + H3N3F1E1 + H4N3E1 + H3N3F2L1 + H4N3F1L1 + H4N3F1E1 ) / ( H3N4 + H3N3E1 + H3N4F1 + H4N4 + H3N5 + H3N3F1E1 + H4N3E1 + H3N4F2 + H4N4F1 + H5N4 + H3N5F1 + H3N3F2L1 + H4N5 + H4N3F1L1 + H4N3F1E1 + H4N4L1 + H3N4F1E1 + H5N4F1 + H4N4E1 + H4N5F1 + H5N5 + H5N4L1 + H4N4F1E1 + H5N4E1 + H5N5F1 + H4N5E1 + H5N4F1L1 + H4N4E1L1 + H5N4F1E1 + H4N5F1E1 + H3N4F2L2 + H5N5E1 + H5N4E1L1 + H3N5F1E1L1 + H4N4F1E2 + H5N4E2 + H5N5F1E1 + H4N4F2L2 + H6N5E1 + H5N4F1L2 + H4N6F1E1 + H4N4F2E1L1 + H5N4F1E1L1 + H4N7E1 + H5N4F1E2 + H6N5F1E1 + H5N5E2 + H4N5F2L2 + H6N5E1L1 + H5N5F1E2 + H6N5E2 + H5N5F2L2 + H7N6E1 + |

| | | | |
|---|---|---|---|
| | | | H6N5F1L2 + H8N7 + H6N5F1E1L1 + H6N5F1E2 + H6N5E1L2 + H6N5E2L1 + H6N5E3 + H6N5F1L3 + H7N6E2 + H6N5F1E1L2 + H6N5F1E2L1 + H9N8 + H6N5F1E3 + H6N5F2E2L1 + H7N6E1L2 + H7N6E2L1 + H7N6E3 + H8N7E2 + H7N6F1E1L2 + H9N8E1 + H7N6E1L3 + H7N6E2L2 + H7N6E3L1 + H7N6F1E1L3 + H7N6F1E2L2 + H9N8E3 ) |
| **CA2** | 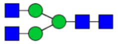 | Relative abundance of diantennary glycans within complex type glycans | CA2 = ( H3N4 + H3N4F1 + H4N4 + H3N5 + H3N4F2 + H4N4F1 + H5N4 + H3N5F1 + H4N5 + H4N4L1 + H3N4F1E1 + H5N4F1 + H4N4E1 + H4N5F1 + H5N5 + H5N4L1 + H4N4F1E1 + H5N4E1 + H5N5F1 + H4N5E1 + H5N4F1L1 + H4N4E1L1 + H5N4F1E1 + H4N5F1E1 + H3N4F2L2 + H5N5E1 + H5N4E1L1 + H3N5F1E1L1 + H4N4F1E2 + H5N4E2 + H5N5F1E1 + H4N4F2L2 + H5N4F1L2 + H4N4F2E1L1 + H5N4F1E1L1 + H5N4F1E2 + H5N5E2 + H4N5F2L2 + H5N5F1E2 + H5N5F2L2 ) / ( H3N4 + H3N3E1 + H3N4F1 + H4N4 + H3N5 + H3N3F1E1 + H4N3E1 + H3N4F2 + H4N4F1 + H5N4 + H3N5F1 + H3N3F2L1 + H4N5 + H4N3F1L1 + H4N3F1E1 + H4N4L1 + H3N4F1E1 + H5N4F1 + H4N4E1 + H4N5F1 + H5N5 + H5N4L1 + H4N4F1E1 + H5N4E1 + H5N5F1 + H4N5E1 + H5N4F1L1 + H4N4E1L1 + H5N4F1E1 + H4N5F1E1 + H3N4F2L2 + H5N5E1 + H5N4E1L1 + H3N5F1E1L1 + H4N4F1E2 + H5N4E2 + H5N5F1E1 + H4N4F2L2 + H6N5E1 + H5N4F1L2 + H4N6F1E1 + H4N4F2E1L1 + H5N4F1E1L1 + H4N7E1 + H5N4F1E2 + H6N5F1E1 + H5N5E2 + H4N5F2L2 + H6N5E1L1 + H5N5F1E2 + H6N5E2 + H5N5F2L2 + H7N6E1 + H6N5F1L2 + H8N7 + H6N5F1E1L1 + H6N5F1E2 + H6N5E1L2 + H6N5E2L1 + H6N5E3 + H6N5F1L3 + H7N6E2 + H6N5F1E1L2 + H6N5F1E2L1 + H9N8 + H6N5F1E3 + H6N5F2E2L1 + H7N6E1L2 + H7N6E2L1 + H7N6E3 + H8N7E2 + H7N6F1E1L2 + H9N8E1 + H7N6E1L3 + H7N6E2L2 + H7N6E3L1 + H7N6F1E1L3 + H7N6F1E2L2 + H9N8E3 ) |
| **CA3** | 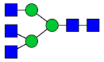 | Relative abundance of triantennary glycans within complex type glycans | CA3 = ( H6N5E1 + H6N5F1E1 + H6N5E1L1 + H6N5E2 + H6N5F1L2 + H6N5F1E1L1 + H6N5F1E2 + H6N5E1L2 + H6N5E2L1 + H6N5E3 + H6N5F1L3 + H6N5F1E1L2 + H6N5F1E2L1 + H6N5F1E3 + H6N5F2E2L1 ) / ( H3N4 + H3N3E1 + H3N4F1 + H4N4 + H3N5 + H3N3F1E1 + H4N3E1 + H3N4F2 + H4N4F1 + H5N4 + H3N5F1 + H3N3F2L1 + H4N5 + H4N3F1L1 + H4N3F1E1 + H4N4L1 + H3N4F1E1 + H5N4F1 + H4N4E1 + H4N5F1 + H5N5 + H5N4L1 + H4N4F1E1 + H5N4E1 + H5N5F1 + H4N5E1 + H5N4F1L1 + H4N4E1L1 + H5N4F1E1 + H4N5F1E1 + H3N4F2L2 + H5N5E1 + H5N4E1L1 + H3N5F1E1L1 + H4N4F1E2 + H5N4E2 + H5N5F1E1 + H4N4F2L2 + H6N5E1 + H5N4F1L2 + H4N6F1E1 + H4N4F2E1L1 + H5N4F1E1L1 + H4N7E1 + H5N4F1E2 + H6N5F1E1 + H5N5E2 + H4N5F2L2 + H6N5E1L1 + H5N5F1E2 + H6N5E2 + H5N5F2L2 + H7N6E1 + H6N5F1L2 + H8N7 + H6N5F1E1L1 + H6N5F1E2 + H6N5E1L2 + H6N5E2L1 + H6N5E3 + H6N5F1L3 + H7N6E2 + H6N5F1E1L2 + H6N5F1E2L1 + H9N8 + H6N5F1E3 + H6N5F2E2L1 + H7N6E1L2 + H7N6E2L1 + H7N6E3 + H8N7E2 + H7N6F1E1L2 + H9N8E1 + H7N6E1L3 + H7N6E2L2 + H7N6E3L1 + H7N6F1E1L3 + H7N6F1E2L2 + H9N8E3 ) |
| **CA4** | 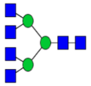 | Relative abundance of tetra-antennary glycans within complex type glycans | CA4 = ( H4N6F1E1 + H7N6E1 + H7N6E2 + H7N6E1L2 + H7N6E2L1 + H7N6E3 + H7N6F1E1L2 + H7N6E1L3 + H7N6E2L2 + H7N6E3L1 + H7N6F1E1L3 + H7N6F1E2L2 ) / ( H3N4 + H3N3E1 + H3N4F1 + H4N4 + H3N5 + H3N3F1E1 + H4N3E1 + H3N4F2 + H4N4F1 + H5N4 + H3N5F1 + H3N3F2L1 + H4N5 + H4N3F1L1 + H4N3F1E1 + H4N4L1 + H3N4F1E1 + H5N4F1 + H4N4E1 + H4N5F1 + H5N5 + H5N4L1 + H4N4F1E1 + H5N4E1 + H5N5F1 + H4N5E1 + H5N4F1L1 + H4N4E1L1 + H5N4F1E1 + H4N5F1E1 + H3N4F2L2 + H5N5E1 + H5N4E1L1 + H3N5F1E1L1 + H4N4F1E2 + H5N4E2 + H5N5F1E1 + H4N4F2L2 + H6N5E1 + H5N4F1L2 + H4N6F1E1 + H4N4F2E1L1 + H5N4F1E1L1 + H4N7E1 + H5N4F1E2 + H6N5F1E1 + H5N5E2 + H4N5F2L2 + H6N5E1L1 + H5N5F1E2 + H6N5E2 + H5N5F2L2 + H7N6E1 + H6N5F1L2 + H8N7 + H6N5F1E1L1 + H6N5F1E2 + H6N5E1L2 + H6N5E2L1 + H6N5E3 + H6N5F1L3 + H7N6E2 + H6N5F1E1L2 + H6N5F1E2L1 + H9N8 + H6N5F1E3 + H6N5F2E2L1 + H7N6E1L2 + H7N6E2L1 + H7N6E3 + H8N7E2 + H7N6F1E1L2 + H9N8E1 + H7N6E1L3 + H7N6E2L2 + H7N6E3L1 + H7N6F1E1L3 + H7N6F1E2L2 + H9N8E3 ) |
| | | **Fucosylation (F)** | |
| **CF** | 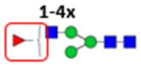  | Fucosylation within complex type glycans | CF = ( H3N4F1 + H3N3F1E1 + H3N4F2 + H4N4F1 + H3N5F1 + H3N3F2L1 + H4N3F1L1 + H4N3F1E1 + H3N4F1E1 + H5N4F1 + H4N5F1 + H4N4F1E1 + H5N5F1 + H5N4F1L1 + H5N4F1E1 + H4N5F1E1 + H3N4F2L2 + H3N5F1E1L1 + H4N4F1E2 + H5N5F1E1 + H4N4F2L2 + H5N4F1L2 + H4N6F1E1 + H4N4F2E1L1 + H5N4F1E1L1 + H5N4F1E2 + H6N5F1E1 + H4N5F2L2 + H5N5F1E2 + H5N5F2L2 + H6N5F1L2 + H6N5F1E1L1 + H6N5F1E2 + H6N5F1L3 + H6N5F1E1L2 + H6N5F1E2L1 + H6N5F1E3 + H6N5F2E2L1 + H7N6F1E1L2 + H7N6F1E1L3 + H7N6F1E2L2 ) / ( H3N4 + H3N3E1 + H3N4F1 + H4N4 + H3N5 + H3N3F1E1 + H4N3E1 + H3N4F2 + H4N4F1 + H5N4 + H3N5F1 + H3N3F2L1 + H4N5 + H4N3F1L1 + H4N3F1E1 + H4N4L1 + H3N4F1E1 + H5N4F1 + H4N4E1 + H4N5F1 + H5N5 + H5N4L1 + H4N4F1E1 + H5N4E1 + H5N5F1 + H4N5E1 + H5N4F1L1 + H4N4E1L1 + H5N4F1E1 + H4N5F1E1 + H3N4F2L2 + H5N5E1 + H5N4E1L1 + H3N5F1E1L1 + H4N4F1E2 + H5N4E2 + H5N5F1E1 + H4N4F2L2 + H6N5E1 + H5N4F1L2 + H4N6F1E1 + H4N4F2E1L1 + H5N4F1E1L1 + H4N7E1 + H5N4F1E2 + H6N5F1E1 + H5N5E2 + H4N5F2L2 + H6N5E1L1 + H5N5F1E2 + H6N5E2 + H5N5F2L2 + H7N6E1 + H6N5F1L2 + H8N7 + |

| | | | |
|---|---|---|---|
| | | | H6N5F1E1L1 + H6N5F1E2 + H6N5E1L2 + H6N5E2L1 + H6N5E3 + H6N5F1L3 + H7N6E2 + H6N5F1E1L2 + H6N5F1E2L1 + H9N8 + H6N5F1E3 + H6N5F2E2L1 + H7N6E1L2 + H7N6E2L1 + H7N6E3 + H8N7E2 + H7N6F1E1L2 + H9N8E1 + H7N6E1L3 + H7N6E2L2 + H7N6E3L1 + H7N6F1E1L3 + H7N6F1E2L2 + H9N8E3 ) |
| **A2F** | 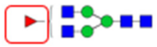 | Fucosylation within diantennary glycans | A2F = ( H3N4F1 + H3N4F2 + H4N4F1 + H3N5F1 + H3N4F1E1 + H5N4F1 + H4N5F1 + H4N4F1E1 + H5N5F1 + H5N4F1L1 + H5N4F1E1 + H4N5F1E1 + H3N4F2L2 + H3N5F1E1L1 + H4N4F1E2 + H5N5F1E1 + H4N4F2L2 + H5N4F1L2 + H4N4F2E1L1 + H5N4F1E1L1 + H5N4F1E2 + H4N5F2L2 + H5N5F1E2 + H5N5F2L2 ) / ( H3N4 + H3N4F1 + H4N4 + H3N5 + H3N4F2 + H4N4F1 + H5N4 + H3N5F1 + H4N5 + H4N4L1 + H3N4F1E1 + H5N4F1 + H4N4E1 + H4N5F1 + H5N5 + H5N4L1 + H4N4F1E1 + H5N4E1 + H5N5F1 + H4N5E1 + H5N4F1L1 + H4N4E1L1 + H5N4F1E1 + H4N5F1E1 + H3N4F2L2 + H5N5E1 + H5N4E1L1 + H3N5F1E1L1 + H4N4F1E2 + H5N4E2 + H5N5F1E1 + H4N4F2L2 + H5N4F1L2 + H4N4F2E1L1 + H5N4F1E1L1 + H5N4F1E2 + H5N5E2 + H4N5F2L2 + H5N5F1E2 + H5N5F2L2 ) |
| **A3F** | 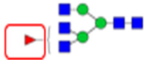 | Fucosylation within triantennary glycans | A3F = ( H6N5F1E1 + H6N5F1L2 + H6N5F1E1L1 + H6N5F1E2 + H6N5F1L3 + H6N5F1E1L2 + H6N5F1E2L1 + H6N5F1E3 + H6N5F2E2L1 ) / ( H6N5E1 + H6N5F1E1 + H6N5E1L1 + H6N5E2 + H6N5F1L2 + H6N5F1E1L1 + H6N5F1E2 + H6N5E1L2 + H6N5E2L1 + H6N5E3 + H6N5F1L3 + H6N5F1E1L2 + H6N5F1E2L1 + H6N5F1E3 + H6N5F2E2L1 ) |
| **A4F** | 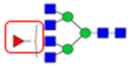 | Fucosylation within tetra-antennary glycans | A4F = ( H4N6F1E1 + H7N6F1E1L2 + H7N6F1E1L3 + H7N6F1E2L2 ) / ( H4N6F1E1 + H7N6E1 + H7N6E2 + H7N6E1L2 + H7N6E2L1 + H7N6E3 + H7N6F1E1L2 + H7N6E1L3 + H7N6E2L2 + H7N6E3L1 + H7N6F1E1L3 + H7N6F1E2L2 ) |
| **A2SF** | 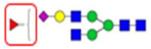 | Fucosylation within sialylated diantennary glycans | A2SF = ( H3N4F1E1 + H4N4F1E1 + H5N4F1L1 + H5N4F1E1 + H4N5F1E1 + H3N4F2L2 + H3N5F1E1L1 + H4N4F1E2 + H5N5F1E1 + H4N4F2L2 + H5N4F1L2 + H4N4F2E1L1 + H5N4F1E1L1 + H5N4F1E2 + H4N5F2L2 + H5N5F1E2 + H5N5F2L2 ) / ( H4N4L1 + H3N4F1E1 + H4N4E1 + H5N4L1 + H4N4F1E1 + H5N4E1 + H4N5E1 + H5N4F1L1 + H4N4E1L1 + H5N4F1E1 + H4N5F1E1 + H3N4F2L2 + H5N5E1 + H5N4E1L1 + H3N5F1E1L1 + H4N4F1E2 + H5N4E2 + H5N5F1E1 + H4N4F2L2 + H5N4F1L2 + H4N4F2E1L1 + H5N4F1E1L1 + H5N4F1E2 + H5N5E2 + H4N5F2L2 + H5N5F1E2 + H5N5F2L2 ) |
| **A2S0F** | 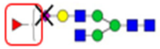 | Fucosylation within non-sialylated diantennary glycans | A2S0F = ( H3N4F1 + H3N4F2 + H4N4F1 + H3N5F1 + H5N4F1 + H4N5F1 + H5N5F1 ) / ( H3N4 + H3N4F1 + H4N4 + H3N5 + H3N4F2 + H4N4F1 + H5N4 + H3N5F1 + H4N5 + H5N4F1 + H4N5F1 + H5N5 + H5N5F1 ) |
| **A2EF** | 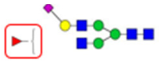 | Fucosylation within diantennary glycans with α2,6-linked sialic acid | A2EF = ( H3N4F1E1 + H4N4F1E1 + H5N4F1E1 + H4N5F1E1 + H3N5F1E1L1 + H4N4F1E2 + H5N5F1E1 + H4N4F2E1L1 + H5N4F1E1L1 + H5N4F1E2 + H5N5F1E2 ) / ( H3N4F1E1 + H4N4E1 + H4N4F1E1 + H5N4E1 + H4N5E1 + H4N4E1L1 + H5N4F1E1 + H4N5F1E1 + H5N5E1 + H5N4E1L1 + H3N5F1E1L1 + H4N4F1E2 + H5N4E2 + H5N5F1E1 + H4N4F2E1L1 + H5N4F1E1L1 + H5N4F1E2 + H5N5E2 + H5N5F1E2 ) |
| **A2E0F** | 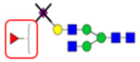 | Fucosylation within diantennary glycans without α2,6-linked sialic acid | A2E0F = ( H3N4F1 + H3N4F2 + H4N4F1 + H3N5F1 + H5N4F1 + H4N5F1 + H5N5F1 + H5N4F1L1 + H3N4F2L2 + H4N4F2L2 + H5N4F1L2 + H4N5F2L2 + H5N5F2L2 ) / ( H3N4 + H3N4F1 + H4N4 + H3N5 + H3N4F2 + H4N4F1 + H5N4 + H3N5F1 + H4N5 + H4N4L1 + H5N4F1 + H4N5F1 + H5N5 + H5N4L1 + H5N5F1 + H5N4F1L1 + H3N4F2L2 + H4N4F2L2 + H5N4F1L2 + H4N5F2L2 + H5N5F2L2 ) |
| **A2LF** | 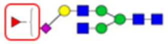 | Fucosylation within diantennary glycans with α2,3-linked sialic acid | A2LF = ( H5N4F1L1 + H3N4F2L2 + H3N5F1E1L1 + H4N4F2L2 + H5N4F1L2 + H4N4F2E1L1 + H5N4F1E1L1 + H4N5F2L2 + H5N5F2L2 ) / ( H4N4L1 + H5N4L1 + H5N4F1L1 + H4N4E1L1 + H3N4F2L2 + H5N4E1L1 + H3N5F1E1L1 + H4N4F2L2 + H5N4F1L2 + H4N4F2E1L1 + H5N4F1E1L1 + H4N5F2L2 + H5N5F2L2 ) |
| **A2L0F** | 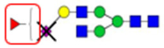 | Fucosylation within diantennary glycans without α2,3-linked sialic acid | A2L0F = ( H3N4F1 + H3N4F2 + H4N4F1 + H3N5F1 + H3N4F1E1 + H5N4F1 + H4N5F1 + H4N4F1E1 + H5N5F1 + H5N4F1E1 + H4N5F1E1 + H4N4F1E2 + H5N5F1E1 + H5N4F1E2 + H5N5F1E2 ) / ( H3N4 + H3N4F1 + H4N4 + H3N5 + H3N4F2 + H4N4F1 + H5N4 + H3N5F1 + H4N5 + H3N4F1E1 + H5N4F1 + H4N4E1 + H4N5F1 + H5N5 + H4N4F1E1 + H5N4E1 + H5N5F1 + H4N5E1 + H5N4F1E1 + H4N5F1E1 + H5N5E1 + H4N4F1E2 + H5N4E2 + H5N5F1E1 + H5N4F1E2 + H5N5E2 + H5N5F1E2 ) |

| | | | |
|---|---|---|---|
| **A3EF** | 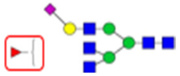 | Fucosylation within triantennary glycans with α2,6-linked sialic acid | A3EF = ( H6N5F1E1 + H6N5F1E1L1 + H6N5F1E2 + H6N5F1E1L2 + H6N5F1E2L1 + H6N5F1E3 + H6N5F2E2L1 ) / ( H6N5E1 + H6N5F1E1 + H6N5E1L1 + H6N5E2 + H6N5F1E1L1 + H6N5F1E2 + H6N5E1L2 + H6N5E2L1 + H6N5E3 + H6N5F1E1L2 + H6N5F1E2L1 + H6N5F1E3 + H6N5F2E2L1 ) |
| **A3LF** | 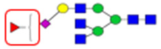 | Fucosylation within triantennary glycans with α2,3-linked sialic acid | A3LF = ( H6N5F1L2 + H6N5F1E1L1 + H6N5F1L3 + H6N5F1E1L2 + H6N5F1E2L1 + H6N5F2E2L1 ) / ( H6N5E1L1 + H6N5F1L2 + H6N5F1E1L1 + H6N5E1L2 + H6N5E2L1 + H6N5F1L3 + H6N5F1E1L2 + H6N5F1E2L1 + H6N5F2E2L1 ) |
| **A3L0F** | 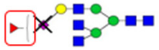 | Fucosylation within triantennary glycans without α2,3-linked sialic acid | A3L0F = ( H6N5F1E1 + H6N5F1E2 + H6N5F1E3 ) / ( H6N5E1 + H6N5F1E1 + H6N5E2 + H6N5F1E2 + H6N5E3 + H6N5F1E3 ) |
| **A4LF** | 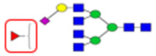 | Fucosylation within tetra-antennary glycans with α2,3-linked sialic acid | A4LF = ( H7N6F1E1L2 + H7N6F1E1L3 + H7N6F1E2L2 ) / ( H7N6E1L2 + H7N6E2L1 + H7N6F1E1L2 + H7N6E1L3 + H7N6E2L2 + H7N6E3L1 + H7N6F1E1L3 + H7N6F1E2L2 ) |
| **A4L0F** | 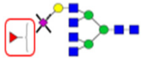 | Fucosylation within tetra-antennary glycans without α2,3-linked sialic acid | A4L0F = ( H4N6F1E1 ) / ( H4N6F1E1 + H7N6E1 + H7N6E2 + H7N6E3 ) |
| | | **Antennary fucosylation (Fa)** | |
| **CFa** | 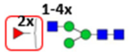 | Relative abundance of species with 2 fucoses (i.e. at least one antennary fucose) within all complex type glycans | CFa = ( H3N4F2 + H3N3F2L1 + H3N4F2L2 + H4N4F2L2 + H4N4F2E1L1 + H4N5F2L2 + H5N5F2L2 + H6N5F2E2L1 ) / ( H3N4 + H3N3E1 + H3N4F1 + H4N4 + H3N5 + H3N3F1E1 + H4N3E1 + H3N4F2 + H4N4F1 + H5N4 + H3N5F1 + H3N3F2L1 + H4N5 + H4N3F1L1 + H4N3F1E1 + H4N4L1 + H3N4F1E1 + H5N4F1 + H4N4E1 + H4N5F1 + H5N5 + H5N4L1 + H4N4F1E1 + H5N4E1 + H5N5F1 + H4N5E1 + H5N4F1L1 + H4N4E1L1 + H5N4F1E1 + H4N5F1E1 + H3N4F2L2 + H5N5E1 + H5N4E1L1 + H3N5F1E1L1 + H4N4F1E2 + H5N4E2 + H5N5F1E1 + H4N4F2L2 + H6N5E1 + H5N4F1L2 + H4N6F1E1 + H4N4F2E1L1 + H5N4F1E1L1 + H4N7E1 + H5N4F1E2 + H6N5F1E1 + H5N5E2 + H4N5F2L2 + H6N5E1L1 + H5N5F1E2 + H6N5E2 + H5N5F2L2 + H7N6E1 + H6N5F1L2 + H8N7 + H6N5F1E1L1 + H6N5F1E2 + H6N5E1L2 + H6N5E2L1 + H6N5E3 + H6N5F1L3 + H7N6E2 + H6N5F1E1L2 + H6N5F1E2L1 + H9N8 + H6N5F1E3 + H6N5F2E2L1 + H7N6E1L2 + H7N6E2L1 + H7N6E3 + H8N7E2 + H7N6F1E1L2 + H9N8E1 + H7N6E1L3 + H7N6E2L2 + H7N6E3L1 + H7N6F1E1L3 + H7N6F1E2L2 + H9N8E3 ) |
| **A2Fa** | 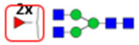 | Relative abundance of species with 2 fucoses (i.e. at least one antennary fucose) within diantennary glycans | A2Fa = ( H3N4F2 + H3N4F2L2 + H4N4F2L2 + H4N4F2E1L1 + H4N5F2L2 + H5N5F2L2 ) / ( H3N4 + H3N4F1 + H4N4 + H3N5 + H3N4F2 + H4N4F1 + H5N4 + H3N5F1 + H4N5 + H4N4L1 + H3N4F1E1 + H5N4F1 + H4N4E1 + H4N5F1 + H5N5 + H5N4L1 + H4N4F1E1 + H5N4E1 + H5N5F1 + H4N5E1 + H5N4F1L1 + H4N4E1L1 + H5N4F1E1 + H4N5F1E1 + H3N4F2L2 + H5N5E1 + H5N4E1L1 + H3N5F1E1L1 + H4N4F1E2 + H5N4E2 + H5N5F1E1 + H4N4F2L2 + H5N4F1L2 + H4N4F2E1L1 + H5N4F1E1L1 + H5N4F1E2 + H5N5E2 + H4N5F2L2 + H5N5F1E2 + H5N5F2L2 ) |
| **A3Fa** | 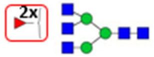 | Relative abundance of species with 2 fucoses (i.e. at least one antennary fucose) within triantennary glycans | A3Fa = ( H6N5F2E2L1 ) / ( H6N5E1 + H6N5F1E1 + H6N5E1L1 + H6N5E2 + H6N5F1L2 + H6N5F1E1L1 + H6N5F1E2 + H6N5E1L2 + H6N5E2L1 + H6N5E3 + H6N5F1L3 + H6N5F1E1L2 + H6N5F1E2L1 + H6N5F1E3 + H6N5F2E2L1 ) |
| | | **Bisection (B)** | |

| | | | |
|---|---|---|---|
| **CB** | 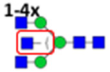 | Relative abundance of species with a bisecting GlcNAc within all complex glycans | CB = ( H3N5 + H3N5F1 + H4N5 + H4N5F1 + H5N5 + H5N5F1 + H4N5E1 + H4N5F1E1 + H5N5E1 + H3N5F1E1L1 + H5N5F1E1 + H5N5E2 + H4N5F2L2 + H5N5F1E2 + H5N5F2L2 ) / ( H3N4 + H3N3E1 + H3N4F1 + H4N4 + H3N5 + H3N3F1E1 + H4N3E1 + H3N4F2 + H4N4F1 + H5N4 + H3N5F1 + H3N3F2L1 + H4N5 + H4N3F1L1 + H4N3F1E1 + H4N4L1 + H3N4F1E1 + H5N4F1 + H4N4E1 + H4N5F1 + H5N5 + H5N4L1 + H4N4F1E1 + H5N4E1 + H5N5F1 + H4N5E1 + H5N4F1L1 + H4N4E1L1 + H5N4F1E1 + H4N5F1E1 + H3N4F2L2 + H5N5E1 + H5N4E1L1 + H3N5F1E1L1 + H4N4F1E2 + H5N4E2 + H5N5F1E1 + H4N4F2L2 + H6N5E1 + H5N4F1L2 + H4N6F1E1 + H4N4F2E1L1 + H5N4F1E1L1 + H4N7E1 + H5N4F1E2 + H6N5F1E1 + H5N5E2 + H4N5F2L2 + H6N5E1L1 + H5N5F1E2 + H6N5E2 + H5N5F2L2 + H7N6E1 + H6N5F1L2 + H8N7 + H6N5F1E1L1 + H6N5F1E2 + H6N5E1L2 + H6N5E2L1 + H6N5E3 + H6N5F1L3 + H7N6E2 + H6N5F1E1L2 + H6N5F1E2L1 + H9N8 + H6N5F1E3 + H6N5F2E2L1 + H7N6E1L2 + H7N6E2L1 + H7N6E3 + H8N7E2 + H7N6F1E1L2 + H9N8E1 + H7N6E1L3 + H7N6E2L2 + H7N6E3L1 + H7N6F1E1L3 + H7N6F1E2L2 + H9N8E3 ) |
| **A2B** | 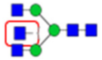 | Relative abundance of species with a bisecting GlcNAc within diantennary glycans | A2B = ( H3N5 + H3N5F1 + H4N5 + H4N5F1 + H5N5 + H5N5F1 + H4N5E1 + H4N5F1E1 + H5N5E1 + H3N5F1E1L1 + H5N5F1E1 + H5N5E2 + H4N5F2L2 + H5N5F1E2 + H5N5F2L2 ) / ( H3N4 + H3N4F1 + H4N4 + H3N5 + H3N4F2 + H4N4F1 + H5N4 + H3N5F1 + H4N5 + H4N4L1 + H3N4F1E1 + H5N4F1 + H4N4E1 + H4N5F1 + H5N5 + H5N4L1 + H4N4F1E1 + H5N4E1 + H5N5F1 + H4N5E1 + H5N4F1L1 + H4N4E1L1 + H5N4F1E1 + H4N5F1E1 + H3N4F2L2 + H5N5E1 + H5N4E1L1 + H3N5F1E1L1 + H4N4F1E2 + H5N4E2 + H5N5F1E1 + H4N4F2L2 + H5N4F1L2 + H4N4F2E1L1 + H5N4F1E1L1 + H5N4F1E2 + H5N5E2 + H4N5F2L2 + H5N5F1E2 + H5N5F2L2 ) |
| **A2FB** | 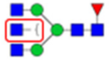 | Relative abundance of species with a bisecting GlcNAc within fucosylated diantennary | A2FB = ( H3N5F1 + H4N5F1 + H5N5F1 + H4N5F1E1 + H3N5F1E1L1 + H5N5F1E1 + H4N5F2L2 + H5N5F1E2 + H5N5F2L2 ) / ( H3N4F1 + H3N4F2 + H4N4F1 + H3N5F1 + H3N4F1E1 + H5N4F1 + H4N5F1 + H4N4F1E1 + H5N5F1 + H5N4F1L1 + H5N4F1E1 + H4N5F1E1 + H3N4F2L2 + H3N5F1E1L1 + H4N4F1E2 + H5N5F1E1 + H4N4F2L2 + H5N4F1L2 + H4N4F2E1L1 + H5N4F1E1L1 + H5N4F1E2 + H4N5F2L2 + H5N5F1E2 + H5N5F2L2 ) |
| **A2F0B** | 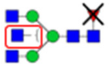 | Relative abundance of species with a bisecting GlcNAc within non-fucosylated dianten nary glycans | A2F0B = ( H3N5 + H4N5 + H5N5 + H4N5E1 + H5N5E1 + H5N5E2 ) / ( H3N4 + H4N4 + H3N5 + H5N4 + H4N5 + H4N4L1 + H4N4E1 + H5N5 + H5N4L1 + H5N4E1 + H4N5E1 + H4N4E1L1 + H5N5E1 + H5N4E1L1 + H5N4E2 + H5N5E2 ) |
| **A2SB** | 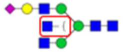 | Relative abundance of species with a bisecting GlcNAc within sialylated diantennary glycans | A2SB = ( H4N5E1 + H4N5F1E1 + H5N5E1 + H3N5F1E1L1 + H5N5F1E1 + H5N5E2 + H4N5F2L2 + H5N5F1E2 + H5N5F2L2 ) / ( H4N4L1 + H3N4F1E1 + H4N4E1 + H5N4L1 + H4N4F1E1 + H5N4E1 + H4N5E1 + H5N4F1L1 + H4N4E1L1 + H5N4F1E1 + H4N5F1E1 + H3N4F2L2 + H5N5E1 + H5N4E1L1 + H3N5F1E1L1 + H4N4F1E2 + H5N4E2 + H5N5F1E1 + H4N4F2L2 + H5N4F1L2 + H4N4F2E1L1 + H5N4F1E1L1 + H5N4F1E2 + H5N5E2 + H4N5F2L2 + H5N5F1E2 + H5N5F2L2 ) |
| **A2S0B** | 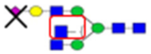 | Relative abundance of species with a bisecting GlcNAc within non-sialylated diantennary glycans | A2S0B = ( H3N5 + H3N5F1 + H4N5 + H4N5F1 + H5N5 + H5N5F1 ) / ( H3N4 + H3N4F1 + H4N4 + H3N5 + H3N4F2 + H4N4F1 + H5N4 + H3N5F1 + H4N5 + H5N4F1 + H4N5F1 + H5N5 + H5N5F1 ) |
| **A2FSB** | 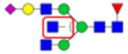 | Relative abundance of species with a bisecting GlcNAc within fucosylated sialylated diantennary glycans | A2FSB = ( H4N5F1E1 + H3N5F1E1L1 + H5N5F1E1 + H4N5F2L2 + H5N5F1E2 + H5N5F2L2 ) / ( H3N4F1E1 + H4N4F1E1 + H5N4F1L1 + H5N4F1E1 + H4N5F1E1 + H3N4F2L2 + H3N5F1E1L1 + H4N4F1E2 + H5N5F1E1 + H4N4F2L2 + H5N4F1L2 + H4N4F2E1L1 + H5N4F1E1L1 + H5N4F1E2 + H4N5F2L2 + H5N5F1E2 + H5N5F2L2 ) |
| **A2F0SB** | 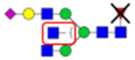 | Relative abundance of species with a bisecting GlcNAc within non-fucosylated sialylated diantennary glycans | A2F0SB = ( H4N5E1 + H5N5E1 + H5N5E2 ) / ( H4N4L1 + H4N4E1 + H5N4L1 + H5N4E1 + H4N5E1 + H4N4E1L1 + H5N5E1 + H5N4E1L1 + H5N4E2 + H5N5E2 ) |

| | | | |
|---|---|---|---|
| **A2FS0B** | 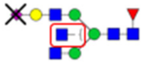 | Relative abundance of species with a bisecting GlcNAc within fucosylated non-sialylated diantennary glycans | A2FS0B = ( H3N5F1 + H4N5F1 + H5N5F1 ) / ( H3N4F1 + H3N4F2 + H4N4F1 + H3N5F1 + H5N4F1 + H4N5F1 + H5N5F1 ) |
| **A2F0S0B** | 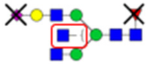 | Relative abundance of species with a bisecting GlcNAc within non-fucosylated non-sialylated diantennary glycans | A2F0S0B = ( H3N5 + H4N5 + H5N5 ) / ( H3N4 + H4N4 + H3N5 + H5N4 + H4N5 + H5N5 ) |
| | | **Galactosylaiton (G)** | |
| **CG** | 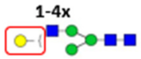 | Galactosylation within all complex glycans | CG = ( H3N3E1 + H4N4 + H3N3F1E1 + H4N3E1 + H4N4F1 + H5N4 + H3N3F2L1 + H4N5 + H4N3F1L1 + H4N3F1E1 + H4N4L1 + H3N4F1E1 + H5N4F1 + H4N4E1 + H4N5F1 + H5N5 + H5N4L1 + H4N4F1E1 + H5N4E1 + H4N5E1 + H5N4F1L1 + H4N4E1L1 + H5N4F1E1 + H4N5F1E1 + H3N4F2L2 + H5N5E1 + H5N4E1L1 + H3N5F1E1L1 + H4N4F1E2 + H5N4E2 + H5N5F1E1 + H4N4F2L2 + H6N5E1 + H5N4F1L2 + H4N6F1E1 + H4N4F2E1L1 + H5N4F1E1L1 + H4N7E1 + H5N4F1E2 + H6N5F1E1 + H5N5E2 + H4N5F2L2 + H6N5E1L1 + H5N5F1E2 + H6N5E2 + H5N5F2L2 + H7N6E1 + H6N5F1L2 + H8N7 + H6N5F1E1L1 + H6N5F1E2 + H6N5E1L2 + H6N5E2L1 + H6N5E3 + H6N5F1L3 + H7N6E2 + H6N5F1E1L2 + H6N5F1E2L1 + H9N8 + H6N5F1E3 + H6N5F2E2L1 + H7N6E1L2 + H7N6E2L1 + H7N6E3 + H8N7E2 + H7N6F1E1L2 + H9N8E1 + H7N6E1L3 + H7N6E2L2 + H7N6E3L1 + H7N6F1E1L3 + H7N6F1E2L2 + H9N8E3 ) / ( H3N4 + H3N3E1 + H3N4F1 + H4N4 + H3N5 + H3N3F1E1 + H4N3E1 + H3N4F2 + H4N4F1 + H5N4 + H3N5F1 + H3N3F2L1 + H4N5 + H4N3F1L1 + H4N3F1E1 + H4N4L1 + H3N4F1E1 + H5N4F1 + H4N4E1 + H4N5F1 + H5N5 + H5N4L1 + H4N4F1E1 + H5N4E1 + H5N5F1 + H4N5E1 + H5N4F1L1 + H4N4E1L1 + H5N4F1E1 + H4N5F1E1 + H3N4F2L2 + H5N5E1 + H5N4E1L1 + H3N5F1E1L1 + H4N4F1E2 + H5N4E2 + H5N5F1E1 + H4N4F2L2 + H6N5E1 + H5N4F1L2 + H4N6F1E1 + H4N4F2E1L1 + H5N4F1E1L1 + H4N7E1 + H5N4F1E2 + H6N5F1E1 + H5N5E2 + H4N5F2L2 + H6N5E1L1 + H5N5F1E2 + H6N5E2 + H5N5F2L2 + H7N6E1 + H6N5F1L2 + H8N7 + H6N5F1E1L1 + H6N5F1E2 + H6N5E1L2 + H6N5E2L1 + H6N5E3 + H6N5F1L3 + H7N6E2 + H6N5F1E1L2 + H6N5F1E2L1 + H9N8 + H6N5F1E3 + H6N5F2E2L1 + H7N6E1L2 + H7N6E2L1 + H7N6E3 + H8N7E2 + H7N6F1E1L2 + H9N8E1 + H7N6E1L3 + H7N6E2L2 + H7N6E3L1 + H7N6F1E1L3 + H7N6F1E2L2 + H9N8E3 ) |
| **A2G** | 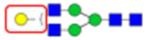 | Galactosylation per antenna within diantennary glycans | A2G = ( 0/2 * ( H3N4 + H3N4F1 + H3N5 + H3N4F2 + H3N5F1 ) + 1/2 * ( H4N4 + H4N4F1 + H4N5 + H4N4L1 + H4N4E1 + H4N5F1 + H4N4F1E1 + H4N5E1 + H4N4E1L1 + H4N5F1E1 + H4N4F1E2 + H4N4F2L2 + H4N4F2E1L1 + H4N5F2L2 ) + 2/2 * ( H5N4 + H5N4F1 + H5N5 + H5N4L1 + H5N4E1 + H5N5F1 + H5N4F1L1 + H4N4E1L1 + H5N4F1E1 + H3N4F2L2 + H5N5E1 + H5N4E1L1 + H4N4F1E2 + H5N4E2 + H5N5F1E1 + H4N4F2L2 + H5N4F1L2 + H4N4F2E1L1 + H5N4F1E1L1 + H5N4F1E2 + H5N5E2 + H5N5F1E2 + H5N5F2L2 ) ) / ( H3N4 + H3N4F1 + H4N4 + H3N5 + H3N4F2 + H4N4F1 + H5N4 + H3N5F1 + H4N5 + H4N4L1 + H3N4F1E1 + H5N4F1 + H4N4E1 + H4N5F1 + H5N5 + H5N4L1 + H4N4F1E1 + H5N4E1 + H5N5F1 + H4N5E1 + H5N4F1L1 + H4N4E1L1 + H5N4F1E1 + H4N5F1E1 + H3N4F2L2 + H5N5E1 + H5N4E1L1 + H3N5F1E1L1 + H4N4F1E2 + H5N4E2 + H5N5F1E1 + H4N4F2L2 + H5N4F1L2 + H4N4F2E1L1 + H5N4F1E1L1 + H5N4F1E2 + H5N5E2 + H4N5F2L2 + H5N5F1E2 + H5N5F2L2 ) |
| **A4G** | 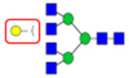 | Galactosylation per antenna within tetra-antennary glycans | A4G = ( 0/4 * (0) + 1/4 * ( H4N6F1E1 ) + 2/4 * (0) + 3/4 * (0) + 4/4 * ( H7N6E1 + H7N6E2 + H7N6E1L2 + H7N6E2L1 + H7N6E3 + H7N6F1E1L2 + H7N6E1L3 + H7N6E2L2 + H7N6E3L1 + H7N6F1E1L3 + H7N6F1E2L2 ) ) / ( H4N6F1E1 + H7N6E1 + H7N6E2 + H7N6E1L2 + H7N6E2L1 + H7N6E3 + H7N6F1E1L2 + H7N6E1L3 + H7N6E2L2 + H7N6E3L1 + H7N6F1E1L3 + H7N6F1E2L2 ) |
| **A2FG** | 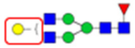 | Galactosylation per antenna within fucosylated diantennary glycans | A2FG = ( 0/2 * ( H3N4F1 + H3N4F2 + H3N5F1 ) + 1/2 * ( H4N4F1 + H4N5F1 + H4N4F1E1 + H4N5F1E1 + H4N4F1E2 + H4N4F2L2 + H4N4F2E1L1 + H4N5F2L2 ) + 2/2 * ( H5N4F1 + H5N5F1 + H5N4F1L1 + H5N4F1E1 + H3N4F2L2 + H4N4F1E2 + H5N5F1E1 + H4N4F2L2 + H5N4F1L2 + H4N4F2E1L1 + H5N4F1E1L1 + H5N4F1E2 + H5N5F1E2 + H5N5F2L2 ) ) / ( H3N4F1 + H3N4F2 + H4N4F1 + H3N5F1 + H3N4F1E1 + H5N4F1 + H4N5F1 + H4N4F1E1 + H5N5F1 + H5N4F1L1 + H5N4F1E1 + H4N5F1E1 + H3N4F2L2 + H3N5F1E1L1 + H4N4F1E2 + H5N5F1E1 + H4N4F2L2 + H5N4F1L2 + H4N4F2E1L1 + H5N4F1E1L1 + H5N4F1E2 + H4N5F2L2 + H5N5F1E2 + H5N5F2L2 ) |

| | | | |
|---|---|---|---|
| **A2F0G** | 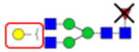 | Galactosylation per antenna within non-fucosylated diantennary glycans | A2F0G = ( 0/2 * ( H3N4 + H3N5 ) + 1/2 * ( H4N4 + H4N5 + H4N4L1 + H4N4E1 + H4N5E1 + H4N4E1L1 ) + 2/2 * ( H5N4 + H5N5 + H5N4L1 + H5N4E1 + H4N4E1L1 + H5N5E1 + H5N4E1L1 + H5N4E2 + H5N5E2 ) ) / ( H3N4 + H4N4 + H3N5 + H5N4 + H4N5 + H4N4L1 + H4N4E1 + H5N5 + H5N4L1 + H5N4E1 + H4N5E1 + H4N4E1L1 + H5N5E1 + H5N4E1L1 + H5N4E2 + H5N5E2 ) |
| **A2SG** | 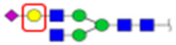 | Galactosylation per antenna within sialylated diantennary glycans | A2SG = ( 0/2 * (0) + 1/2 * ( H4N4L1 + H4N4E1 + H4N4F1E1 + H4N5E1 + H4N4E1L1 + H4N5F1E1 + H4N4F1E2 + H4N4F2L2 + H4N4F2E1L1 + H4N5F2L2 ) + 2/2 * ( H5N4L1 + H5N4E1 + H5N4F1L1 + H4N4E1L1 + H5N4F1E1 + H3N4F2L2 + H5N5E1 + H5N4E1L1 + H4N4F1E2 + H5N4E2 + H5N5F1E1 + H4N4F2L2 + H5N4F1L2 + H4N4F2E1L1 + H5N4F1E1L1 + H5N4F1E2 + H5N5E2 + H5N5F1E2 + H5N5F2L2 ) ) / ( H4N4L1 + H3N4F1E1 + H4N4E1 + H5N4L1 + H4N4F1E1 + H5N4E1 + H4N5E1 + H5N4F1L1 + H4N4E1L1 + H5N4F1E1 + H4N5F1E1 + H3N4F2L2 + H5N5E1 + H5N4E1L1 + H3N5F1E1L1 + H4N4F1E2 + H5N4E2 + H5N5F1E1 + H4N4F2L2 + H5N4F1L2 + H4N4F2E1L1 + H5N4F1E1L1 + H5N4F1E2 + H5N5E2 + H4N5F2L2 + H5N5F1E2 + H5N5F2L2 ) |
| **A2S0G** | 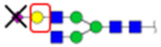 | Galactosylation per antenna within non-sialylated diantennary glycans | A2S0G = ( 0/2 * ( H3N4 + H3N4F1 + H3N5 + H3N4F2 + H3N5F1 ) + 1/2 * ( H4N4 + H4N4F1 + H4N5 + H4N5F1 ) + 2/2 * ( H5N4 + H5N4F1 + H5N5 + H5N5F1 ) ) / ( H3N4 + H3N4F1 + H4N4 + H3N5 + H3N4F2 + H4N4F1 + H5N4 + H3N5F1 + H4N5 + H5N4F1 + H4N5F1 + H5N5 + H5N5F1 ) |
| **A2FSG** | 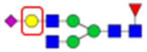 | Galactosylation per antenna within fucosylated sialylated diantennary glycans | A2FSG = ( 0/2 * (0) + 1/2 * ( H4N4F1E1 + H4N5F1E1 + H4N4F1E2 + H4N4F2L2 + H4N4F2E1L1 + H4N5F2L2 ) + 2/2 * ( H5N4F1L1 + H5N4F1E1 + H3N4F2L2 + H4N4F1E2 + H5N5F1E1 + H4N4F2L2 + H5N4F1L2 + H4N4F2E1L1 + H5N4F1E1L1 + H5N4F1E2 + H5N5F1E2 + H5N5F2L2 ) ) / ( H3N4F1E1 + H4N4F1E1 + H5N4F1L1 + H5N4F1E1 + H4N5F1E1 + H3N4F2L2 + H3N5F1E1L1 + H4N4F1E2 + H5N5F1E1 + H4N4F2L2 + H5N4F1L2 + H4N4F2E1L1 + H5N4F1E1L1 + H5N4F1E2 + H4N5F2L2 + H5N5F1E2 + H5N5F2L2 ) |
| **A2F0SG** | 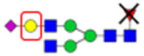 | Galactosylation per antenna within non-fucosylated sialylated diantennary glycans | A2F0SG = ( 0/2 * (0) + 1/2 * ( H4N4L1 + H4N4E1 + H4N5E1 + H4N4E1L1 ) + 2/2 * ( H5N4L1 + H5N4E1 + H4N4E1L1 + H5N5E1 + H5N4E1L1 + H5N4E2 + H5N5E2 ) ) / ( H4N4L1 + H4N4E1 + H5N4L1 + H5N4E1 + H4N5E1 + H4N4E1L1 + H5N5E1 + H5N4E1L1 + H5N4E2 + H5N5E2 ) |
| **A2FS0G** | 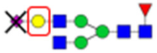 | Galactosylation per antenna within fucosylated non-sialylated diantennary glycans | A2FS0G = ( 0/2 * ( H3N4F1 + H3N4F2 + H3N5F1 ) + 1/2 * ( H4N4F1 + H4N5F1 ) + 2/2 * ( H5N4F1 + H5N5F1 ) ) / ( H3N4F1 + H3N4F2 + H4N4F1 + H3N5F1 + H5N4F1 + H4N5F1 + H5N5F1 ) |
| **A2F0S0G** | 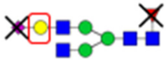 | Galactosylation per antenna within non-fucosylated, non-sialylated diantennary glycans | A2F0S0G = ( 0/2 * ( H3N4 + H3N5 ) + 1/2 * ( H4N4 + H4N5 ) + 2/2 * ( H5N4 + H5N5 ) ) / ( H3N4 + H4N4 + H3N5 + H5N4 + H4N5 + H5N5 ) |
| **A4F0G** | 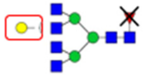 | Galactosylation per antenna within non-fucosylated tetra-antennary glycans | A4F0G = ( 0/4 * (0) + 1/4 * (0) + 2/4 * (0) + 3/4 * (0) + 4/4 * ( H7N6E1 + H7N6E2 + H7N6E1L2 + H7N6E2L1 + H7N6E3 + H7N6E1L3 + H7N6E2L2 + H7N6E3L1 ) ) / ( H4N6F1E1 + H7N6E1 + H7N6E2 + H7N6E1L2 + H7N6E2L1 + H7N6E3 + H7N6F1E1L2 + H7N6E1L3 + H7N6E2L2 + H7N6E3L1 + H7N6F1E1L3 + H7N6F1E2L2 ) |
| | | **Sialylation (S)** | |
| **CS** | 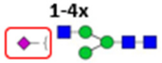 | Sialylation per antenna within all complex glycans | CS = ( H3N3E1 + H3N3F1E1 + H4N3E1 + H3N3F2L1 + H4N3F1L1 + H4N3F1E1 + H4N4L1 + H3N4F1E1 + H4N4E1 + H5N4L1 + H4N4F1E1 + H5N4E1 + H4N5E1 + H5N4F1L1 + H4N4E1L1 + H5N4F1E1 + H4N5F1E1 + H3N4F2L2 + H5N5E1 + H5N4E1L1 + H3N5F1E1L1 + H4N4F1E2 + H5N4E2 + H5N5F1E1 + H4N4F2L2 + H6N5E1 + H5N4F1L2 + H4N6F1E1 + H4N4F2E1L1 + H5N4F1E1L1 + H4N7E1 + H5N4F1E2 + H6N5F1E1 + H5N5E2 + H4N5F2L2 + H6N5E1L1 + H5N5F1E2 + H6N5E2 + H5N5F2L2 + H7N6E1 + H6N5F1L2 + H6N5F1E1L1 + H6N5F1E2 + H6N5E1L2 + H6N5E2L1 + H6N5E3 + H6N5F1L3 + H7N6E2 + H6N5F1E1L2 + H6N5F1E2L1 + H6N5F1E3 + H6N5F2E2L1 + H7N6E1L2 + H7N6E2L1 + H7N6E3 + H8N7E2 + H7N6F1E1L2 + H9N8E1 + H7N6E1L3 + H7N6E2L2 + H7N6E3L1 + H7N6F1E1L3 + H7N6F1E2L2 + H9N8E3 ) / ( H3N4 + H3N3E1 + H3N4F1 + H4N4 + H3N5 + H3N3F1E1 + H4N3E1 + H3N4F2 + H4N4F1 + H5N4 + H3N5F1 + H3N3F2L1 + H4N5 + H4N3F1L1 + |

| | | | |
|---|---|---|---|
| | | | H4N3F1E1 + H4N4L1 + H3N4F1E1 + H5N4F1 + H4N4E1 + H4N5F1 + H5N5 + H5N4L1 + H4N4F1E1 + H5N4E1 + H5N5F1 + H4N5E1 + H5N4F1L1 + H4N4E1L1 + H5N4F1E1 + H4N5F1E1 + H3N4F2L2 + H5N5E1 + H5N4E1L1 + H3N5F1E1L1 + H4N4F1E2 + H5N4E2 + H5N5F1E1 + H4N4F2L2 + H6N5E1 + H5N4F1L2 + H4N6F1E1 + H4N4F2E1L1 + H5N4F1E1L1 + H4N7E1 + H5N4F1E2 + H6N5F1E1 + H5N5E2 + H4N5F2L2 + H6N5E1L1 + H5N5F1E2 + H6N5E2 + H5N5F2L2 + H7N6E1 + H6N5F1L2 + H8N7 + H6N5F1E1L1 + H6N5F1E2 + H6N5E1L2 + H6N5E2L1 + H6N5E3 + H6N5F1L3 + H7N6E2 + H6N5F1E1L2 + H6N5F1E2L1 + H9N8 + H6N5F1E3 + H6N5F2E2L1 + H7N6E1L2 + H7N6E2L1 + H7N6E3 + H8N7E2 + H7N6F1E1L2 + H9N8E1 + H7N6E1L3 + H7N6E2L2 + H7N6E3L1 + H7N6F1E1L3 + H7N6F1E2L2 + H9N8E3 ) |
| **A2S** | 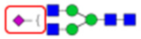 | Sialylation per antenna within diantennary glycans | A2S = ( 0/2 * ( H3N4 + H3N4F1 + H4N4 + H3N5 + H3N4F2 + H4N4F1 + H5N4 + H3N5F1 + H4N5 + H5N4F1 + H4N5F1 + H5N5 + H5N5F1 ) + 1/2 * ( H4N4L1 + H3N4F1E1 + H4N4E1 + H5N4L1 + H4N4F1E1 + H5N4E1 + H4N5E1 + H5N4F1L1 + H5N4F1E1 + H4N5F1E1 + H5N5E1 + H5N5F1E1 ) + 2/2 * ( H4N4E1L1 + H3N4F2L2 + H5N4E1L1 + H3N5F1E1L1 + H4N4F1E2 + H5N4E2 + H4N4F2L2 + H5N4F1L2 + H4N4F2E1L1 + H5N4F1E1L1 + H5N4F1E2 + H5N5E2 + H4N5F2L2 + H5N5F1E2 + H5N5F2L2 ) ) / ( H3N4 + H3N4F1 + H4N4 + H3N5 + H3N4F2 + H4N4F1 + H5N4 + H3N5F1 + H4N5 + H4N4L1 + H3N4F1E1 + H5N4F1 + H4N4E1 + H4N5F1 + H5N5 + H5N4L1 + H4N4F1E1 + H5N4E1 + H5N5F1 + H4N5E1 + H5N4F1L1 + H4N4E1L1 + H5N4F1E1 + H4N5F1E1 + H3N4F2L2 + H5N5E1 + H5N4E1L1 + H3N5F1E1L1 + H4N4F1E2 + H5N4E2 + H5N5F1E1 + H4N4F2L2 + H5N4F1L2 + H4N4F2E1L1 + H5N4F1E1L1 + H5N4F1E2 + H5N5E2 + H4N5F2L2 + H5N5F1E2 + H5N5F2L2 ) |
| **A3S** | 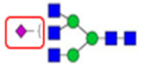 | Sialylation per antenna within triantennary glycans | A3S = ( 0/3 * (0) + 1/3 * ( H6N5E1 + H6N5F1E1 ) + 2/3 * ( H6N5E1L1 + H6N5E2 + H6N5F1L2 + H6N5F1E1L1 + H6N5F1E2 ) + 3/3 * ( H6N5E1L2 + H6N5E2L1 + H6N5E3 + H6N5F1L3 + H6N5F1E1L2 + H6N5F1E2L1 + H6N5F1E3 + H6N5F2E2L1 ) ) / ( H6N5E1 + H6N5F1E1 + H6N5E1L1 + H6N5E2 + H6N5F1L2 + H6N5F1E1L1 + H6N5F1E2 + H6N5E1L2 + H6N5E2L1 + H6N5E3 + H6N5F1L3 + H6N5F1E1L2 + H6N5F1E2L1 + H6N5F1E3 + H6N5F2E2L1 ) |
| **A4S** | 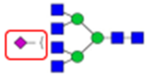 | Sialylation per antenna within tetra-antennary glycans | A4S = ( 0/4 * (0) + 1/4 * ( H4N6F1E1 + H7N6E1 ) + 2/4 * ( H7N6E2 ) + 3/4 * ( H7N6E1L2 + H7N6E2L1 + H7N6E3 + H7N6F1E1L2 ) + 4/4 * ( H7N6E1L3 + H7N6E2L2 + H7N6E3L1 + H7N6F1E1L3 + H7N6F1E2L2 ) ) / ( H4N6F1E1 + H7N6E1 + H7N6E2 + H7N6E1L2 + H7N6E2L1 + H7N6E3 + H7N6F1E1L2 + H7N6E1L3 + H7N6E2L2 + H7N6E3L1 + H7N6F1E1L3 + H7N6F1E2L2 ) |
| **A2FS** | 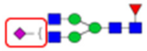 | Sialylation per antenna within fucosylated diantennary glycans | A2FS = ( 0/2 * ( H3N4F1 + H3N4F2 + H4N4F1 + H3N5F1 + H5N4F1 + H4N5F1 + H5N5F1 ) + 1/2 * ( H3N4F1E1 + H4N4F1E1 + H5N4F1L1 + H5N4F1E1 + H4N5F1E1 + H5N5F1E1 ) + 2/2 * ( H3N4F2L2 + H3N5F1E1L1 + H4N4F1E2 + H4N4F2L2 + H5N4F1L2 + H4N4F2E1L1 + H5N4F1E1L1 + H5N4F1E2 + H4N5F2L2 + H5N5F1E2 + H5N5F2L2 ) ) / ( H3N4F1 + H3N4F2 + H4N4F1 + H3N5F1 + H3N4F1E1 + H5N4F1 + H4N5F1 + H4N4F1E1 + H5N5F1 + H5N4F1L1 + H5N4F1E1 + H4N5F1E1 + H3N4F2L2 + H3N5F1E1L1 + H4N4F1E2 + H5N5F1E1 + H4N4F2L2 + H5N4F1L2 + H4N4F2E1L1 + H5N4F1E1L1 + H5N4F1E2 + H4N5F2L2 + H5N5F1E2 + H5N5F2L2 ) |
| **A2F0S** | 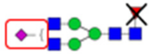 | Sialylation per antenna within non-fucosylated diantennary glycans | A2F0S = ( 0/2 * ( H3N4 + H4N4 + H3N5 + H5N4 + H4N5 + H5N5 ) + 1/2 * ( H4N4L1 + H4N4E1 + H5N4L1 + H5N4E1 + H4N5E1 + H5N5E1 ) + 2/2 * ( H4N4E1L1 + H5N4E1L1 + H5N4E2 + H5N5E2 ) ) / ( H3N4 + H4N4 + H3N5 + H5N4 + H4N5 + H4N4L1 + H4N4E1 + H5N5 + H5N4L1 + H5N4E1 + H4N5E1 + H4N4E1L1 + H5N5E1 + H5N4E1L1 + H5N4E2 + H5N5E2 ) |
| **A3FS** | 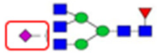 | Sialylation per antenna within fucosylated triantennary glycans | A3FS = ( 0/3 * (0) + 1/3 * ( H6N5F1E1 ) + 2/3 * ( H6N5F1L2 + H6N5F1E1L1 + H6N5F1E2 ) + 3/3 * ( H6N5F1L3 + H6N5F1E1L2 + H6N5F1E2L1 + H6N5F1E3 + H6N5F2E2L1 ) ) / ( H6N5F1E1 + H6N5F1L2 + H6N5F1E1L1 + H6N5F1E2 + H6N5F1L3 + H6N5F1E1L2 + H6N5F1E2L1 + H6N5F1E3 + H6N5F2E2L1 ) |
| **A3F0S** | 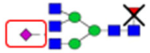 | Sialylation per antenna within non-fucosylated triantennary glycans | A3F0S = ( 0/3 * (0) + 1/3 * ( H6N5E1 ) + 2/3 * ( H6N5E1L1 + H6N5E2 ) + 3/3 * ( H6N5E1L2 + H6N5E2L1 + H6N5E3 ) ) / ( H6N5E1 + H6N5E1L1 + H6N5E2 + H6N5E1L2 + H6N5E2L1 + H6N5E3 ) |
| **A4FS** | 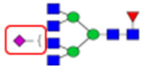 | Sialylation per antenna within fucosylated tetra-antennary glycans | A4FS = ( 0/4 * (0) + 1/4 * ( H4N6F1E1 ) + 2/4 * (0) + 3/4 * ( H7N6F1E1L2 ) + 4/4 * ( H7N6F1E1L3 + H7N6F1E2L2 ) ) / ( H4N6F1E1 + H7N6E1 + H7N6E2 + H7N6E1L2 + H7N6E2L1 + H7N6E3 + H7N6F1E1L2 + H7N6E1L3 + H7N6E2L2 + H7N6E3L1 + H7N6F1E1L3 + H7N6F1E2L2 ) |

| | | | |
|---|---|---|---|
| **A4F0S** | 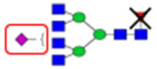 | Sialylation per antenna within non-fucosylated tetra-antennary glycans | A4F0S = ( 0/4 * (0) + 1/4 * ( H7N6E1 ) + 2/4 * ( H7N6E2 ) + 3/4 * ( H7N6E1L2 + H7N6E2L1 + H7N6E3 ) + 4/4 * ( H7N6E1L3 + H7N6E2L2 + H7N6E3L1 ) ) / ( H4N6F1E1 + H7N6E1 + H7N6E2 + H7N6E1L2 + H7N6E2L1 + H7N6E3 + H7N6F1E1L2 + H7N6E1L3 + H7N6E2L2 + H7N6E3L1 + H7N6F1E1L3 + H7N6F1E2L2 ) |
| **A2GS** | 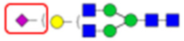 | Sialylation per galactose within diantennary glycans | A2GS = ( ( 0/2 * ( H3N4 + H3N4F1 + H4N4 + H3N5 + H3N4F2 + H4N4F1 + H5N4 + H3N5F1 + H4N5 + H5N4F1 + H4N5F1 + H5N5 + H5N5F1 ) + 1/2 * ( H4N4L1 + H3N4F1E1 + H4N4E1 + H5N4L1 + H4N4F1E1 + H5N4E1 + H4N5E1 + H5N4F1L1 + H5N4F1E1 + H4N5F1E1 + H5N5E1 + H5N5F1E1 ) + 2/2 * ( H4N4E1L1 + H3N4F2L2 + H5N4E1L1 + H3N5F1E1L1 + H4N4F1E2 + H5N4E2 + H4N4F2L2 + H5N4F1L2 + H4N4F2E1L1 + H5N4F1E1L1 + H5N4F1E2 + H5N5E2 + H4N5F2L2 + H5N5F1E2 + H5N5F2L2 ) ) / ( H3N4 + H3N4F1 + H4N4 + H3N5 + H3N4F2 + H4N4F1 + H5N4 + H3N5F1 + H4N5 + H4N4L1 + H3N4F1E1 + H5N4F1 + H4N4E1 + H4N5F1 + H5N5 + H5N4L1 + H4N4F1E1 + H5N4E1 + H5N5F1 + H4N5E1 + H5N4F1L1 + H4N4E1L1 + H5N4F1E1 + H4N5F1E1 + H3N4F2L2 + H5N5E1 + H5N4E1L1 + H3N5F1E1L1 + H4N4F1E2 + H5N4E2 + H5N5F1E1 + H4N4F2L2 + H5N4F1L2 + H4N4F2E1L1 + H5N4F1E1L1 + H5N4F1E2 + H5N5E2 + H4N5F2L2 + H5N5F1E2 + H5N5F2L2 ) ) / ( ( 0/2 * ( H3N4 + H3N4F1 + H3N5 + H3N4F2 + H3N5F1 ) + 1/2 * ( H4N4 + H4N4F1 + H4N5 + H4N4L1 + H4N4E1 + H4N5F1 + H4N4F1E1 + H4N5E1 + H4N4E1L1 + H4N5F1E1 + H4N4F1E2 + H4N4F2L2 + H4N4F2E1L1 + H4N5F2L2 ) + 2/2 * ( H5N4 + H5N4F1 + H5N5 + H5N4L1 + H5N4E1 + H5N5F1 + H5N4F1L1 + H4N4E1L1 + H5N4F1E1 + H3N4F2L2 + H5N5E1 + H5N4E1L1 + H4N4F1E2 + H5N4E2 + H5N5F1E1 + H4N4F2L2 + H5N4F1L2 + H4N4F2E1L1 + H5N4F1E1L1 + H5N4F1E2 + H5N5E2 + H5N5F1E2 + H5N5F2L2 ) ) / ( H3N4 + H3N4F1 + H4N4 + H3N5 + H3N4F2 + H4N4F1 + H5N4 + H3N5F1 + H4N5 + H4N4L1 + H3N4F1E1 + H5N4F1 + H4N4E1 + H4N5F1 + H5N5 + H5N4L1 + H4N4F1E1 + H5N4E1 + H5N5F1 + H4N5E1 + H5N4F1L1 + H4N4E1L1 + H5N4F1E1 + H4N5F1E1 + H3N4F2L2 + H5N5E1 + H5N4E1L1 + H3N5F1E1L1 + H4N4F1E2 + H5N4E2 + H5N5F1E1 + H4N4F2L2 + H5N4F1L2 + H4N4F2E1L1 + H5N4F1E1L1 + H5N4F1E2 + H5N5E2 + H4N5F2L2 + H5N5F1E2 + H5N5F2L2 ) ) |
| **A2FGS** | 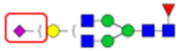 | Sialylation per galactose within fucosylated diantennary glycans | A2FGS = ( ( 0/2 * ( H3N4F1 + H3N4F2 + H4N4F1 + H3N5F1 + H5N4F1 + H4N5F1 + H5N5F1 ) + 1/2 * ( H3N4F1E1 + H4N4F1E1 + H5N4F1L1 + H5N4F1E1 + H4N5F1E1 + H5N5F1E1 ) + 2/2 * ( H3N4F2L2 + H3N5F1E1L1 + H4N4F1E2 + H4N4F2L2 + H5N4F1L2 + H4N4F2E1L1 + H5N4F1E1L1 + H5N4F1E2 + H4N5F2L2 + H5N5F1E2 + H5N5F2L2 ) ) / ( H3N4F1 + H3N4F2 + H4N4F1 + H3N5F1 + H3N4F1E1 + H5N4F1 + H4N5F1 + H4N4F1E1 + H5N5F1 + H5N4F1L1 + H5N4F1E1 + H4N5F1E1 + H3N4F2L2 + H3N5F1E1L1 + H4N4F1E2 + H5N5F1E1 + H4N4F2L2 + H5N4F1L2 + H4N4F2E1L1 + H5N4F1E1L1 + H5N4F1E2 + H4N5F2L2 + H5N5F1E2 + H5N5F2L2 ) ) / ( ( 0/2 * ( H3N4F1 + H3N4F2 + H3N5F1 ) + 1/2 * ( H4N4F1 + H4N5F1 + H4N4F1E1 + H4N5F1E1 + H4N4F1E2 + H4N4F2L2 + H4N4F2E1L1 + H4N5F2L2 ) + 2/2 * ( H5N4F1 + H5N5F1 + H5N4F1L1 + H5N4F1E1 + H3N4F2L2 + H4N4F1E2 + H5N5F1E1 + H4N4F2L2 + H5N4F1L2 + H4N4F2E1L1 + H5N4F1E1L1 + H5N4F1E2 + H5N5F1E2 + H5N5F2L2 ) ) / ( H3N4F1 + H3N4F2 + H4N4F1 + H3N5F1 + H3N4F1E1 + H5N4F1 + H4N5F1 + H4N4F1E1 + H5N5F1 + H5N4F1L1 + H5N4F1E1 + H4N5F1E1 + H3N4F2L2 + H3N5F1E1L1 + H4N4F1E2 + H5N5F1E1 + H4N4F2L2 + H5N4F1L2 + H4N4F2E1L1 + H5N4F1E1L1 + H5N4F1E2 + H4N5F2L2 + H5N5F1E2 + H5N5F2L2 ) ) |
| **A2F0GS** | 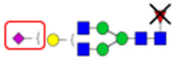 | Sialylation per galactose within non-fucosylated diantennary glycans | A2F0GS = ( ( 0/2 * ( H3N4 + H4N4 + H3N5 + H5N4 + H4N5 + H5N5 ) + 1/2 * ( H4N4L1 + H4N4E1 + H5N4L1 + H5N4E1 + H4N5E1 + H5N5E1 ) + 2/2 * ( H4N4E1L1 + H5N4E1L1 + H5N4E2 + H5N5E2 ) ) / ( H3N4 + H4N4 + H3N5 + H5N4 + H4N5 + H4N4L1 + H4N4E1 + H5N5 + H5N4L1 + H5N4E1 + H4N5E1 + H4N4E1L1 + H5N5E1 + H5N4E1L1 + H5N4E2 + H5N5E2 ) ) / ( ( 0/2 * ( H3N4 + H3N5 ) + 1/2 * ( H4N4 + H4N5 + H4N4L1 + H4N4E1 + H4N5E1 + H4N4E1L1 ) + 2/2 * ( H5N4 + H5N5 + H5N4L1 + H5N4E1 + H4N4E1L1 + H5N5E1 + H5N4E1L1 + H5N4E2 + H5N5E2 ) ) / ( H3N4 + H4N4 + H3N5 + H5N4 + H4N5 + H4N4L1 + H4N4E1 + H5N5 + H5N4L1 + H5N4E1 + H4N5E1 + H4N4E1L1 + H5N5E1 + H5N4E1L1 + H5N4E2 + H5N5E2 ) ) |
| **A4FGS** | 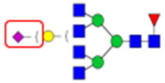 | Sialylation per galactose within fucosylated tetra-antennary glycans | A4FGS = ( ( 0/4 * (0) + 1/4 * ( H4N6F1E1 ) + 2/4 * (0) + 3/4 * ( H7N6F1E1L2 ) + 4/4 * ( H7N6F1E1L3 + H7N6F1E2L2 ) ) / ( H4N6F1E1 + H7N6E1 + H7N6E2 + H7N6E1L2 + H7N6E2L1 + H7N6E3 + H7N6F1E1L2 + H7N6E1L3 + H7N6E2L2 + H7N6E3L1 + H7N6F1E1L3 + H7N6F1E2L2 ) ) / ( ( 0/4 * (0) + 1/4 * ( H4N6F1E1 ) + 2/4 * (0) + 3/4 * (0) + 4/4 * ( H7N6F1E1L2 + H7N6F1E1L3 + H7N6F1E2L2 ) ) / ( H4N6F1E1 + H7N6E1 + H7N6E2 + H7N6E1L2 + H7N6E2L1 + H7N6E3 + H7N6F1E1L2 + H7N6E1L3 + H7N6E2L2 + H7N6E3L1 + H7N6F1E1L3 + H7N6F1E2L2 ) ) |

| | | | |
|---|---|---|---|
| **A4F0GS** | 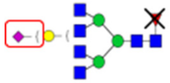 | Sialylation per galactose within non-fucosylated tetra-antennary glycans | A4F0GS = ( ( 0/4 * (0) + 1/4 * ( H7N6E1 ) + 2/4 * ( H7N6E2 ) + 3/4 * ( H7N6E1L2 + H7N6E2L1 + H7N6E3 ) + 4/4 * ( H7N6E1L3 + H7N6E2L2 + H7N6E3L1 ) ) / ( H4N6F1E1 + H7N6E1 + H7N6E2 + H7N6E1L2 + H7N6E2L1 + H7N6E3 + H7N6F1E1L2 + H7N6E1L3 + H7N6E2L2 + H7N6E3L1 + H7N6F1E1L3 + H7N6F1E2L2 ) ) / ( ( 0/4 * (0) + 1/4 * (0) + 2/4 * (0) + 3/4 * (0) + 4/4 * ( H7N6E1 + H7N6E2 + H7N6E1L2 + H7N6E2L1 + H7N6E3 + H7N6E1L3 + H7N6E2L2 + H7N6E3L1 ) ) / ( H4N6F1E1 + H7N6E1 + H7N6E2 + H7N6E1L2 + H7N6E2L1 + H7N6E3 + H7N6F1E1L2 + H7N6E1L3 + H7N6E2L2 + H7N6E3L1 + H7N6F1E1L3 + H7N6F1E2L2 ) ) |
| | | **α2,3-linked sialylation (L)** | |
| **A2L** | 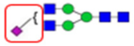 | α2,3-sialylation per antenna within diantennary glycans | A2L = ( 0/2 * ( H3N4 + H3N4F1 + H4N4 + H3N5 + H3N4F2 + H4N4F1 + H5N4 + H3N5F1 + H4N5 + H3N4F1E1 + H5N4F1 + H4N4E1 + H4N5F1 + H5N5 + H4N4F1E1 + H5N4E1 + H5N5F1 + H4N5E1 + H5N4F1E1 + H4N5F1E1 + H5N5E1 + H4N4F1E2 + H5N4E2 + H5N5F1E1 + H5N4F1E2 + H5N5E2 + H5N5F1E2 ) + 1/2 * ( H4N4L1 + H5N4L1 + H5N4F1L1 + H4N4E1L1 + H5N4E1L1 + H3N5F1E1L1 + H4N4F2E1L1 + H5N4F1E1L1 ) + 2/2 * ( H3N4F2L2 + H4N4F2L2 + H5N4F1L2 + H4N5F2L2 + H5N5F2L2 ) ) / ( H3N4 + H3N4F1 + H4N4 + H3N5 + H3N4F2 + H4N4F1 + H5N4 + H3N5F1 + H4N5 + H4N4L1 + H3N4F1E1 + H5N4F1 + H4N4E1 + H4N5F1 + H5N5 + H5N4L1 + H4N4F1E1 + H5N4E1 + H5N5F1 + H4N5E1 + H5N4F1L1 + H4N4E1L1 + H5N4F1E1 + H4N5F1E1 + H3N4F2L2 + H5N5E1 + H5N4E1L1 + H3N5F1E1L1 + H4N4F1E2 + H5N4E2 + H5N5F1E1 + H4N4F2L2 + H5N4F1L2 + H4N4F2E1L1 + H5N4F1E1L1 + H5N4F1E2 + H5N5E2 + H4N5F2L2 + H5N5F1E2 + H5N5F2L2 ) |
| **A2FL** | 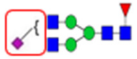 | α2,3-sialylation per antenna within fucosylated diantennary glycans | A2FL = ( 0/2 * ( H3N4F1 + H3N4F2 + H4N4F1 + H3N5F1 + H3N4F1E1 + H5N4F1 + H4N5F1 + H4N4F1E1 + H5N5F1 + H5N4F1E1 + H4N5F1E1 + H4N4F1E2 + H5N5F1E1 + H5N4F1E2 + H5N5F1E2 ) + 1/2 * ( H5N4F1L1 + H3N5F1E1L1 + H4N4F2E1L1 + H5N4F1E1L1 ) + 2/2 * ( H3N4F2L2 + H4N4F2L2 + H5N4F1L2 + H4N5F2L2 + H5N5F2L2 ) ) / ( H3N4F1 + H3N4F2 + H4N4F1 + H3N5F1 + H3N4F1E1 + H5N4F1 + H4N5F1 + H4N4F1E1 + H5N5F1 + H5N4F1L1 + H5N4F1E1 + H4N5F1E1 + H3N4F2L2 + H3N5F1E1L1 + H4N4F1E2 + H5N5F1E1 + H4N4F2L2 + H5N4F1L2 + H4N4F2E1L1 + H5N4F1E1L1 + H5N4F1E2 + H4N5F2L2 + H5N5F1E2 + H5N5F2L2 ) |
| **A2F0L** | 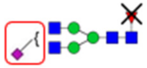 | α2,3-sialylation per antenna within non-fucosylated diantennary glycans | A2F0L = ( 0/2 * ( H3N4 + H4N4 + H3N5 + H5N4 + H4N5 + H4N4E1 + H5N5 + H5N4E1 + H4N5E1 + H5N5E1 + H5N4E2 + H5N5E2 ) + 1/2 * ( H4N4L1 + H5N4L1 + H4N4E1L1 + H5N4E1L1 ) + 2/2 * (0) ) / ( H3N4 + H4N4 + H3N5 + H5N4 + H4N5 + H4N4L1 + H4N4E1 + H5N5 + H5N4L1 + H5N4E1 + H4N5E1 + H4N4E1L1 + H5N5E1 + H5N4E1L1 + H5N4E2 + H5N5E2 ) |
| **A3L** | 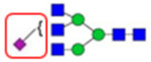 | α2,3-sialylation per antenna within triantennary glycans | A3L = ( 0/3 * ( H6N5E1 + H6N5F1E1 + H6N5E2 + H6N5F1E2 + H6N5E3 + H6N5F1E3 ) + 1/3 * ( H6N5E1L1 + H6N5F1E1L1 + H6N5E2L1 + H6N5F1E2L1 + H6N5F2E2L1 ) + 2/3 * ( H6N5F1L2 + H6N5E1L2 + H6N5F1E1L2 ) + 3/3 * ( H6N5F1L3 ) ) / ( H6N5E1 + H6N5F1E1 + H6N5E1L1 + H6N5E2 + H6N5F1L2 + H6N5F1E1L1 + H6N5F1E2 + H6N5E1L2 + H6N5E2L1 + H6N5E3 + H6N5F1L3 + H6N5F1E1L2 + H6N5F1E2L1 + H6N5F1E3 + H6N5F2E2L1 ) |
| **A3FL** | 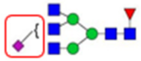 | α2,3-sialylation per antenna within fucosylated triantennary glycans | A3FL = ( 0/3 * ( H6N5F1E1 + H6N5F1E2 + H6N5F1E3 ) + 1/3 * ( H6N5F1E1L1 + H6N5F1E2L1 + H6N5F2E2L1 ) + 2/3 * ( H6N5F1L2 + H6N5F1E1L2 ) + 3/3 * ( H6N5F1L3 ) ) / ( H6N5F1E1 + H6N5F1L2 + H6N5F1E1L1 + H6N5F1E2 + H6N5F1L3 + H6N5F1E1L2 + H6N5F1E2L1 + H6N5F1E3 + H6N5F2E2L1 ) |
| **A3F0L** | 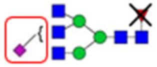 | α2,3-sialylation per antenna within non-fucosylated triantennary glycans | A3F0L = ( 0/3 * ( H6N5E1 + H6N5E2 + H6N5E3 ) + 1/3 * ( H6N5E1L1 + H6N5E2L1 ) + 2/3 * ( H6N5E1L2 ) + 3/3 * (0) ) / ( H6N5E1 + H6N5E1L1 + H6N5E2 + H6N5E1L2 + H6N5E2L1 + H6N5E3 ) |
| **A4L** | 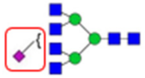 | α2,3-sialylation per antenna within tetra-antennary glycans | A4L = ( 0/4 * ( H4N6F1E1 + H7N6E1 + H7N6E2 + H7N6E3 ) + 1/4 * ( H7N6E2L1 + H7N6E3L1 ) + 2/4 * ( H7N6E1L2 + H7N6F1E1L2 + H7N6E2L2 + H7N6F1E2L2 ) + 3/4 * ( H7N6E1L3 + H7N6F1E1L3 ) + 4/4 * (0) ) / ( H4N6F1E1 + H7N6E1 + H7N6E2 + H7N6E1L2 + H7N6E2L1 + H7N6E3 + H7N6F1E1L2 + H7N6E1L3 + H7N6E2L2 + H7N6E3L1 + H7N6F1E1L3 + H7N6F1E2L2 ) |

| | | | |
|---|---|---|---|
| **A4FL** | 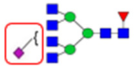 | α2,3-sialylation per antenna within fucosylated tetra-antennary glycans | A4FL = ( 0/4 * ( H4N6F1E1 ) + 1/4 * (0) + 2/4 * ( H7N6F1E1L2 + H7N6F1E2L2 ) + 3/4 * ( H7N6F1E1L3 ) + 4/4 * (0) ) / ( H4N6F1E1 + H7N6E1 + H7N6E2 + H7N6E1L2 + H7N6E2L1 + H7N6E3 + H7N6F1E1L2 + H7N6E1L3 + H7N6E2L2 + H7N6E3L1 + H7N6F1E1L3 + H7N6F1E2L2 ) |
| **A4F0L** | 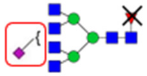 | α2,3-sialylation per antenna within non-fucosylated tetra-antennary glycans | A4F0L = ( 0/4 * ( H7N6E1 + H7N6E2 + H7N6E3 ) + 1/4 * ( H7N6E2L1 + H7N6E3L1 ) + 2/4 * ( H7N6E1L2 + H7N6E2L2 ) + 3/4 * ( H7N6E1L3 ) + 4/4 * (0) ) / ( H4N6F1E1 + H7N6E1 + H7N6E2 + H7N6E1L2 + H7N6E2L1 + H7N6E3 + H7N6F1E1L2 + H7N6E1L3 + H7N6E2L2 + H7N6E3L1 + H7N6F1E1L3 + H7N6F1E2L2 ) |
| **A2GL** | 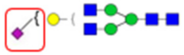 | α2,3-sialylation per galactose within diantennary glycans | A2GL = ( ( 0/2 * ( H3N4 + H3N4F1 + H4N4 + H3N5 + H3N4F2 + H4N4F1 + H5N4 + H3N5F1 + H4N5 + H3N4F1E1 + H5N4F1 + H4N4E1 + H4N5F1 + H5N5 + H4N4F1E1 + H5N4E1 + H5N5F1 + H4N5E1 + H5N4F1E1 + H4N5F1E1 + H5N5E1 + H4N4F1E2 + H5N4E2 + H5N5F1E1 + H5N4F1E2 + H5N5E2 + H5N5F1E2 ) + 1/2 * ( H4N4L1 + H5N4L1 + H5N4F1L1 + H4N4E1L1 + H5N4E1L1 + H3N5F1E1L1 + H4N4F2E1L1 + H5N4F1E1L1 ) + 2/2 * ( H3N4F2L2 + H4N4F2L2 + H5N4F1L2 + H4N5F2L2 + H5N5F2L2 ) ) / ( H3N4 + H3N4F1 + H4N4 + H3N5 + H3N4F2 + H4N4F1 + H5N4 + H3N5F1 + H4N5 + H4N4L1 + H3N4F1E1 + H5N4F1 + H4N4E1 + H4N5F1 + H5N5 + H5N4L1 + H4N4F1E1 + H5N4E1 + H5N5F1 + H4N5E1 + H5N4F1L1 + H4N4E1L1 + H5N4F1E1 + H4N5F1E1 + H3N4F2L2 + H5N5E1 + H5N4E1L1 + H3N5F1E1L1 + H4N4F1E2 + H5N4E2 + H5N5F1E1 + H4N4F2L2 + H5N4F1L2 + H4N4F2E1L1 + H5N4F1E1L1 + H5N4F1E2 + H5N5E2 + H4N5F2L2 + H5N5F1E2 + H5N5F2L2 ) ) / ( ( 0/2 * ( H3N4 + H3N4F1 + H3N5 + H3N4F2 + H3N5F1 ) + 1/2 * ( H4N4 + H4N4F1 + H4N5 + H4N4L1 + H4N4E1 + H4N5F1 + H4N4F1E1 + H4N5E1 + H4N4E1L1 + H4N5F1E1 + H4N4F1E2 + H4N4F2L2 + H4N4F2E1L1 + H4N5F2L2 ) + 2/2 * ( H5N4 + H5N4F1 + H5N5 + H5N4L1 + H5N4E1 + H5N5F1 + H5N4F1L1 + H4N4E1L1 + H5N4F1E1 + H3N4F2L2 + H5N5E1 + H5N4E1L1 + H4N4F1E2 + H5N4E2 + H5N5F1E1 + H4N4F2L2 + H5N4F1L2 + H4N4F2E1L1 + H5N4F1E1L1 + H5N4F1E2 + H5N5E2 + H5N5F1E2 + H5N5F2L2 ) ) / ( H3N4 + H3N4F1 + H4N4 + H3N5 + H3N4F2 + H4N4F1 + H5N4 + H3N5F1 + H4N5 + H4N4L1 + H3N4F1E1 + H5N4F1 + H4N4E1 + H4N5F1 + H5N5 + H5N4L1 + H4N4F1E1 + H5N4E1 + H5N5F1 + H4N5E1 + H5N4F1L1 + H4N4E1L1 + H5N4F1E1 + H4N5F1E1 + H3N4F2L2 + H5N5E1 + H5N4E1L1 + H3N5F1E1L1 + H4N4F1E2 + H5N4E2 + H5N5F1E1 + H4N4F2L2 + H5N4F1L2 + H4N4F2E1L1 + H5N4F1E1L1 + H5N4F1E2 + H5N5E2 + H4N5F2L2 + H5N5F1E2 + H5N5F2L2 ) ) |
| **A2FGL** | 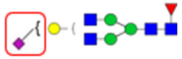 | α2,3-sialylation per galactose within fucosylated diantennary glycans | A2FGL = ( ( 0/2 * ( H3N4F1 + H3N4F2 + H4N4F1 + H3N5F1 + H3N4F1E1 + H5N4F1 + H4N5F1 + H4N4F1E1 + H5N5F1 + H5N4F1E1 + H4N5F1E1 + H4N4F1E2 + H5N5F1E1 + H5N4F1E2 + H5N5F1E2 ) + 1/2 * ( H5N4F1L1 + H3N5F1E1L1 + H4N4F2E1L1 + H5N4F1E1L1 ) + 2/2 * ( H3N4F2L2 + H4N4F2L2 + H5N4F1L2 + H4N5F2L2 + H5N5F2L2 ) ) / ( H3N4F1 + H3N4F2 + H4N4F1 + H3N5F1 + H3N4F1E1 + H5N4F1 + H4N5F1 + H4N4F1E1 + H5N5F1 + H5N4F1L1 + H5N4F1E1 + H4N5F1E1 + H3N4F2L2 + H3N5F1E1L1 + H4N4F1E2 + H5N5F1E1 + H4N4F2L2 + H5N4F1L2 + H4N4F2E1L1 + H5N4F1E1L1 + H5N4F1E2 + H4N5F2L2 + H5N5F1E2 + H5N5F2L2 ) ) / ( ( 0/2 * ( H3N4F1 + H3N4F2 + H3N5F1 ) + 1/2 * ( H4N4F1 + H4N5F1 + H4N4F1E1 + H4N5F1E1 + H4N4F1E2 + H4N4F2L2 + H4N4F2E1L1 + H4N5F2L2 ) + 2/2 * ( H5N4F1 + H5N5F1 + H5N4F1L1 + H5N4F1E1 + H3N4F2L2 + H4N4F1E2 + H5N5F1E1 + H4N4F2L2 + H5N4F1L2 + H4N4F2E1L1 + H5N4F1E1L1 + H5N4F1E2 + H5N5F1E2 + H5N5F2L2 ) ) / ( H3N4F1 + H3N4F2 + H4N4F1 + H3N5F1 + H3N4F1E1 + H5N4F1 + H4N5F1 + H4N4F1E1 + H5N5F1 + H5N4F1L1 + H5N4F1E1 + H4N5F1E1 + H3N4F2L2 + H3N5F1E1L1 + H4N4F1E2 + H5N5F1E1 + H4N4F2L2 + H5N4F1L2 + H4N4F2E1L1 + H5N4F1E1L1 + H5N4F1E2 + H4N5F2L2 + H5N5F1E2 + H5N5F2L2 ) ) |
| **A2F0GL** | 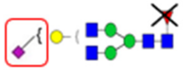 | α2,3-sialylation per galactose within non-fucosylated diantennary glycans | A2F0GL = ( ( 0/2 * ( H3N4 + H4N4 + H3N5 + H5N4 + H4N5 + H4N4E1 + H5N5 + H5N4E1 + H4N5E1 + H5N5E1 + H5N4E2 + H5N5E2 ) + 1/2 * ( H4N4L1 + H5N4L1 + H4N4E1L1 + H5N4E1L1 ) + 2/2 * (0) ) / ( H3N4 + H4N4 + H3N5 + H5N4 + H4N5 + H4N4L1 + H4N4E1 + H5N5 + H5N4L1 + H5N4E1 + H4N5E1 + H4N4E1L1 + H5N5E1 + H5N4E1L1 + H5N4E2 + H5N5E2 ) ) / ( ( 0/2 * ( H3N4 + H3N5 ) + 1/2 * ( H4N4 + H4N5 + H4N4L1 + H4N4E1 + H4N5E1 + H4N4E1L1 ) + 2/2 * ( H5N4 + H5N5 + H5N4L1 + H5N4E1 + H4N4E1L1 + H5N5E1 + H5N4E1L1 + H5N4E2 + H5N5E2 ) ) / ( H3N4 + H4N4 + H3N5 + H5N4 + H4N5 + H4N4L1 + H4N4E1 + H5N5 + H5N4L1 + H5N4E1 + H4N5E1 + H4N4E1L1 + H5N5E1 + H5N4E1L1 + H5N4E2 + H5N5E2 ) ) |

| | | | |
|---|---|---|---|
| **A4FGL** | 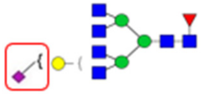 | α2,3-sialylation per galactose within fucosylated tetra-antennary glycans | A4FGL = ( ( 0/4 * ( H4N6F1E1 ) + 1/4 * (0) + 2/4 * ( H7N6F1E1L2 + H7N6F1E2L2 ) + 3/4 * ( H7N6F1E1L3 ) + 4/4 * (0) ) / ( H4N6F1E1 + H7N6E1 + H7N6E2 + H7N6E1L2 + H7N6E2L1 + H7N6E3 + H7N6F1E1L2 + H7N6E1L3 + H7N6E2L2 + H7N6E3L1 + H7N6F1E1L3 + H7N6F1E2L2 ) ) / ( ( 0/4 * (0) + 1/4 * ( H4N6F1E1 ) + 2/4 * (0) + 3/4 * (0) + 4/4 * ( H7N6F1E1L2 + H7N6F1E1L3 + H7N6F1E2L2 ) ) / ( H4N6F1E1 + H7N6E1 + H7N6E2 + H7N6E1L2 + H7N6E2L1 + H7N6E3 + H7N6F1E1L2 + H7N6E1L3 + H7N6E2L2 + H7N6E3L1 + H7N6F1E1L3 + H7N6F1E2L2 ) ) |
| **A4F0GL** | 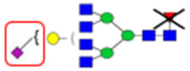 | α2,3-sialylation per galactose within non-fucosylated tetra-antennary glycans | A4F0GL = ( ( 0/4 * ( H7N6E1 + H7N6E2 + H7N6E3 ) + 1/4 * ( H7N6E2L1 + H7N6E3L1 ) + 2/4 * ( H7N6E1L2 + H7N6E2L2 ) + 3/4 * ( H7N6E1L3 ) + 4/4 * (0) ) / ( H4N6F1E1 + H7N6E1 + H7N6E2 + H7N6E1L2 + H7N6E2L1 + H7N6E3 + H7N6F1E1L2 + H7N6E1L3 + H7N6E2L2 + H7N6E3L1 + H7N6F1E1L3 + H7N6F1E2L2 ) ) / ( ( 0/4 * (0) + 1/4 * (0) + 2/4 * (0) + 3/4 * (0) + 4/4 * ( H7N6E1 + H7N6E2 + H7N6E1L2 + H7N6E2L1 + H7N6E3 + H7N6E1L3 + H7N6E2L2 + H7N6E3L1 ) ) / ( H4N6F1E1 + H7N6E1 + H7N6E2 + H7N6E1L2 + H7N6E2L1 + H7N6E3 + H7N6F1E1L2 + H7N6E1L3 + H7N6E2L2 + H7N6E3L1 + H7N6F1E1L3 + H7N6F1E2L2 ) ) |
| | | **α2,6-linked sialylation (E)** | |
| **A2E** | 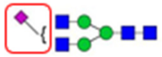 | α2,6-sialylation per antenna within diantennary glycans | A2E = ( 0/2 * ( H3N4 + H3N4F1 + H4N4 + H3N5 + H3N4F2 + H4N4F1 + H5N4 + H3N5F1 + H4N5 + H4N4L1 + H5N4F1 + H4N5F1 + H5N5 + H5N4L1 + H5N5F1 + H5N4F1L1 + H3N4F2L2 + H4N4F2L2 + H5N4F1L2 + H4N5F2L2 + H5N5F2L2 ) + 1/2 * ( H3N4F1E1 + H4N4E1 + H4N4F1E1 + H5N4E1 + H4N5E1 + H4N4E1L1 + H5N4F1E1 + H4N5F1E1 + H5N5E1 + H5N4E1L1 + H3N5F1E1L1 + H5N5F1E1 + H4N4F2E1L1 + H5N4F1E1L1 ) + 2/2 * ( H4N4F1E2 + H5N4E2 + H5N4F1E2 + H5N5E2 + H5N5F1E2 ) ) / ( H3N4 + H3N4F1 + H4N4 + H3N5 + H3N4F2 + H4N4F1 + H5N4 + H3N5F1 + H4N5 + H4N4L1 + H3N4F1E1 + H5N4F1 + H4N4E1 + H4N5F1 + H5N5 + H5N4L1 + H4N4F1E1 + H5N4E1 + H5N5F1 + H4N5E1 + H5N4F1L1 + H4N4E1L1 + H5N4F1E1 + H4N5F1E1 + H3N4F2L2 + H5N5E1 + H5N4E1L1 + H3N5F1E1L1 + H4N4F1E2 + H5N4E2 + H5N5F1E1 + H4N4F2L2 + H5N4F1L2 + H4N4F2E1L1 + H5N4F1E1L1 + H5N4F1E2 + H5N5E2 + H4N5F2L2 + H5N5F1E2 + H5N5F2L2 ) |
| **A2FE** | 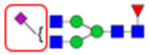 | α2,6-sialylation per antenna within fucosylated diantennary glycans | A2FE = ( 0/2 * ( H3N4F1 + H3N4F2 + H4N4F1 + H3N5F1 + H5N4F1 + H4N5F1 + H5N5F1 + H5N4F1L1 + H3N4F2L2 + H4N4F2L2 + H5N4F1L2 + H4N5F2L2 + H5N5F2L2 ) + 1/2 * ( H3N4F1E1 + H4N4F1E1 + H5N4F1E1 + H4N5F1E1 + H3N5F1E1L1 + H5N5F1E1 + H4N4F2E1L1 + H5N4F1E1L1 ) + 2/2 * ( H4N4F1E2 + H5N4F1E2 + H5N5F1E2 ) ) / ( H3N4F1 + H3N4F2 + H4N4F1 + H3N5F1 + H3N4F1E1 + H5N4F1 + H4N5F1 + H4N4F1E1 + H5N5F1 + H5N4F1L1 + H5N4F1E1 + H4N5F1E1 + H3N4F2L2 + H3N5F1E1L1 + H4N4F1E2 + H5N5F1E1 + H4N4F2L2 + H5N4F1L2 + H4N4F2E1L1 + H5N4F1E1L1 + H5N4F1E2 + H4N5F2L2 + H5N5F1E2 + H5N5F2L2 ) |
| **A2F0E** | 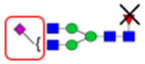 | α2,6-sialylation per antenna within non-fucosylated diantennary glycans | A2F0E = ( 0/2 * ( H3N4 + H4N4 + H3N5 + H5N4 + H4N5 + H4N4L1 + H5N5 + H5N4L1 ) + 1/2 * ( H4N4E1 + H5N4E1 + H4N5E1 + H4N4E1L1 + H5N5E1 + H5N4E1L1 ) + 2/2 * ( H5N4E2 + H5N5E2 ) ) / ( H3N4 + H4N4 + H3N5 + H5N4 + H4N5 + H4N4L1 + H4N4E1 + H5N5 + H5N4L1 + H5N4E1 + H4N5E1 + H4N4E1L1 + H5N5E1 + H5N4E1L1 + H5N4E2 + H5N5E2 ) |
| **A3E** | 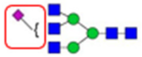 | α2,6-sialylation per antenna within triantennary glycans | A3E = ( 0/3 * ( H6N5F1L2 + H6N5F1L3 ) + 1/3 * ( H6N5E1 + H6N5F1E1 + H6N5E1L1 + H6N5F1E1L1 + H6N5E1L2 + H6N5F1E1L2 ) + 2/3 * ( H6N5E2 + H6N5F1E2 + H6N5E2L1 + H6N5F1E2L1 + H6N5F2E2L1 ) + 3/3 * ( H6N5E3 + H6N5F1E3 ) ) / ( H6N5E1 + H6N5F1E1 + H6N5E1L1 + H6N5E2 + H6N5F1L2 + H6N5F1E1L1 + H6N5F1E2 + H6N5E1L2 + H6N5E2L1 + H6N5E3 + H6N5F1L3 + H6N5F1E1L2 + H6N5F1E2L1 + H6N5F1E3 + H6N5F2E2L1 ) |
| **A3FE** | 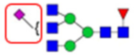 | α2,6-sialylation per antenna within fucosylated triantennary glycans | A3FE = ( 0/3 * ( H6N5F1L2 + H6N5F1L3 ) + 1/3 * ( H6N5F1E1 + H6N5F1E1L1 + H6N5F1E1L2 ) + 2/3 * ( H6N5F1E2 + H6N5F1E2L1 + H6N5F2E2L1 ) + 3/3 * ( H6N5F1E3 ) ) / ( H6N5F1E1 + H6N5F1L2 + H6N5F1E1L1 + H6N5F1E2 + H6N5F1L3 + H6N5F1E1L2 + H6N5F1E2L1 + H6N5F1E3 + H6N5F2E2L1 ) |
| **A3F0E** | 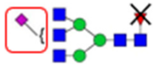 | α2,6-sialylation per antenna within non-fucosylated triantennary glycans | A3F0E = ( 0/3 * (0) + 1/3 * ( H6N5E1 + H6N5E1L1 + H6N5E1L2 ) + 2/3 * ( H6N5E2 + H6N5E2L1 ) + 3/3 * ( H6N5E3 ) ) / ( H6N5E1 + H6N5E1L1 + H6N5E2 + H6N5E1L2 + H6N5E2L1 + H6N5E3 ) |

| | | | |
|---|---|---|---|
| **A4E** | 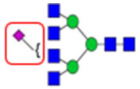 | α2,6-sialylation per antenna within tetra-antennary glycans | A4E = ( 0/4 * (0) + 1/4 * ( H4N6F1E1 + H7N6E1 + H7N6E1L2 + H7N6F1E1L2 + H7N6E1L3 + H7N6F1E1L3 ) + 2/4 * ( H7N6E2 + H7N6E2L1 + H7N6E2L2 + H7N6F1E2L2 ) + 3/4 * ( H7N6E3 + H7N6E3L1 ) + 4/4 * (0) ) / ( H4N6F1E1 + H7N6E1 + H7N6E2 + H7N6E1L2 + H7N6E2L1 + H7N6E3 + H7N6F1E1L2 + H7N6E1L3 + H7N6E2L2 + H7N6E3L1 + H7N6F1E1L3 + H7N6F1E2L2 ) |
| **A4FE** | 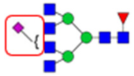 | α2,6-sialylation per antenna within fucosylated tetra-antennary glycans | A4FE = ( 0/4 * (0) + 1/4 * ( H4N6F1E1 + H7N6F1E1L2 + H7N6F1E1L3 ) + 2/4 * ( H7N6F1E2L2 ) + 3/4 * (0) + 4/4 * (0) ) / ( H4N6F1E1 + H7N6E1 + H7N6E2 + H7N6E1L2 + H7N6E2L1 + H7N6E3 + H7N6F1E1L2 + H7N6E1L3 + H7N6E2L2 + H7N6E3L1 + H7N6F1E1L3 + H7N6F1E2L2 ) |
| **A4F0E** | 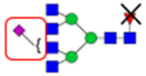 | α2,6-sialylation per antenna within non-fucosylated tetra-antennary glycans | A4F0E = ( 0/4 * (0) + 1/4 * ( H7N6E1 + H7N6E1L2 + H7N6E1L3 ) + 2/4 * ( H7N6E2 + H7N6E2L1 + H7N6E2L2 ) + 3/4 * ( H7N6E3 + H7N6E3L1 ) + 4/4 * (0) ) / ( H4N6F1E1 + H7N6E1 + H7N6E2 + H7N6E1L2 + H7N6E2L1 + H7N6E3 + H7N6F1E1L2 + H7N6E1L3 + H7N6E2L2 + H7N6E3L1 + H7N6F1E1L3 + H7N6F1E2L2 ) |
| **A2GE** | 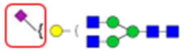 | α2,6-sialylation per galactose within diantennary glycans | A2GE = ( ( 0/2 * ( H3N4 + H3N4F1 + H4N4 + H3N5 + H3N4F2 + H4N4F1 + H5N4 + H3N5F1 + H4N5 + H4N4L1 + H5N4F1 + H4N5F1 + H5N5 + H5N4L1 + H5N5F1 + H5N4F1L1 + H3N4F2L2 + H4N4F2L2 + H5N4F1L2 + H4N5F2L2 + H5N5F2L2 ) + 1/2 * ( H3N4F1E1 + H4N4E1 + H4N4F1E1 + H5N4E1 + H4N5E1 + H4N4E1L1 + H5N4F1E1 + H4N5F1E1 + H5N5E1 + H5N4E1L1 + H3N5F1E1L1 + H5N5F1E1 + H4N4F2E1L1 + H5N4F1E1L1 ) + 2/2 * ( H4N4F1E2 + H5N4E2 + H5N4F1E2 + H5N5E2 + H5N5F1E2 ) ) / ( H3N4 + H3N4F1 + H4N4 + H3N5 + H3N4F2 + H4N4F1 + H5N4 + H3N5F1 + H4N5 + H4N4L1 + H3N4F1E1 + H5N4F1 + H4N4E1 + H4N5F1 + H5N5 + H5N4L1 + H4N4F1E1 + H5N4E1 + H5N5F1 + H4N5E1 + H5N4F1L1 + H4N4E1L1 + H5N4F1E1 + H4N5F1E1 + H3N4F2L2 + H5N5E1 + H5N4E1L1 + H3N5F1E1L1 + H4N4F1E2 + H5N4E2 + H5N5F1E1 + H4N4F2L2 + H5N4F1L2 + H4N4F2E1L1 + H5N4F1E1L1 + H5N4F1E2 + H5N5E2 + H4N5F2L2 + H5N5F1E2 + H5N5F2L2 ) ) / ( ( 0/2 * ( H3N4 + H3N4F1 + H3N5 + H3N4F2 + H3N5F1 ) + 1/2 * ( H4N4 + H4N4F1 + H4N5 + H4N4L1 + H4N4E1 + H4N5F1 + H4N4F1E1 + H4N5E1 + H4N4E1L1 + H4N5F1E1 + H4N4F1E2 + H4N4F2L2 + H4N4F2E1L1 + H4N5F2L2 ) + 2/2 * ( H5N4 + H5N4F1 + H5N5 + H5N4L1 + H5N4E1 + H5N5F1 + H5N4F1L1 + H4N4E1L1 + H5N4F1E1 + H3N4F2L2 + H5N5E1 + H5N4E1L1 + H4N4F1E2 + H5N4E2 + H5N5F1E1 + H4N4F2L2 + H5N4F1L2 + H4N4F2E1L1 + H5N4F1E1L1 + H5N4F1E2 + H5N5E2 + H5N5F1E2 + H5N5F2L2 ) ) / ( H3N4 + H3N4F1 + H4N4 + H3N5 + H3N4F2 + H4N4F1 + H5N4 + H3N5F1 + H4N5 + H4N4L1 + H3N4F1E1 + H5N4F1 + H4N4E1 + H4N5F1 + H5N5 + H5N4L1 + H4N4F1E1 + H5N4E1 + H5N5F1 + H4N5E1 + H5N4F1L1 + H4N4E1L1 + H5N4F1E1 + H4N5F1E1 + H3N4F2L2 + H5N5E1 + H5N4E1L1 + H3N5F1E1L1 + H4N4F1E2 + H5N4E2 + H5N5F1E1 + H4N4F2L2 + H5N4F1L2 + H4N4F2E1L1 + H5N4F1E1L1 + H5N4F1E2 + H5N5E2 + H4N5F2L2 + H5N5F1E2 + H5N5F2L2 ) ) |
| **A2FGE** | 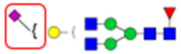 | α2,6-sialylation per galactose within fucosylated diantennary glycans | A2FGE = ( ( 0/2 * ( H3N4F1 + H3N4F2 + H4N4F1 + H3N5F1 + H5N4F1 + H4N5F1 + H5N5F1 + H5N4F1L1 + H3N4F2L2 + H4N4F2L2 + H5N4F1L2 + H4N5F2L2 + H5N5F2L2 ) + 1/2 * ( H3N4F1E1 + H4N4F1E1 + H5N4F1E1 + H4N5F1E1 + H3N5F1E1L1 + H5N5F1E1 + H4N4F2E1L1 + H5N4F1E1L1 ) + 2/2 * ( H4N4F1E2 + H5N4F1E2 + H5N5F1E2 ) ) / ( H3N4F1 + H3N4F2 + H4N4F1 + H3N5F1 + H3N4F1E1 + H5N4F1 + H4N5F1 + H4N4F1E1 + H5N5F1 + H5N4F1L1 + H5N4F1E1 + H4N5F1E1 + H3N4F2L2 + H3N5F1E1L1 + H4N4F1E2 + H5N5F1E1 + H4N4F2L2 + H5N4F1L2 + H4N4F2E1L1 + H5N4F1E1L1 + H5N4F1E2 + H4N5F2L2 + H5N5F1E2 + H5N5F2L2 ) ) / ( ( 0/2 * ( H3N4F1 + H3N4F2 + H3N5F1 ) + 1/2 * ( H4N4F1 + H4N5F1 + H4N4F1E1 + H4N5F1E1 + H4N4F1E2 + H4N4F2L2 + H4N4F2E1L1 + H4N5F2L2 ) + 2/2 * ( H5N4F1 + H5N5F1 + H5N4F1L1 + H5N4F1E1 + H3N4F2L2 + H4N4F1E2 + H5N5F1E1 + H4N4F2L2 + H5N4F1L2 + H4N4F2E1L1 + H5N4F1E1L1 + H5N4F1E2 + H5N5F1E2 + H5N5F2L2 ) ) / ( H3N4F1 + H3N4F2 + H4N4F1 + H3N5F1 + H3N4F1E1 + H5N4F1 + H4N5F1 + H4N4F1E1 + H5N5F1 + H5N4F1L1 + H5N4F1E1 + H4N5F1E1 + H3N4F2L2 + H3N5F1E1L1 + H4N4F1E2 + H5N5F1E1 + H4N4F2L2 + H5N4F1L2 + H4N4F2E1L1 + H5N4F1E1L1 + H5N4F1E2 + H4N5F2L2 + H5N5F1E2 + H5N5F2L2 ) ) |
| **A2F0GE** | 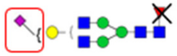 | α2,6-sialylation per galactose within non-fucosylated diantennary glycans | A2F0GE = ( ( 0/2 * ( H3N4 + H4N4 + H3N5 + H5N4 + H4N5 + H4N4L1 + H5N5 + H5N4L1 ) + 1/2 * ( H4N4E1 + H5N4E1 + H4N5E1 + H4N4E1L1 + H5N5E1 + H5N4E1L1 ) + 2/2 * ( H5N4E2 + H5N5E2 ) ) / ( H3N4 + H4N4 + H3N5 + H5N4 + H4N5 + H4N4L1 + H4N4E1 + H5N5 + H5N4L1 + H5N4E1 + H4N5E1 + H4N4E1L1 + H5N5E1 + H5N4E1L1 + H5N4E2 + H5N5E2 ) ) / ( ( 0/2 * ( H3N4 + H3N5 ) + 1/2 * ( H4N4 + H4N5 + H4N4L1 + H4N4E1 + H4N5E1 + H4N4E1L1 ) + 2/2 * ( H5N4 + H5N5 + H5N4L1 + H5N4E1 + H4N4E1L1 + H5N5E1 + H5N4E1L1 + H5N4E2 + H5N5E2 ) ) / ( H3N4 + H4N4 + H3N5 + H5N4 + H4N5 + H4N4L1 + H4N4E1 + H5N5 + H5N4L1 + H5N4E1 + H4N5E1 + H4N4E1L1 + H5N5E1 + H5N4E1L1 + H5N4E2 + H5N5E2 ) ) |

| | | | |
|---|---|---|---|
| **A4FGE** | 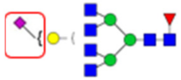 | α2,6-sialylation per galactose within fucosylated tetra-antennary glycans | A4FGE = ( ( 0/4 * (0) + 1/4 * ( H4N6F1E1 + H7N6F1E1L2 + H7N6F1E1L3 ) + 2/4 * ( H7N6F1E2L2 ) + 3/4 * (0) + 4/4 * (0) ) / ( H4N6F1E1 + H7N6E1 + H7N6E2 + H7N6E1L2 + H7N6E2L1 + H7N6E3 + H7N6F1E1L2 + H7N6E1L3 + H7N6E2L2 + H7N6E3L1 + H7N6F1E1L3 + H7N6F1E2L2 ) ) / ( ( 0/4 * (0) + 1/4 * ( H4N6F1E1 ) + 2/4 * (0) + 3/4 * (0) + 4/4 * ( H7N6F1E1L2 + H7N6F1E1L3 + H7N6F1E2L2 ) ) / ( H4N6F1E1 + H7N6E1 + H7N6E2 + H7N6E1L2 + H7N6E2L1 + H7N6E3 + H7N6F1E1L2 + H7N6E1L3 + H7N6E2L2 + H7N6E3L1 + H7N6F1E1L3 + H7N6F1E2L2 ) ) |
| **A4F0GE** | 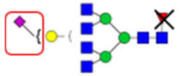 | α2,6-sialylation per galactose within non-fucosylated tetra-antennary glycans | A4F0GE = ( ( 0/4 * (0) + 1/4 * ( H7N6E1 + H7N6E1L2 + H7N6E1L3 ) + 2/4 * ( H7N6E2 + H7N6E2L1 + H7N6E2L2 ) + 3/4 * ( H7N6E3 + H7N6E3L1 ) + 4/4 * (0) ) / ( H4N6F1E1 + H7N6E1 + H7N6E2 + H7N6E1L2 + H7N6E2L1 + H7N6E3 + H7N6F1E1L2 + H7N6E1L3 + H7N6E2L2 + H7N6E3L1 + H7N6F1E1L3 + H7N6F1E2L2 ) ) / ( ( 0/4 * (0) + 1/4 * (0) + 2/4 * (0) + 3/4 * (0) + 4/4 * ( H7N6E1 + H7N6E2 + H7N6E1L2 + H7N6E2L1 + H7N6E3 + H7N6E1L3 + H7N6E2L2 + H7N6E3L1 ) ) / ( H4N6F1E1 + H7N6E1 + H7N6E2 + H7N6E1L2 + H7N6E2L1 + H7N6E3 + H7N6F1E1L2 + H7N6E1L3 + H7N6E2L2 + H7N6E3L1 + H7N6F1E1L3 + H7N6F1E2L2 ) ) |

**Table S3.** Data quality control. The data quality was checked by the measurement of a standard sample assessed by calculating the mean, SD (standard deviation) and CV (coefficient of variance) for all glycans.

| Directly detected glycan | Average (Relative abundance) | SD | RSD (CV) | Derived glycan | Average (Relative abundance) | SD | RSD (CV) |
|---|---|---|---|---|---|---|---|
| H3N3 | 0.039607833 | 0.01739846 | 0.439268152 | TM | 0.023171143 | 0.00257282 | 0.111035518 |
| H5N2 | 0.451708911 | 0.056776162 | 0.125691924 | THy | 0.012651728 | 0.00071093 | 0.05619236 |
| H3N3F1 | 0.07902041 | 0.017572557 | 0.222379977 | MHy | 1.835362385 | 0.193246285 | 0.105290534 |
| H4N3 | 0.110482431 | 0.037448074 | 0.338950487 | MM | 6.92791362 | 0.053928802 | 0.007784277 |
| H3N4 | 0.250042582 | 0.053446552 | 0.213749799 | CA1 | 0.009665167 | 0.000819922 | 0.084832639 |
| H6N2 | 0.728890248 | 0.084673577 | 0.1161678 | CA2 | 0.841862335 | 0.010030586 | 0.011914758 |
| H4N3F1 | 0.085699177 | 0.019654924 | 0.229347876 | CA3 | 0.118313529 | 0.006867529 | 0.058045171 |
| H3N3E1 | 0.153001481 | 0.022880891 | 0.149546862 | CA4 | 0.022235346 | 0.002589532 | 0.116460161 |
| H5N3 | 0.135301779 | 0.034397906 | 0.254230999 | CF | 0.402432253 | 0.008984035 | 0.022324341 |
| H3N4F1 | 2.64115728 | 0.272798686 | 0.103287558 | CFa | 0.010747781 | 0.000500058 | 0.046526598 |
| H4N4 | 0.348785435 | 0.035490834 | 0.101755494 | CB | 0.11385348 | 0.003424831 | 0.030081035 |
| H3N5 | 0.183147421 | 0.037997131 | 0.207467464 | CG | 0.957487837 | 0.004609285 | 0.004813936 |
| H7N2 | 0.210587231 | 0.033788086 | 0.160446983 | CS | 0.836809125 | 0.012313748 | 0.014715122 |
| H3N3F1E1 | 0.076019004 | 0.013450421 | 0.176934973 | A2F | 0.395732228 | 0.009904865 | 0.025029211 |
| H5N3F1 | 0.057499464 | 0.013416186 | 0.233327144 | A3F | 0.448321775 | 0.020320597 | 0.04532592 |
| H4N3E1 | 0.392010177 | 0.021838081 | 0.055707944 | A4F | 0.556221521 | 0.02854712 | 0.051323293 |
| H6N3 | 0.09135846 | 0.013155864 | 0.144002694 | A2Fa | 0.009854487 | 0.000638445 | 0.064787218 |
| H3N4F2 | 0.059534349 | 0.010787269 | 0.181194032 | A3Fa | 0.010346811 | 0.00141354 | 0.136616049 |
| H4N4F1 | 5.080758527 | 0.37481287 | 0.073771046 | A2S0F | 0.896163614 | 0.007482844 | 0.008349864 |
| H5N4 | 0.393766922 | 0.054741086 | 0.139019007 | A2L0F | 0.384038385 | 0.011099399 | 0.028901795 |
| H3N5F1 | 0.923986677 | 0.077000296 | 0.083334855 | A3L0F | 0.224215592 | 0.005530963 | 0.024668055 |
| H3N3F2L1 | 0.119062465 | 0.019410045 | 0.163024052 | A4L0F | 0.807602865 | 0.024087049 | 0.029825364 |
| H4N5 | 0.244791584 | 0.039401788 | 0.160960549 | A2E0F | 0.891993469 | 0.00658423 | 0.007381478 |
| H4N3F1L1 | 0.087120812 | 0.013450119 | 0.154384683 | A2SF | 0.276536338 | 0.003986308 | 0.014415132 |
| H5N3L1 | 0.047287876 | 0.009047412 | 0.191326251 | A2LF | 0.494604971 | 0.010597764 | 0.021426724 |
| H8N2 | 0.383977767 | 0.038621779 | 0.100583374 | A3LF | 0.546948991 | 0.028805176 | 0.052665196 |
| H4N3F1E1 | 0.100729097 | 0.007207029 | 0.071548627 | A4LF | 0.373671647 | 0.023645095 | 0.063277734 |
| H4N4L1 | 0.032585383 | 0.006509056 | 0.199753857 | A2EF | 0.256492891 | 0.003872188 | 0.015096668 |
| H5N3E1 | 0.277026178 | 0.019019548 | 0.068656139 | A3EF | 0.436141435 | 0.022286074 | 0.051098272 |
| H3N4F1E1 | 0.167696408 | 0.036022883 | 0.214810105 | A2B | 0.135329688 | 0.004873226 | 0.036010027 |
| H5N4F1 | 2.996973789 | 0.210080162 | 0.07009743 | A2F0B | 0.046662052 | 0.005986828 | 0.128301852 |
| H4N4E1 | 0.428495408 | 0.015326874 | 0.035769051 | A2FB | 0.27114578 | 0.00577779 | 0.021308796 |
| H4N5F1 | 1.596146621 | 0.083860992 | 0.052539655 | A2S0B | 0.24033785 | 0.006323956 | 0.026312776 |
| H5N5 | 0.17944697 | 0.019337809 | 0.107763362 | A2SB | 0.110467034 | 0.004502474 | 0.040758528 |
| H5N3F1L1 | 0.352451012 | 0.044116485 | 0.125170544 | A2F0S0B | 0.379274422 | 0.023926931 | 0.063086066 |
| H6N3L1 | 0.078911285 | 0.008164135 | 0.103459668 | A2F0SB | 0.035348255 | 0.00462745 | 0.130910286 |
| H9N2 | 0.543036184 | 0.060634909 | 0.111659058 | A2FS0B | 0.224362711 | 0.004968559 | 0.022145209 |
| H5N4L1 | 0.265903639 | 0.011632304 | 0.043746312 | A2FSB | 0.307040023 | 0.005745942 | 0.018713985 |
| H6N3E1 | 0.224749504 | 0.009683546 | 0.04308595 | A2G | 0.895715802 | 0.009040692 | 0.010093259 |
| H4N4F1E1 | 0.71101816 | 0.023274658 | 0.032734267 | A4G | 0.743469525 | 0.038891684 | 0.052311067 |
| H5N4E1 | 4.727910264 | 0.159005274 | 0.033631195 | A2F0G | 0.97790322 | 0.003144081 | 0.003215125 |
| H5N5F1 | 0.599817241 | 0.030722848 | 0.051220349 | A4F0G | 0.442683432 | 0.02860366 | 0.064614255 |

| | | | | | | | |
|---|---|---|---|---|---|---|---|
| H4N5E1 | 0.310326286 | 0.060089352 | 0.193632813 | A2FG | 0.770613216 | 0.014317192 | 0.01857896 |
| H5N4F1L1 | 0.565074338 | 0.019555364 | 0.034606711 | A2S0G | 0.503617859 | 0.012070807 | 0.023968186 |
| H4N4E1L1 | 0.088133361 | 0.00923248 | 0.104755793 | A2SG | 0.989179786 | 0.001271709 | 0.00128562 |
| H5N4F1E1 | 3.398623712 | 0.100344617 | 0.029525074 | A2F0S0G | 0.545446389 | 0.026553904 | 0.048682886 |
| H4N5F1E1 | 0.368827992 | 0.017663701 | 0.047891434 | A2FS0G | 0.498886436 | 0.010727887 | 0.021503665 |
| H3N4F2L2 | 0.087005775 | 0.048576333 | 0.558311594 | A2F0SG | 0.992761545 | 0.000835185 | 0.000841274 |
| H5N5E1 | 1.070173314 | 0.134134293 | 0.125338851 | A2FSG | 0.979867538 | 0.003638782 | 0.003713545 |
| H5N4E1L1 | 3.951900687 | 0.197733609 | 0.050035065 | A2S | 0.720584658 | 0.016349453 | 0.02268915 |
| H3N5F1E1L1 | 0.303957733 | 0.039803475 | 0.130950691 | A3S | 0.907182614 | 0.005016555 | 0.005529818 |
| H4N4F1E2 | 0.73316873 | 0.08892241 | 0.121285055 | A4S | 0.646919544 | 0.036395239 | 0.056259297 |
| H5N4E2 | 36.10552887 | 1.501930208 | 0.041598344 | A2F0S | 0.896868818 | 0.008578753 | 0.009565226 |
| H5N5F1E1 | 2.053852562 | 0.069539771 | 0.03385821 | A3F0S | 0.896886406 | 0.005551941 | 0.006190239 |
| H4N4F2L2 | 0.142568584 | 0.025570813 | 0.17935798 | A4F0S | 0.360942183 | 0.027383919 | 0.075867882 |
| H6N5E1 | 0.484571887 | 0.032329546 | 0.06671775 | A2FS | 0.452072148 | 0.019863928 | 0.043939729 |
| H5N4F1L2 | 0.993682761 | 0.043630936 | 0.043908316 | A3FS | 0.920236486 | 0.005203613 | 0.005654647 |
| H4N6F1E1 | 0.714082688 | 0.06051025 | 0.084738436 | A4FS | 0.286662755 | 0.012951608 | 0.045180643 |
| H4N4F2E1L1 | 0.437445058 | 0.025403968 | 0.058073505 | A2L | 0.061067336 | 0.001742998 | 0.028542235 |
| H5N4F1E1L1 | 1.641815195 | 0.072228285 | 0.043992945 | A3L | 0.270578151 | 0.004941553 | 0.018262942 |
| H4N7E1 | 0.545464254 | 0.034344452 | 0.062963709 | A4L | 0.293359804 | 0.029151934 | 0.099372625 |
| H5N4F1E2 | 3.641336889 | 0.179461848 | 0.049284604 | A2F0L | 0.044368542 | 0.001944699 | 0.043830579 |
| H6N5F1E1 | 0.230379094 | 0.014815902 | 0.064310967 | A3F0L | 0.21713866 | 0.005074971 | 0.02337203 |
| H5N5E2 | 0.290083893 | 0.016809416 | 0.057946741 | A4F0L | 0.169354609 | 0.015582436 | 0.092010699 |
| H4N5F2L2 | 0.018694696 | 0.007894997 | 0.422312123 | A2FL | 0.086608249 | 0.00364148 | 0.042045417 |
| H6N5E1L1 | 0.515358348 | 0.027196712 | 0.052772429 | A3FL | 0.336710099 | 0.003736993 | 0.011098547 |
| H5N5F1E2 | 2.749983681 | 0.107266273 | 0.039006149 | A4FL | 0.124563144 | 0.014749695 | 0.118411391 |
| H6N5E2 | 0.453898924 | 0.026301861 | 0.057946516 | A2E | 0.659726859 | 0.015896918 | 0.024096211 |
| H5N5F2L2 | 0.050780045 | 0.006546925 | 0.128927113 | A3E | 0.636472264 | 0.002824843 | 0.004438282 |
| H7N6E1 | 0.069402598 | 0.009018023 | 0.129937831 | A4E | 0.35334058 | 0.007542983 | 0.021347626 |
| H6N5F1L2 | 0.121106646 | 0.01052631 | 0.086917693 | A2F0E | 0.852550903 | 0.008460337 | 0.009923557 |
| H8N7 | 0.03497838 | 0.006929806 | 0.198116847 | A3F0E | 0.679718971 | 0.003597634 | 0.005292826 |
| H6N5F1E1L1 | 0.298133387 | 0.019025543 | 0.06381554 | A4F0E | 0.191599096 | 0.012266386 | 0.064021105 |
| H6N5F1E2 | 0.336879994 | 0.016778787 | 0.049806422 | A2FE | 0.365665753 | 0.017423363 | 0.047648332 |
| H6N5E1L2 | 0.495878411 | 0.040987047 | 0.082655438 | A3FE | 0.583893962 | 0.005150446 | 0.008820858 |
| H6N5E2L1 | 2.58653207 | 0.175155942 | 0.06771845 | A4FE | 0.161747657 | 0.005634238 | 0.034833506 |
| H6N5E3 | 1.740165337 | 0.081434013 | 0.04679671 | A2GS | 0.804224112 | 0.010987743 | 0.013662539 |
| H6N5F1L3 | 0.132816352 | 0.013976007 | 0.105228058 | A2F0GS | 0.917121124 | 0.006146379 | 0.006701819 |
| H7N6E2 | 0.059938347 | 0.008653509 | 0.144373494 | A4F0GS | 0.815025775 | 0.012271978 | 0.015057165 |
| H6N5F1E1L2 | 0.445317152 | 0.046887723 | 0.105290629 | A2FGS | 0.585897424 | 0.016306966 | 0.027832459 |
| H6N5F1E2L1 | 3.222202324 | 0.303259463 | 0.094115587 | A4FGS | 0.949115843 | 0.003975949 | 0.004189109 |
| H9N8 | 0.096374023 | 0.022683834 | 0.235372911 | A2GL | 0.068179656 | 0.001781861 | 0.02613479 |
| H6N5F1E3 | 0.208859186 | 0.019517426 | 0.093447775 | A2F0GL | 0.045367012 | 0.001959376 | 0.043189443 |
| H6N5F2E2L1 | 0.117479097 | 0.015536285 | 0.132247231 | A4F0GL | 0.382157522 | 0.013509023 | 0.035349357 |
| H7N6E1L2 | 0.173998724 | 0.031360898 | 0.180236369 | A2FGL | 0.11224599 | 0.003442029 | 0.030665052 |
| H7N6E2L1 | 0.153479848 | 0.029127813 | 0.189782653 | A4FGL | 0.411839745 | 0.031210124 | 0.075782205 |
| H7N6E3 | 0.039126807 | 0.008132235 | 0.207843062 | A2GE | 0.736255077 | 0.011317159 | 0.015371247 |
| H8N7E2 | 0.016049758 | 0.005668407 | 0.353177128 | A2F0GE | 0.87179351 | 0.006341055 | 0.007273574 |

| | | | | | | | |
|---|---|---|---|---|---|---|---|
| H7N6F1E1L2 | 0.133209274 | 0.028609585 | 0.214771722 | A4F0GE | 0.43271878 | 0.003577573 | 0.008267662 |
| H9N8E1 | 0.034674989 | 0.008469495 | 0.244253727 | A2FGE | 0.473933864 | 0.015400756 | 0.032495581 |
| H7N6E1L3 | 0.146979254 | 0.036200714 | 0.246298123 | A4FGE | 0.537104304 | 0.033911969 | 0.063138516 |
| H7N6E2L2 | 0.208953888 | 0.041337589 | 0.197831156 | | | | |
| H7N6E3L1 | 0.09646455 | 0.017750368 | 0.184009237 | | | | |
| H7N6F1E1L3 | 0.14015283 | 0.032972333 | 0.235259845 | | | | |
| H7N6F1E2L2 | 0.196518054 | 0.043297126 | 0.220321364 | | | | |
| H9N8E3 | 0.011044612 | 0.005922011 | 0.536189993 | | | | |

**Table S4. Derived *N*-glycan trait changes in the cohort.** IQR values of derived glycan traits in PTMC, HC, LNM, NLNM, CI, and NCI. The tendency of glycan trait alterations and p values for the comparison by Mann-Whitney U test in the cohort were listed. The p-values A considered significant are below the significance threshold of 5.49E-4 (=0.05/91, 91 derived glycan traits) after multiple testing corrections. The p-values B and C considered significant are below the significance threshold of 0.05 after multiple testing corrections. The p-values in bold indicated significance. PTMC, papillary thyroid microcarcinoma; HC, healthy controls; LNM, lymph node metastasis; NLNM, non-lymph node metastasis; CI, capsular invasion; NCI, non-capsular invasion.

| Traits | HC | | | PTMC | | | NLNM | | | LNM | | | NCI | | | CI | | | P value A | P value B | P value C |
|---|---|---|---|---|---|---|---|---|---|---|---|---|---|---|---|---|---|---|---|---|---|
| | 0.25 | median | 0.75 | 0.25 | median | 0.75 | 0.25 | median | 0.75 | 0.25 | median | 0.75 | 0.25 | median | 0.75 | 0.25 | median | 0.75 | PTMC vs. HC | LNM vs. NLNM | CI vs. NCI |
| TM | 0.0203 | 0.0227 | 0.0251 | 0.0249 | 0.0284 | 0.0313 | 0.0245 | 0.0275 | 0.0311 | 0.0255 | 0.0290 | 0.0318 | 0.0256 | 0.0280 | 0.0309 | 0.0247 | 0.0285 | 0.0323 | **1.37668E-14** | 0.254766991 | 0.5953069 |
| THy | 0.0126 | 0.0133 | 0.0143 | 0.0132 | 0.0143 | 0.0156 | 0.0133 | 0.0148 | 0.0161 | 0.0127 | 0.0141 | 0.0152 | 0.0129 | 0.0140 | 0.0159 | 0.0136 | 0.0145 | 0.0156 | 0.001085405 | 0.089223117 | 0.4946562 |
| MHy | 1.4634 | 1.7240 | 1.8755 | 1.7612 | 2.0062 | 2.2056 | 1.6373 | 1.9494 | 2.1057 | 1.8546 | 2.0865 | 2.2807 | 1.7944 | 2.0328 | 2.2948 | 1.7269 | 2.0013 | 2.1644 | **2.3644E-09** | **0.026546415** | 0.289732 |
| MM | 6.8731 | 6.9451 | 6.9894 | 6.8296 | 6.9005 | 6.9912 | 6.8119 | 6.8579 | 6.9492 | 6.8517 | 6.9325 | 7.0216 | 6.8019 | 6.8976 | 6.9888 | 6.8508 | 6.9034 | 6.9909 | 0.09328455 | **0.033418778** | 0.4079789 |
| CA1 | 0.0087 | 0.0090 | 0.0095 | 0.0090 | 0.0096 | 0.0103 | 0.0088 | 0.0093 | 0.0101 | 0.0092 | 0.0096 | 0.0103 | 0.0091 | 0.0097 | 0.0104 | 0.0089 | 0.0094 | 0.0102 | **3.61143E-05** | 0.191197124 | 0.3419551 |
| CA2 | 0.8460 | 0.8597 | 0.8717 | 0.8308 | 0.8475 | 0.8619 | 0.8371 | 0.8557 | 0.8660 | 0.8263 | 0.8413 | 0.8596 | 0.8345 | 0.8488 | 0.8633 | 0.8305 | 0.8468 | 0.8619 | 0.001096495 | **0.012975594** | 0.5803719 |
| CA3 | 0.0928 | 0.1061 | 0.1164 | 0.1009 | 0.1122 | 0.1270 | 0.0970 | 0.1051 | 0.1236 | 0.1039 | 0.1157 | 0.1296 | 0.1010 | 0.1100 | 0.1225 | 0.1006 | 0.1129 | 0.1294 | 0.021604219 | **0.048514552** | 0.4901007 |
| CA4 | 0.0163 | 0.0184 | 0.0211 | 0.0204 | 0.0224 | 0.0261 | 0.0188 | 0.0210 | 0.0231 | 0.0213 | 0.0234 | 0.0283 | 0.0204 | 0.0224 | 0.0266 | 0.0204 | 0.0225 | 0.0247 | **1.26061E-08** | **0.000634221** | 0.7151505 |
| CF | 0.3774 | 0.3963 | 0.4161 | 0.3739 | 0.3963 | 0.4220 | 0.3784 | 0.3965 | 0.4215 | 0.3731 | 0.3923 | 0.4217 | 0.3756 | 0.3977 | 0.4275 | 0.3734 | 0.3916 | 0.4215 | 0.84480109 | 0.671564731 | 0.3606244 |
| CFa | 0.0090 | 0.0095 | 0.0102 | 0.0084 | 0.0090 | 0.0096 | 0.0085 | 0.0090 | 0.0095 | 0.0083 | 0.0090 | 0.0096 | 0.0083 | 0.0090 | 0.0094 | 0.0084 | 0.0091 | 0.0097 | **7.73242E-05** | 0.814381291 | 0.3878137 |
| CB | 0.0815 | 0.0900 | 0.0992 | 0.0851 | 0.0962 | 0.1045 | 0.0861 | 0.0972 | 0.1047 | 0.0851 | 0.0958 | 0.1041 | 0.0841 | 0.0973 | 0.1081 | 0.0852 | 0.0948 | 0.1040 | 0.041246356 | 0.787299408 | 0.6104148 |
| CG | 0.9417 | 0.9507 | 0.9630 | 0.9417 | 0.9556 | 0.9646 | 0.9410 | 0.9559 | 0.9680 | 0.9428 | 0.9547 | 0.9618 | 0.9384 | 0.9496 | 0.9590 | 0.9522 | 0.9585 | 0.9676 | 0.346522232 | 0.33875331 | **0.0044593** |
| CS | 0.7899 | 0.8061 | 0.8302 | 0.7871 | 0.8114 | 0.8389 | 0.7807 | 0.8100 | 0.8418 | 0.7928 | 0.8115 | 0.8345 | 0.7698 | 0.7970 | 0.8345 | 0.7940 | 0.8159 | 0.8406 | 0.808922484 | 0.825278451 | **0.0377395** |
| A2F | 0.3836 | 0.4050 | 0.4314 | 0.3867 | 0.4089 | 0.4387 | 0.3828 | 0.4132 | 0.4444 | 0.3874 | 0.4055 | 0.4305 | 0.3880 | 0.4120 | 0.4476 | 0.3871 | 0.4087 | 0.4272 | 0.469943965 | 0.66137319 | 0.3799207 |
| A3F | 0.2579 | 0.2974 | 0.3548 | 0.2245 | 0.2774 | 0.3622 | 0.2359 | 0.2785 | 0.3412 | 0.2152 | 0.2762 | 0.3623 | 0.2276 | 0.2846 | 0.3502 | 0.2201 | 0.2762 | 0.3621 | 0.241334512 | 0.739214505 | 0.73684 |
| A4F | 0.4485 | 0.4992 | 0.5441 | 0.4065 | 0.4678 | 0.5129 | 0.4324 | 0.4812 | 0.5115 | 0.3900 | 0.4472 | 0.5144 | 0.3946 | 0.4698 | 0.5127 | 0.4077 | 0.4620 | 0.5125 | 0.012261549 | 0.18882797 | 0.9222815 |
| A2Fa | 0.0088 | 0.0094 | 0.0100 | 0.0082 | 0.0087 | 0.0092 | 0.0082 | 0.0087 | 0.0092 | 0.0083 | 0.0088 | 0.0093 | 0.0080 | 0.0087 | 0.0089 | 0.0083 | 0.0088 | 0.0094 | **3.93736E-06** | 0.494412128 | 0.1555949 |
| A3Fa | 0.0037 | 0.0053 | 0.0070 | 0.0033 | 0.0052 | 0.0077 | 0.0033 | 0.0052 | 0.0077 | 0.0033 | 0.0051 | 0.0076 | 0.0035 | 0.0052 | 0.0083 | 0.0032 | 0.0052 | 0.0073 | 0.951793986 | 0.986021201 | 0.2997221 |
| A2S0F | 0.8975 | 0.9153 | 0.9256 | 0.8870 | 0.9065 | 0.9188 | 0.8818 | 0.9024 | 0.9186 | 0.8914 | 0.9072 | 0.9182 | 0.8899 | 0.9129 | 0.9231 | 0.8852 | 0.9046 | 0.9141 | 0.016877036 | 0.379108409 | 0.1031823 |
| A2L0F | 0.3744 | 0.3988 | 0.4260 | 0.3779 | 0.4030 | 0.4388 | 0.3760 | 0.4102 | 0.4464 | 0.3791 | 0.4004 | 0.4262 | 0.3787 | 0.4063 | 0.4481 | 0.3785 | 0.4012 | 0.4208 | 0.364506624 | 0.503309485 | 0.2641983 |

| | | | | | | | | | | | | | | | | | | | | | |
|---|---|---|---|---|---|---|---|---|---|---|---|---|---|---|---|---|---|---|---|---|---|
| A3L0F | 0.1647 | 0.1865 | 0.1977 | 0.1568 | 0.1787 | 0.2052 | 0.1698 | 0.1823 | 0.2050 | 0.1515 | 0.1764 | 0.2054 | 0.1553 | 0.1847 | 0.2035 | 0.1595 | 0.1767 | 0.2067 | 0.682697632 | 0.291540824 | 0.6104148 |
| A4L0F | 0.7696 | 0.8024 | 0.8350 | 0.7703 | 0.8082 | 0.8343 | 0.7734 | 0.8161 | 0.8370 | 0.7649 | 0.7979 | 0.8292 | 0.7888 | 0.8082 | 0.8281 | 0.7542 | 0.8048 | 0.8359 | 0.951793986 | 0.257702266 | 0.6883604 |
| A2E0F | 0.8906 | 0.9077 | 0.9173 | 0.8812 | 0.9003 | 0.9106 | 0.8765 | 0.8945 | 0.9116 | 0.8857 | 0.9010 | 0.9100 | 0.8839 | 0.9034 | 0.9144 | 0.8796 | 0.8989 | 0.9082 | 0.011771536 | 0.321357119 | 0.1473513 |
| A2SF | 0.2455 | 0.2623 | 0.2767 | 0.2482 | 0.2704 | 0.2903 | 0.2557 | 0.2729 | 0.2936 | 0.2471 | 0.2670 | 0.2868 | 0.2462 | 0.2665 | 0.2813 | 0.2541 | 0.2731 | 0.2911 | 0.036361406 | 0.31456497 | 0.3760115 |
| A2LF | 0.4364 | 0.4609 | 0.4907 | 0.4289 | 0.4498 | 0.4817 | 0.4216 | 0.4436 | 0.4751 | 0.4306 | 0.4555 | 0.4835 | 0.4231 | 0.4447 | 0.4783 | 0.4310 | 0.4555 | 0.4840 | 0.234467097 | 0.328244335 | 0.283198 |
| A3LF | 0.2913 | 0.3518 | 0.4248 | 0.2509 | 0.3293 | 0.4333 | 0.2641 | 0.3248 | 0.4204 | 0.2358 | 0.3314 | 0.4356 | 0.2539 | 0.3493 | 0.4269 | 0.2514 | 0.3262 | 0.4415 | 0.206309645 | 0.88022956 | 0.7043901 |
| A4LF | 0.1850 | 0.2283 | 0.2718 | 0.1546 | 0.2086 | 0.2750 | 0.1605 | 0.2023 | 0.2706 | 0.1397 | 0.2131 | 0.2788 | 0.1560 | 0.2095 | 0.2679 | 0.1549 | 0.2025 | 0.2806 | 0.166148387 | 0.852664611 | 0.8311809 |
| A2EF | 0.2198 | 0.2386 | 0.2541 | 0.2255 | 0.2488 | 0.2687 | 0.2324 | 0.2504 | 0.2690 | 0.2218 | 0.2454 | 0.2655 | 0.2224 | 0.2425 | 0.2597 | 0.2299 | 0.2504 | 0.2688 | 0.02193465 | 0.321357119 | 0.4588539 |
| A3EF | 0.2371 | 0.2790 | 0.3348 | 0.2059 | 0.2591 | 0.3460 | 0.2186 | 0.2611 | 0.3204 | 0.1909 | 0.2571 | 0.3460 | 0.2081 | 0.2696 | 0.3344 | 0.2000 | 0.2571 | 0.3460 | 0.262781986 | 0.739214505 | 0.7043901 |
| A2B | 0.0952 | 0.1032 | 0.1159 | 0.1010 | 0.1130 | 0.1235 | 0.1019 | 0.1129 | 0.1226 | 0.1006 | 0.1132 | 0.1246 | 0.0997 | 0.1132 | 0.1280 | 0.1015 | 0.1129 | 0.1220 | 0.005373169 | 0.974841045 | 0.7043901 |
| A2F0B | 0.0278 | 0.0306 | 0.0355 | 0.0316 | 0.0342 | 0.0393 | 0.0316 | 0.0336 | 0.0393 | 0.0314 | 0.0357 | 0.0389 | 0.0305 | 0.0343 | 0.0393 | 0.0318 | 0.0342 | 0.0385 | **0.000206663** | 0.697305707 | 0.791954 |
| A2FB | 0.1932 | 0.2142 | 0.2328 | 0.2014 | 0.2266 | 0.2460 | 0.2002 | 0.2284 | 0.2424 | 0.2018 | 0.2264 | 0.2467 | 0.2024 | 0.2174 | 0.2550 | 0.2002 | 0.2268 | 0.2444 | 0.046994648 | 0.874703837 | 0.9855856 |
| A2S0B | 0.1701 | 0.1928 | 0.2076 | 0.1748 | 0.1934 | 0.2144 | 0.1738 | 0.1973 | 0.2042 | 0.1754 | 0.1909 | 0.2158 | 0.1728 | 0.1947 | 0.2157 | 0.1778 | 0.1931 | 0.2063 | 0.480625033 | 0.68181702 | 0.8481282 |
| A2SB | 0.0719 | 0.0795 | 0.0931 | 0.0780 | 0.0904 | 0.1005 | 0.0791 | 0.0897 | 0.1006 | 0.0778 | 0.0911 | 0.0981 | 0.0769 | 0.0896 | 0.1023 | 0.0791 | 0.0926 | 0.0986 | 0.001588522 | 0.825278451 | 0.7808378 |
| A2F0S0B | 0.2555 | 0.2802 | 0.3173 | 0.2580 | 0.2829 | 0.3098 | 0.2569 | 0.2750 | 0.3066 | 0.2608 | 0.2907 | 0.3122 | 0.2596 | 0.2850 | 0.3119 | 0.2577 | 0.2778 | 0.3083 | 0.723272785 | 0.275806128 | 0.6567236 |
| A2F0SB | 0.0196 | 0.0222 | 0.0257 | 0.0231 | 0.0254 | 0.0288 | 0.0225 | 0.0251 | 0.0285 | 0.0231 | 0.0261 | 0.0294 | 0.0227 | 0.0256 | 0.0284 | 0.0232 | 0.0254 | 0.0288 | **0.000121464** | 0.676683385 | 0.9395144 |
| A2FS0B | 0.1622 | 0.1834 | 0.1973 | 0.1658 | 0.1822 | 0.1986 | 0.1652 | 0.1848 | 0.1950 | 0.1664 | 0.1797 | 0.2059 | 0.1623 | 0.1891 | 0.2092 | 0.1668 | 0.1797 | 0.1967 | 0.643019414 | 0.781912629 | 0.7477652 |
| A2FSB | 0.2200 | 0.2445 | 0.2737 | 0.2273 | 0.2608 | 0.2945 | 0.2245 | 0.2601 | 0.2938 | 0.2337 | 0.2628 | 0.2952 | 0.2273 | 0.2679 | 0.2952 | 0.2325 | 0.2594 | 0.2938 | 0.00613299 | 0.88022956 | 0.8481282 |
| A2G | 0.8703 | 0.8837 | 0.9020 | 0.8641 | 0.8886 | 0.9045 | 0.8634 | 0.8919 | 0.9123 | 0.8674 | 0.8878 | 0.9014 | 0.8609 | 0.8762 | 0.8988 | 0.8726 | 0.8919 | 0.9113 | 0.76244593 | 0.494412128 | **0.0120296** |
| A4G | 0.6846 | 0.7203 | 0.7749 | 0.6951 | 0.7478 | 0.7905 | 0.6833 | 0.7314 | 0.7804 | 0.7071 | 0.7660 | 0.7930 | 0.6958 | 0.7580 | 0.7954 | 0.6954 | 0.7458 | 0.7894 | 0.019863221 | 0.153814426 | 0.5462238 |
| A2F0G | 0.9776 | 0.9800 | 0.9832 | 0.9753 | 0.9790 | 0.9820 | 0.9752 | 0.9794 | 0.9826 | 0.9754 | 0.9781 | 0.9816 | 0.9739 | 0.9774 | 0.9806 | 0.9755 | 0.9794 | 0.9826 | 0.031520578 | 0.601588502 | 0.1598457 |
| A4F0G | 0.4551 | 0.5000 | 0.5503 | 0.4848 | 0.5283 | 0.5908 | 0.4877 | 0.5164 | 0.5662 | 0.4794 | 0.5516 | 0.6080 | 0.4814 | 0.5235 | 0.6035 | 0.4849 | 0.5369 | 0.5881 | 0.015847633 | 0.210941926 | 0.9337665 |
| A2FG | 0.7178 | 0.7450 | 0.7814 | 0.7218 | 0.7607 | 0.7853 | 0.7187 | 0.7641 | 0.8032 | 0.7232 | 0.7596 | 0.7791 | 0.7088 | 0.7334 | 0.7718 | 0.7428 | 0.7668 | 0.8043 | 0.241334512 | 0.237643764 | **0.0030116** |
| A2S0G | 0.4759 | 0.5156 | 0.5497 | 0.4958 | 0.5252 | 0.5684 | 0.4971 | 0.5447 | 0.5757 | 0.4940 | 0.5210 | 0.5534 | 0.4872 | 0.5105 | 0.5359 | 0.5071 | 0.5447 | 0.5764 | 0.167030782 | 0.179567678 | **0.0144357** |
| A2SG | 0.9889 | 0.9898 | 0.9914 | 0.9873 | 0.9895 | 0.9907 | 0.9879 | 0.9893 | 0.9906 | 0.9873 | 0.9898 | 0.9910 | 0.9865 | 0.9883 | 0.9906 | 0.9881 | 0.9897 | 0.9909 | 0.017549814 | 0.666461264 | 0.1731252 |
| A2F0S0G | 0.5469 | 0.5782 | 0.6032 | 0.5511 | 0.5904 | 0.6093 | 0.5737 | 0.5986 | 0.6219 | 0.5435 | 0.5783 | 0.5983 | 0.5395 | 0.5748 | 0.5957 | 0.5605 | 0.5942 | 0.6177 | 0.477050052 | **0.007347482** | 0.05688 |

| | | | | | | | | | | | | | | | | | | | | | |
|---|---|---|---|---|---|---|---|---|---|---|---|---|---|---|---|---|---|---|---|---|---|
| A2FS0G | 0.4692 | 0.5082 | 0.5429 | 0.4890 | 0.5178 | 0.5656 | 0.4908 | 0.5334 | 0.5716 | 0.4884 | 0.5170 | 0.5511 | 0.4813 | 0.5066 | 0.5301 | 0.4958 | 0.5334 | 0.5731 | 0.171495646 | 0.25185505 | **0.0138671** |
| A2F0SG | 0.9934 | 0.9939 | 0.9946 | 0.9927 | 0.9936 | 0.9946 | 0.9929 | 0.9938 | 0.9945 | 0.9925 | 0.9934 | 0.9946 | 0.9925 | 0.9932 | 0.9945 | 0.9929 | 0.9939 | 0.9946 | 0.089418741 | 0.902388173 | 0.1433568 |
| A2FSG | 0.9756 | 0.9792 | 0.9829 | 0.9726 | 0.9777 | 0.9820 | 0.9721 | 0.9778 | 0.9821 | 0.9728 | 0.9777 | 0.9820 | 0.9698 | 0.9752 | 0.9813 | 0.9739 | 0.9783 | 0.9824 | 0.024740069 | 0.760472347 | 0.1535019 |
| A2S | 0.6691 | 0.6883 | 0.7122 | 0.6553 | 0.6870 | 0.7127 | 0.6531 | 0.6868 | 0.7194 | 0.6592 | 0.6872 | 0.7121 | 0.6420 | 0.6712 | 0.7107 | 0.6697 | 0.6923 | 0.7129 | 0.421873205 | 0.836209382 | 0.0669566 |
| A3S | 0.8921 | 0.8992 | 0.9078 | 0.8963 | 0.9029 | 0.9111 | 0.8923 | 0.9001 | 0.9087 | 0.8988 | 0.9056 | 0.9127 | 0.8977 | 0.9035 | 0.9129 | 0.8946 | 0.9016 | 0.9105 | 0.039559657 | **0.035818439** | 0.3721272 |
| A4S | 0.5848 | 0.6174 | 0.6680 | 0.6046 | 0.6516 | 0.6896 | 0.5954 | 0.6374 | 0.6820 | 0.6188 | 0.6569 | 0.6957 | 0.6061 | 0.6722 | 0.6981 | 0.6039 | 0.6435 | 0.6820 | 0.00546931 | 0.210941926 | 0.2641983 |
| A2F0S | 0.8907 | 0.8981 | 0.9051 | 0.8803 | 0.8918 | 0.9009 | 0.8805 | 0.8895 | 0.8995 | 0.8812 | 0.8918 | 0.9022 | 0.8788 | 0.8908 | 0.8978 | 0.8835 | 0.8918 | 0.9024 | 0.00176997 | 0.692128393 | 0.3493474 |
| A3F0S | 0.8902 | 0.9021 | 0.9122 | 0.8974 | 0.9061 | 0.9156 | 0.8943 | 0.9055 | 0.9128 | 0.8975 | 0.9085 | 0.9166 | 0.8994 | 0.9080 | 0.9171 | 0.8971 | 0.9046 | 0.9154 | 0.066261195 | 0.181850474 | 0.3799207 |
| A4F0S | 0.3628 | 0.4083 | 0.4629 | 0.3951 | 0.4412 | 0.5004 | 0.4026 | 0.4270 | 0.4723 | 0.3915 | 0.4656 | 0.5064 | 0.3947 | 0.4392 | 0.5193 | 0.4060 | 0.4431 | 0.4940 | 0.005869592 | 0.257702266 | 0.7422961 |
| A2FS | 0.3622 | 0.3918 | 0.4182 | 0.3574 | 0.3945 | 0.4242 | 0.3575 | 0.3995 | 0.4241 | 0.3581 | 0.3897 | 0.4221 | 0.3413 | 0.3657 | 0.4162 | 0.3630 | 0.4026 | 0.4276 | 0.771238436 | 0.749819673 | **0.0159481** |
| A3FS | 0.8787 | 0.8931 | 0.9068 | 0.8734 | 0.8966 | 0.9084 | 0.8697 | 0.8942 | 0.9073 | 0.8787 | 0.8969 | 0.9113 | 0.8854 | 0.8973 | 0.9120 | 0.8707 | 0.8958 | 0.9081 | 0.500545807 | 0.345877931 | 0.3493474 |
| A4FS | 0.1846 | 0.2082 | 0.2287 | 0.1729 | 0.1956 | 0.2339 | 0.1791 | 0.1954 | 0.2327 | 0.1635 | 0.1998 | 0.2373 | 0.1756 | 0.2063 | 0.2292 | 0.1730 | 0.1944 | 0.2369 | 0.270215108 | 0.651244028 | 0.8311809 |
| A2L | 0.0573 | 0.0618 | 0.0645 | 0.0560 | 0.0594 | 0.0643 | 0.0555 | 0.0594 | 0.0645 | 0.0562 | 0.0594 | 0.0625 | 0.0566 | 0.0587 | 0.0630 | 0.0560 | 0.0598 | 0.0644 | 0.09328455 | 0.666461264 | 0.6205803 |
| A3L | 0.2603 | 0.2700 | 0.2783 | 0.2625 | 0.2717 | 0.2803 | 0.2625 | 0.2722 | 0.2802 | 0.2622 | 0.2716 | 0.2803 | 0.2679 | 0.2725 | 0.2826 | 0.2609 | 0.2696 | 0.2793 | 0.336296909 | 0.841686872 | 0.117682 |
| A4L | 0.2354 | 0.2694 | 0.3030 | 0.2592 | 0.2924 | 0.3231 | 0.2589 | 0.2837 | 0.3175 | 0.2635 | 0.2988 | 0.3276 | 0.2562 | 0.3123 | 0.3363 | 0.2593 | 0.2863 | 0.3175 | 0.005973702 | 0.285175993 | 0.16861 |
| A2F0L | 0.0437 | 0.0472 | 0.0506 | 0.0433 | 0.0471 | 0.0509 | 0.0457 | 0.0494 | 0.0522 | 0.0425 | 0.0457 | 0.0500 | 0.0440 | 0.0485 | 0.0514 | 0.0431 | 0.0467 | 0.0508 | 0.979330007 | 0.053509817 | 0.3099393 |
| A3F0L | 0.2206 | 0.2378 | 0.2496 | 0.2229 | 0.2436 | 0.2543 | 0.2249 | 0.2459 | 0.2521 | 0.2215 | 0.2385 | 0.2554 | 0.2289 | 0.2459 | 0.2567 | 0.2214 | 0.2414 | 0.2517 | 0.103842758 | 0.68181702 | 0.2550357 |
| A4F0L | 0.1690 | 0.1929 | 0.2291 | 0.1891 | 0.2166 | 0.2475 | 0.1893 | 0.2078 | 0.2356 | 0.1853 | 0.2242 | 0.2576 | 0.1894 | 0.2133 | 0.2660 | 0.1889 | 0.2167 | 0.2424 | 0.003920066 | 0.321357119 | 0.541428 |
| A2FL | 0.0742 | 0.0820 | 0.0912 | 0.0700 | 0.0789 | 0.0880 | 0.0690 | 0.0790 | 0.0880 | 0.0725 | 0.0789 | 0.0873 | 0.0680 | 0.0776 | 0.0855 | 0.0722 | 0.0815 | 0.0890 | 0.070627521 | 0.66137319 | 0.258065 |
| A3FL | 0.3356 | 0.3456 | 0.3539 | 0.3368 | 0.3460 | 0.3530 | 0.3377 | 0.3445 | 0.3528 | 0.3362 | 0.3462 | 0.3531 | 0.3355 | 0.3462 | 0.3520 | 0.3377 | 0.3457 | 0.3532 | 0.740600621 | 0.666461264 | 0.753247 |
| A4FL | 0.0585 | 0.0684 | 0.0835 | 0.0511 | 0.0698 | 0.0898 | 0.0510 | 0.0721 | 0.0866 | 0.0516 | 0.0688 | 0.0935 | 0.0533 | 0.0725 | 0.0896 | 0.0479 | 0.0688 | 0.0900 | 0.981626242 | 0.913497171 | 0.6359649 |
| A2E | 0.6072 | 0.6279 | 0.6482 | 0.5965 | 0.6271 | 0.6523 | 0.5975 | 0.6269 | 0.6542 | 0.5957 | 0.6292 | 0.6518 | 0.5867 | 0.6101 | 0.6505 | 0.6098 | 0.6319 | 0.6526 | 0.616437802 | 0.755140189 | 0.0578272 |
| A3E | 0.6222 | 0.6289 | 0.6396 | 0.6210 | 0.6334 | 0.6431 | 0.6208 | 0.6293 | 0.6367 | 0.6234 | 0.6364 | 0.6444 | 0.6209 | 0.6311 | 0.6391 | 0.6214 | 0.6335 | 0.6439 | 0.477050052 | 0.054382751 | 0.4120856 |
| A4E | 0.3453 | 0.3536 | 0.3631 | 0.3456 | 0.3569 | 0.3678 | 0.3429 | 0.3509 | 0.3675 | 0.3485 | 0.3611 | 0.3680 | 0.3458 | 0.3551 | 0.3666 | 0.3455 | 0.3579 | 0.3683 | 0.116664859 | 0.106221804 | 0.6359649 |
| A2F0E | 0.8414 | 0.8495 | 0.8581 | 0.8313 | 0.8464 | 0.8545 | 0.8318 | 0.8476 | 0.8522 | 0.8311 | 0.8460 | 0.8584 | 0.8265 | 0.8460 | 0.8543 | 0.8341 | 0.8468 | 0.8554 | 0.02032545 | 0.360411262 | 0.3382968 |
| A3F0E | 0.6544 | 0.6641 | 0.6770 | 0.6524 | 0.6645 | 0.6789 | 0.6521 | 0.6616 | 0.6693 | 0.6531 | 0.6703 | 0.6804 | 0.6508 | 0.6614 | 0.6725 | 0.6529 | 0.6650 | 0.6797 | 0.820095224 | 0.145885331 | 0.4120856 |

| | | | | | | | | | | | | | | | | | | | | | |
|---|---|---|---|---|---|---|---|---|---|---|---|---|---|---|---|---|---|---|---|---|---|
| A4F0E | 0.1959 | 0.2163 | 0.2397 | 0.2064 | 0.2230 | 0.2532 | 0.2068 | 0.2176 | 0.2452 | 0.2061 | 0.2297 | 0.2574 | 0.2062 | 0.2221 | 0.2544 | 0.2068 | 0.2231 | 0.2512 | 0.02996222 | 0.221349931 | 0.8424709 |
| A2FE | 0.2797 | 0.3067 | 0.3319 | 0.2846 | 0.3152 | 0.3391 | 0.2857 | 0.3245 | 0.3394 | 0.2843 | 0.3126 | 0.3370 | 0.2703 | 0.2907 | 0.3318 | 0.2880 | 0.3251 | 0.3460 | 0.245990737 | 0.582216024 | **0.0147277** |
| A3FE | 0.5346 | 0.5463 | 0.5649 | 0.5289 | 0.5487 | 0.5667 | 0.5282 | 0.5483 | 0.5641 | 0.5292 | 0.5492 | 0.5696 | 0.5304 | 0.5517 | 0.5660 | 0.5282 | 0.5483 | 0.5695 | 0.970146972 | 0.636170725 | 0.8199256 |
| A4FE | 0.1214 | 0.1393 | 0.1502 | 0.1139 | 0.1294 | 0.1432 | 0.1214 | 0.1297 | 0.1432 | 0.1079 | 0.1262 | 0.1444 | 0.1088 | 0.1315 | 0.1440 | 0.1155 | 0.1293 | 0.1423 | 0.027444648 | 0.328244335 | 0.8993623 |
| A2GS | 0.7652 | 0.7822 | 0.7935 | 0.7564 | 0.7773 | 0.7981 | 0.7556 | 0.7683 | 0.7931 | 0.7574 | 0.7778 | 0.8009 | 0.7449 | 0.7661 | 0.7932 | 0.7598 | 0.7784 | 0.8007 | 0.23220902 | 0.39060793 | 0.258065 |
| A2F0GS | 0.9098 | 0.9154 | 0.9209 | 0.9024 | 0.9117 | 0.9182 | 0.9023 | 0.9117 | 0.9160 | 0.9038 | 0.9117 | 0.9192 | 0.9018 | 0.9118 | 0.9158 | 0.9039 | 0.9117 | 0.9183 | 0.002008884 | 0.516812447 | 0.6777507 |
| A4F0GS | 0.7984 | 0.8239 | 0.8390 | 0.8149 | 0.8327 | 0.8445 | 0.8178 | 0.8336 | 0.8410 | 0.8116 | 0.8327 | 0.8511 | 0.8214 | 0.8363 | 0.8568 | 0.8134 | 0.8306 | 0.8411 | 0.00546931 | 0.712921574 | 0.0847864 |
| A2FGS | 0.4976 | 0.5221 | 0.5467 | 0.4898 | 0.5214 | 0.5505 | 0.4887 | 0.5199 | 0.5565 | 0.4951 | 0.5253 | 0.5481 | 0.4804 | 0.5105 | 0.5476 | 0.4984 | 0.5276 | 0.5536 | 0.915174281 | 0.930191731 | 0.117682 |
| A4FGS | 0.9590 | 0.9633 | 0.9683 | 0.9560 | 0.9622 | 0.9690 | 0.9559 | 0.9639 | 0.9708 | 0.9561 | 0.9613 | 0.9686 | 0.9577 | 0.9629 | 0.9670 | 0.9559 | 0.9617 | 0.9696 | 0.590349786 | 0.530500705 | 0.870833 |
| A2GL | 0.0660 | 0.0693 | 0.0726 | 0.0637 | 0.0677 | 0.0707 | 0.0639 | 0.0681 | 0.0712 | 0.0634 | 0.0667 | 0.0706 | 0.0650 | 0.0676 | 0.0699 | 0.0628 | 0.0680 | 0.0712 | 0.020481598 | 0.521354785 | 0.9740573 |
| A2F0GL | 0.0446 | 0.0480 | 0.0516 | 0.0442 | 0.0481 | 0.0518 | 0.0465 | 0.0504 | 0.0532 | 0.0435 | 0.0468 | 0.0511 | 0.0451 | 0.0495 | 0.0526 | 0.0441 | 0.0476 | 0.0516 | 0.906044363 | 0.06581584 | 0.2864524 |
| A4F0GL | 0.3700 | 0.3943 | 0.4097 | 0.3802 | 0.4037 | 0.4253 | 0.3825 | 0.4078 | 0.4237 | 0.3784 | 0.4032 | 0.4274 | 0.3865 | 0.4150 | 0.4390 | 0.3784 | 0.4013 | 0.4168 | 0.02127813 | 0.858164342 | 0.0943316 |
| A2FGL | 0.1003 | 0.1108 | 0.1199 | 0.0928 | 0.1051 | 0.1148 | 0.0896 | 0.1036 | 0.1129 | 0.0936 | 0.1071 | 0.1163 | 0.0912 | 0.1061 | 0.1142 | 0.0930 | 0.1043 | 0.1147 | 0.010577847 | 0.33875331 | 0.7205521 |
| A4FGL | 0.2940 | 0.3184 | 0.3708 | 0.2739 | 0.3441 | 0.3954 | 0.2670 | 0.3358 | 0.3948 | 0.2744 | 0.3501 | 0.3972 | 0.2971 | 0.3416 | 0.3988 | 0.2626 | 0.3467 | 0.3876 | 0.342115946 | 0.626204413 | 0.3419551 |
| A2GE | 0.6960 | 0.7112 | 0.7228 | 0.6869 | 0.7105 | 0.7285 | 0.6877 | 0.7058 | 0.7250 | 0.6851 | 0.7143 | 0.7295 | 0.6784 | 0.6990 | 0.7265 | 0.6900 | 0.7113 | 0.7285 | 0.44553917 | 0.328244335 | 0.2460963 |
| A2F0GE | 0.8593 | 0.8668 | 0.8746 | 0.8501 | 0.8628 | 0.8730 | 0.8493 | 0.8605 | 0.8703 | 0.8514 | 0.8656 | 0.8749 | 0.8473 | 0.8654 | 0.8721 | 0.8550 | 0.8625 | 0.8739 | 0.039010595 | 0.237643764 | 0.4038966 |
| A4F0GE | 0.4208 | 0.4276 | 0.4374 | 0.4183 | 0.4261 | 0.4375 | 0.4169 | 0.4223 | 0.4330 | 0.4189 | 0.4290 | 0.4382 | 0.4158 | 0.4251 | 0.4350 | 0.4200 | 0.4276 | 0.4379 | 0.43700139 | 0.196000919 | 0.2963668 |
| A2FGE | 0.3889 | 0.4093 | 0.4282 | 0.3899 | 0.4178 | 0.4414 | 0.3878 | 0.4174 | 0.4470 | 0.3949 | 0.4183 | 0.4372 | 0.3764 | 0.4059 | 0.4399 | 0.3959 | 0.4217 | 0.4435 | 0.257905713 | 0.825278451 | 0.1001615 |
| A4FGE | 0.5885 | 0.6447 | 0.6681 | 0.5630 | 0.6174 | 0.6939 | 0.5619 | 0.6265 | 0.6972 | 0.5635 | 0.6098 | 0.6917 | 0.5577 | 0.6192 | 0.6598 | 0.5671 | 0.6156 | 0.7027 | 0.32062506 | 0.601588502 | 0.3998389 |
